# Measuring the accuracy of gridded human population density surfaces: A case study in Bioko Island, Equatorial Guinea

**DOI:** 10.1371/journal.pone.0248646

**Published:** 2021-09-01

**Authors:** Brendan Fries, Carlos A. Guerra, Guillermo A. García, Sean L. Wu, Jordan M. Smith, Jeremías Nzamio Mba Oyono, Olivier T. Donfack, José Osá Osá Nfumu, Simon I. Hay, David L. Smith, Andrew J. Dolgert

**Affiliations:** 1 South and Central Africa ICEMR, Johns Hopkins Bloomberg School of Public Health, Baltimore, MD, United States of America; 2 Spatial Science for Public Health Center, Johns Hopkins Bloomberg School of Public Health, Baltimore, MD, United States of America; 3 Medical Care Development International, Silver Spring, MD, United States of America; 4 Divisions of Biostatistics & Epidemiology, University of California, Berkeley, Berkeley, CA, United States of America; 5 Medical Care Development International, Malabo, Equatorial Guinea; 6 Ministry of Health and Social Welfare, Malabo, Equatorial Guinea; 7 Department of Health Metrics Sciences, School of Medicine, University of Washington, Seattle, WA, United States of America; 8 Institute for Health Metrics and Evaluation, University of Washington, Seattle, WA, United States of America; Northeastern University (Shenyang China), CHINA

## Abstract

**Background:**

Geospatial datasets of population are becoming more common in models used for health policy. Publicly-available maps of human population make a consistent picture from inconsistent census data, and the techniques they use to impute data makes each population map unique. Each mapping model explains its methods, but it can be difficult to know which map is appropriate for which policy work. High quality census datasets, where available, are a unique opportunity to characterize maps by comparing them with truth.

**Methods:**

We use census data from a bed-net mass-distribution campaign on Bioko Island, Equatorial Guinea, conducted by the Bioko Island Malaria Elimination Program as a gold standard to evaluate LandScan (LS), WorldPop Constrained (WP-C) and WorldPop Unconstrained (WP-U), Gridded Population of the World (GPW), and the High-Resolution Settlement Layer (HRSL). Each layer is compared to the gold-standard using statistical measures to evaluate distribution, error, and bias. We investigated how map choice affects burden estimates from a malaria prevalence model.

**Results:**

Specific population layers were able to match the gold-standard distribution at different population densities. LandScan was able to most accurately capture highly urban distribution, HRSL and WP-C matched best at all other lower population densities. GPW and WP-U performed poorly everywhere. Correctly capturing empty pixels is key, and smaller pixel sizes (100 m vs 1 km) improve this. Normalizing areas based on known district populations increased performance. The use of differing population layers in a malaria model showed a disparity in results around transition points between endemicity levels.

**Discussion:**

The metrics in this paper, some of them novel in this context, characterize how these population maps differ from the gold standard census and from each other. We show that the metrics help understand the performance of a population map within a malaria model. The closest match to the census data would combine LandScan within urban areas and the HRSL for rural areas. Researchers should prefer particular maps if health calculations have a strong dependency on knowing where people are not, or if it is important to categorize variation in density within a city.

## Introduction

The premise of precision public health is that evidence can be used to improve the efficiency and effectiveness of interventions to benefit those most in need [1, 2]. Data accurately describing the geographical distribution of humans are among the most important components of evidence, along with observational or intervention studies, as they identify populations at risk, and resource needs scale with population size [3, 4]. Advances in GIS and satellite imagery have led to the creation of maps for disease risk and spread [5, 6]. These disease models incorporate population as covariates for mapping prevalence, incidence, and other metrics [7–10]. They inform public health in areas without first-rate census data, as in most of the developing world, where much of the infectious disease burden resides. Making effective policy thus requires having accurate maps of human populations [3, 11].

The last two decades, since the advent of AfriPop in 2009 to today, have seen significant advances in mapping technologies and the publication of several gridded population surfaces using different modeling approaches [4]. Two questions for precision public health are how to measure the accuracy of these maps, and how to set standards for accuracy for various purposes. The inherent uncertainty in models and estimates of infectious disease burden is usually recognized while the fundamental uncertainty in the denominator of such disease estimates, the human population data layer, is commonly assumed to be completely accurate.

Here, we measure the accuracy of publicly available, overlapping maps of human population density using a high quality map of one locale as a gold standard [12]. We use several metrics to evaluate these maps, including accuracy profiles and a new goodness-of-fit metric. We evaluate their suitability in the context of malaria control and elimination policy on Bioko Island. The Bioko Island Malaria Elimination Project (BIMEP) has developed highly detailed and constantly updated housing cartography as a basis for distributing interventions, monitoring impact and implementing surveillance [12]. Accompanying this housing database is a recent population census that allocates inhabitants to their households. We also discuss the functional consequences of accuracy by using these surfaces to develop maps of *Plasmodium falciparum* parasite rate (*Pf*PR) with well-documented methods, where the population density surface is both a covariate and the population weight applied to the *Pf*PR surface.

## Methods

### Study area

**Fig 1:**
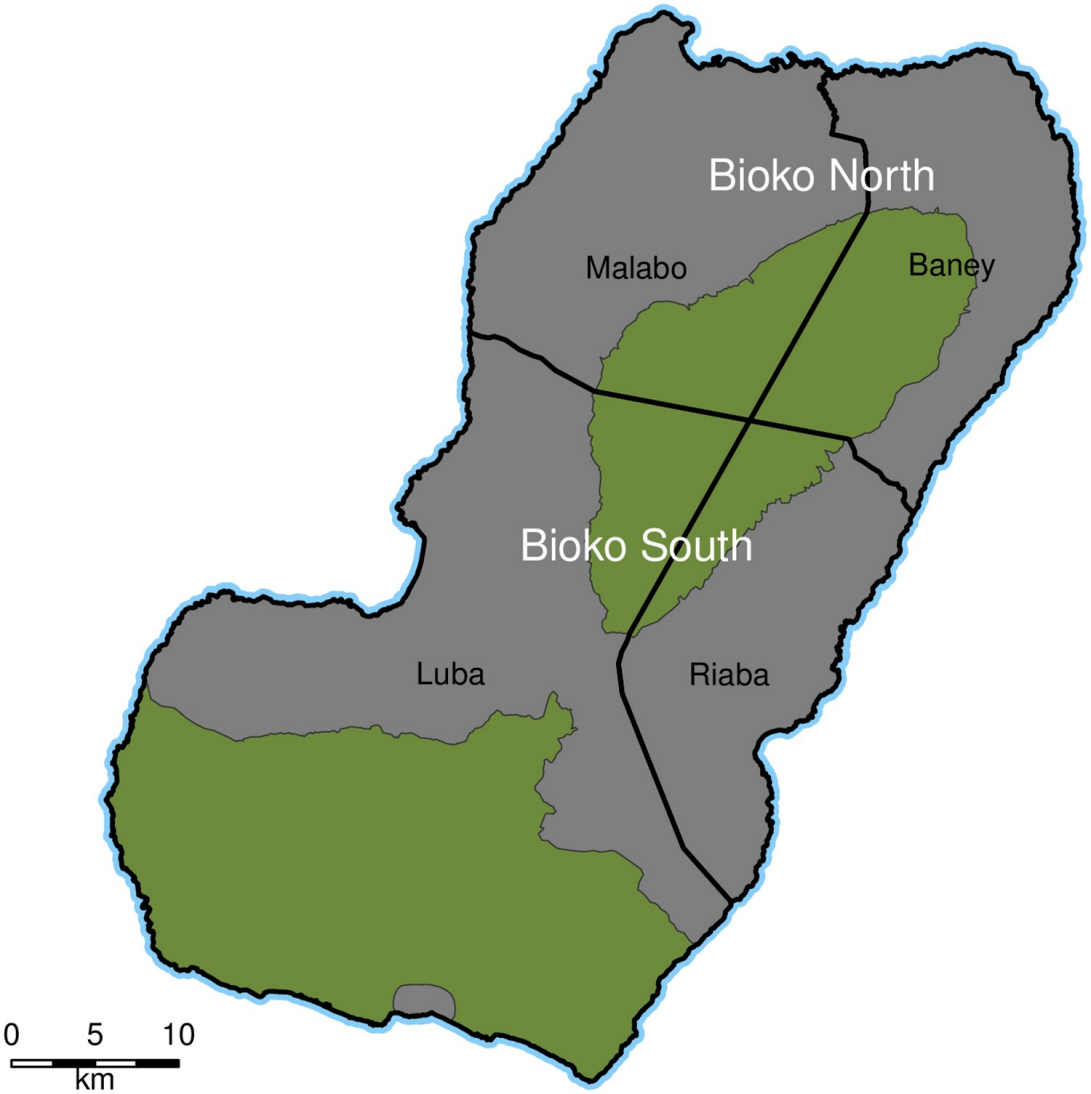
Second and third administrative divisions on Bioko Island. The thick black lines demarcate the four districts. Malabo and Baney make Bioko North and Luba and Riaba, Bioko South. Green areas are uninhabited nature reserves.

Bioko is the largest island of Equatorial Guinea, at 2017 km^2^. It is located approximately 40 km off the coast of Cameroon, in the Bight of Bonny. Malabo, the main urban centre and country capital, has around 85% of the human population of the island. Administratively, Bioko is divided into two provinces (second administrative division) and four districts (third administrative division; Fig 1). A coding system was developed to enumerate all houses on the island [12] based on two virtual grids that cover the island: a 1x1 km grid to define areas and a 100x100 m grid to define sectors within these areas [12].

### Population data

#### BIMEP health census data

This health population census was part of a bed-net mass distribution campaign in 2018 [13]. People present during the campaign were counted and registered to their house mapping code for geographical reference [12]. Each house is GPS-located on the island. The census underestimates the total population count by approximately 12% due to BIMEP census workers only being able to reach approximately 88% of households during the bed-net distribution campaign. The underestimate in the actual population is likely different, however, due to the heterogeneous distribution of intra-household population counts. Despite this discrepancy, the 2018 BIMEP population census represents the most accurate and most up-to-date rendering of population distribution on Bioko Island. This census followed a similar population count in 2015 during the preceding bed-nets distribution campaign, so the data were validated against this previous effort and are used here as the gold standard for analyses.

#### Gridded population data

We selected all population maps that are publicly available, nearly complete across Africa, have data in the last five years, and have any subnational data. These datasets represent two broad categories. Two of these datasets, WorldPop Unconstrained (WP-U) 2018 [14] and Gridded Population of the World (GPW) 2016 [15], begin with areal weighting of census data and include some likelihood of population anywhere it might occur. The WorldPop Constrained 2020 (WP-C) [16] and High Resolution Settlements Layer (HRSL) 2018 [17] datasets use satellite maps of buildings to establish where population might be and then apportion people to those locations. Documentation for LandScan (LS) 2018 [18] describes a combination of these approaches to disaggregate populations across landscapes. These categories result in markedly different assignments of empty space in the population maps, as shown in Table 1.

**Table 1:** Basic summary statistics for all the maps, including the grid size, total population, maximum population density at 1 km^2^, the percentage of pixels that were empty or urban (with >1000 people per km^2^), and the Pareto Number, defined as the percentage *x* that holds a percentage 1 − *x* of the population.

	BIMEP	HRSL	LS	WP-C	WP-U	GPW	BIMEP	HRSL	WP-C	WP-U
Grid Size	923.7 m	923.7 m	923.7 m	923.7 m	923.7 m	923.7 m	30.8 m	30.8 m	92.4 m	92.4 m
Total Population	239056	231210	218044	365265	362962	365766	239056	231210	365265	362962
Max. Pop. Dens.	19909	6304	34344	6842	2356	565	22212	7525	7139	2547
% Empty	89.16	86.39	56.29	83.13	0.041	0.2876	99.13	98.92	96.63	0.11
% Urban	1.902	3.06	2.075	4.436	3.826	0	0.8733	1.076	3.367	3.951
Pareto Number	3.763	5.749	5.275	7.794	22.05	23.79	3.904	5.643	7.812	22.29

The next distinguishing feature seen in Table 1 is grid size. Both LS and GPW use the same coarser grid, with grid cells 923.7 m a side at Bioko Island’s latitude. Both WP maps have grid cells 92.4 m a side, and HRSL grid cells are 30.8 m a side.

While most of these maps estimate population for the year 2018, to match the Bioko Island survey data, the WP-C map is available only for the year 2020. We include it in this analysis by applying the U.N. estimate for Equatorial Guinea population growth, 3.66% per year [19], to rescale the population per pixel of the WP-C map.

The size of grid cells particularly affects the distribution of empty cells [20], so we aggregated the HRSL 30-fold and LS 10-fold, in order to construct nearly 1 km grids. We refer to these as 1 km maps. Rescaling of WP-C and aggregation of finer maps were the only preprocessing on the gridded maps.

When there is direct comparison between the house-level BIMEP data and a gridded dataset, we aggregated the BIMEP data to the same grid as the dataset. This way, each map is compared without added interpolation from alignment to a common grid. When algorithms called for BIMEP data to be gridded, we used the finest grid, that of the HRSL.

### Goodness of fit ratios

Previous work used root mean square error in order to mimic a confidence interval estimate [3]. We propose a new measure to assess accuracy of a map. This measure looks at how much a map improves upon a null-hypothesis map, one that assigns each pixel the average population density. The goodness-of-fit ratio (GOFR) is the ratio of the mean sum of squared errors to the variance. The mean sum of squared errors is the sum of the bias and the variance, so a null-hypothesis map will have a GOFR of one, a perfect map will have a GOFR of zero, and values larger than one indicate the fit is worse than the null-hypothesis map.

Let *H*(*x*_*i*_) denote the true population density at pixel *x*_*i*_, and let the observed map value be denoted $\hat{H}\left(x_{i}\right)$. For any subset of map pixels, the GOFR is the ratio of mean squared error per pixel to variance of the expected. Using *n* for the number of pixels in a region, the GOFR for that region is

$$\begin{array}{c} G\left(\hat{H}\right)=\frac{\sum_{i}{\left(\hat{H}\left(x_{i}\right)-H\left(x_{i}\right)\right)}^{2}/n}{\text{Var}(H)}. \end{array}$$ (1)

We can also compute a normalized by district GOFR to compare relative population densities, to remove any effect of having different total population sizes. Let *h*(*x*) = *H*(*x*)/∑_*y*_ *H*(*y*). The normalized version is

$$\begin{array}{c} g\left(\hat{h}\right)=\frac{\sum_{i}{\left(\hat{h}\left(x_{i}\right)-h\left(x_{i}\right)\right)}^{2}/n}{\text{Var}(h)}. \end{array}$$ (2)

If *G* = 0 then the candidate map is perfect over the region. If *G* < 1, then the map improves the goodness of fit over the constant value, and if *G* ≥ 1, the goodness of fit is equal or worse than just using the average population density.

### Urban fraction

Countries measure urban fraction in ways that are germane to their needs for planning and assessment, so the measure can include observations of human movement patterns and availability of resources [21–23]. Because we are looking only at the maps, the urban fraction here is the percentage of pixels for which the density within a km^2^ was greater than a thousand people [24]. It may be that Equatorial Guinea uses a cutoff of 1500 people per km^2^ [25], but the relative information in these maps is the same for either choice of cutoff.

### Pareto number

The Pareto number is a single value that characterizes the tendency of population to aggregate. A smaller value indicates more aggregation [26]. For instance, if 95% of the population is in 5% of the pixels, the Pareto number would be 5. We find the Pareto number by sorting pixels in increasing population size. The Pareto number is the index, normalized to 100, of the pixel for which the fraction of pixels that are larger equals the fraction of total population in pixels that are smaller.

### Accuracy profiles

We used binary classification statistics to construct an accuracy profile for the 1 km maps. We did not do so for the 100 m maps as LS did not have a 100 m raster layer (Table 2). Binarization of population quantity numbers was done to allow comparison of accuracy statistics across multiple population surfaces. For a threshold population density, *τ*, each pixel in a map is classified as being either above or below the threshold. Using BIMEP as the gold standard, we assessed the accuracy of the other maps against it and each other. We computed: true positives (TP), the proportion above the threshold in both maps; true negatives (TN), the proportion below the threshold in both maps; false negatives (FN), the proportion above in BIMEP but below in the other map; and false positives (FP), the proportion below the threshold in BIMEP but above in the other map. We define accuracy as the proportion correct (i.e. (*TP* + *TN*)/(*TP* + *TN* + *FP* + *FN*)); recall or sensitivity as the proportion above a threshold in the gold standard that were correctly assigned: *TP*/(*TP* + *FN*); and precision, or positive predictive value, as the proportion above the threshold in the alternative map that were correctly assigned: *TP*/(*TP*+*FP*). Each threshold value on population density, *τ*, gives different measures of accuracy, recall, and precision. We also computed accuracy metrics for classification of the landscape into population density categories, using breakpoints at 1, 50, 250, and 1,000 people per km^2^, with 1,000 and up classified as urban areas.

**Table 2:** Let a threshold, *τ*, define a categorization of population density. In a gold standard map, *G*, a pixel is in the category if it is above the threshold: *x* ∈ *G*_*τ*_ if and only if *x* > *τ*. Otherwise, *x* ∉ *G*_*τ*_. Similarly, the categorization is applied to a candidate map, *M*. Pixels are classified as true positives (TP), true negatives (TN), false negatives (FN), and false positives (FP) as described in the table. Accuracy profiles are plotted in Fig 6.

*x* ≥ *τ*	*x* ∈ *M*_*τ*_	*x* ∉ *M*_*τ*_
*x* ∈ *G*_*τ*_	TP	FN
*x* ∉ *G*_*τ*_	FP	TN

### *Pf*PR mapping

We estimated the prevalence of malaria parasites, a *Pf*PR surface, for Bioko Island using the same set of covariates, replacing only the population surfaces one at a time. Data and methods have been described elsewhere [9, 10]. The population surfaces were then used to construct density values to assign as population weights for use in the calculation of *Pf*PR. The response data corresponded to *Pf*PR at household-level spanning the period 2015–2018. We ran this exercise for each of the 1x1 km population grids since environmental covariates were not available for Bioko at finer spatial resolution. We also estimated relative populations at risk using each population surface and expressed them as cumulative distribution and probability density functions.

## Results

### Population distribution

**Fig 2:**
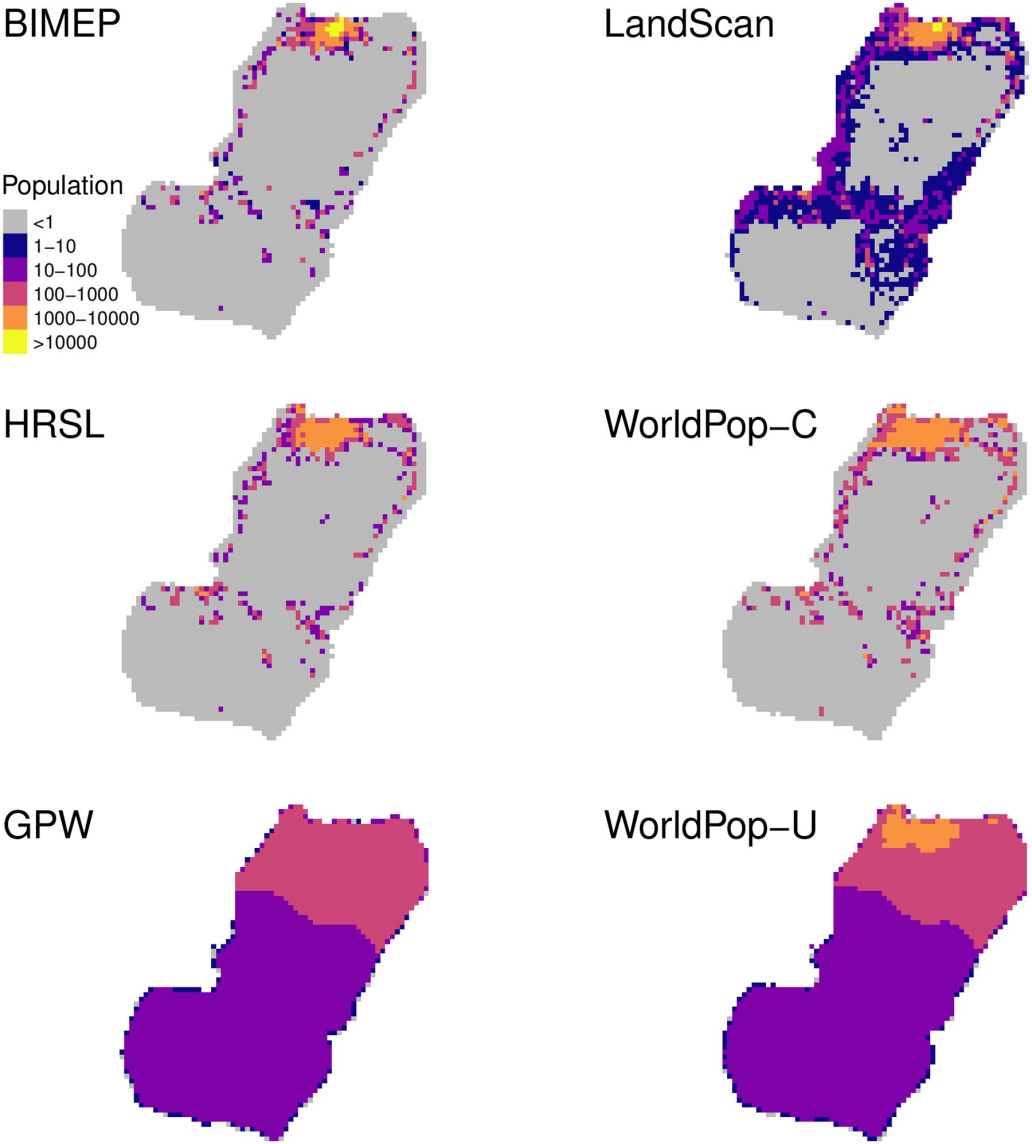
Bioko population rendered at 1x1 km resolution. Population colors range from greater than 10,000 persons per km^2^ to less than 10 per km^2^. Grey pixels represent uninhabited areas (population = 0).

**Fig 3:**
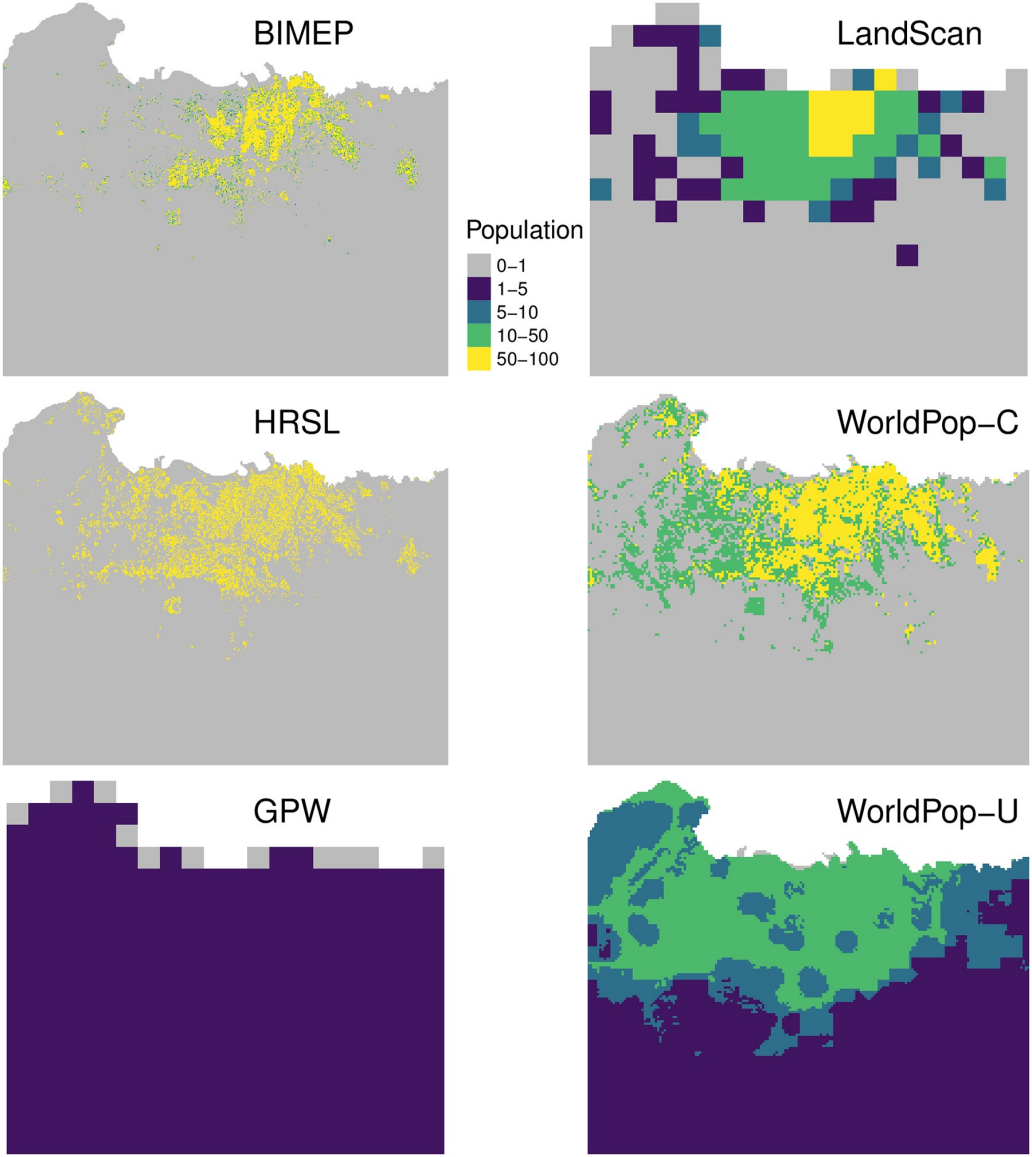
Gridded maps at their native resolution, zoomed into the Malabo area as visualization of a highly populated area for comparison. Grey pixels represent uninhabited areas.

Much of the habitable land area on Bioko Island is sparsely inhabited and the bulk of the population is concentrated in the North, within and nearby Malabo. Urban Malabo is the yellow pixels in the BIMEP map of Fig 2. The rest of the population is distributed in pockets, mostly rural, along the East and West coasts. There are two large, uninhabited nature reserves in the North and South of the island (Fig 1). Figs 2 and 3 illustrate the population distribution according to each of the four surfaces at 1x1 km, 100x100 m, and 30x30 m, respectively. In the BIMEP surface (Fig 2), the population is highly concentrated around urban Malabo, with areas housing as many as 22,212 people per km^2^. Fig 3 illustrates a highly heterogeneous human population distribution in the center of Malabo at 100x100 m pixels. Given the small size of Bioko and aggregation of its population, it is a success for the metrics to present a consistent picture of map performance.

The 1 km LS population surface, while correctly identifying the general shape of settlement around the Malabo area, fails to predict the extreme abundance of zero population pixels in the rest of the island. Within Malabo, the LS model severely over-predicts extreme population aggregation, estimating 34.6% of the total population is concentrated amongst two km^2^ with population density as high as 34,344 people/km^2^ (Fig 2).

The HRSL surface renders a more accurate population distribution overall, particularly in rural areas (Fig 2). It fails to provide an accurate picture of urban Malabo, however, where the population appears more evenly distributed than the gold standard, with a maximum population density of 6,304 per km^2^. This pattern is also manifest in the 100x100 m HRSL surface, representative of an overly uniform population distribution across Malabo (Fig 3).

The population according to WP-U distinguishes average density between Bioko North and Bioko South but diverges little from mean values, including within the nature reserves (Fig 2). The 100x100 m WP-U surface does show increased densities in the larger Malabo municipality, but it is overly uniform and does not quantify empty locations with zero population or dense urban population density (Fig 3). WP-C in contrast has a more realistic and accurate population allocation and dispensation around the island (Fig 2). The 30x30 m WP-C surface matches much closer to the distribution of population shown by BIMEP around Malabo but much like the 1x1 km surface has some over distribution into empty locations (Fig 3).

The 1 km GPW population surface had the least realistic and accurate distribution. It was able to capture only a general north/south population difference and failed to categorize empty space or urban areas (Fig 3).

### Per-pixel scatter plot

**Fig 4:**
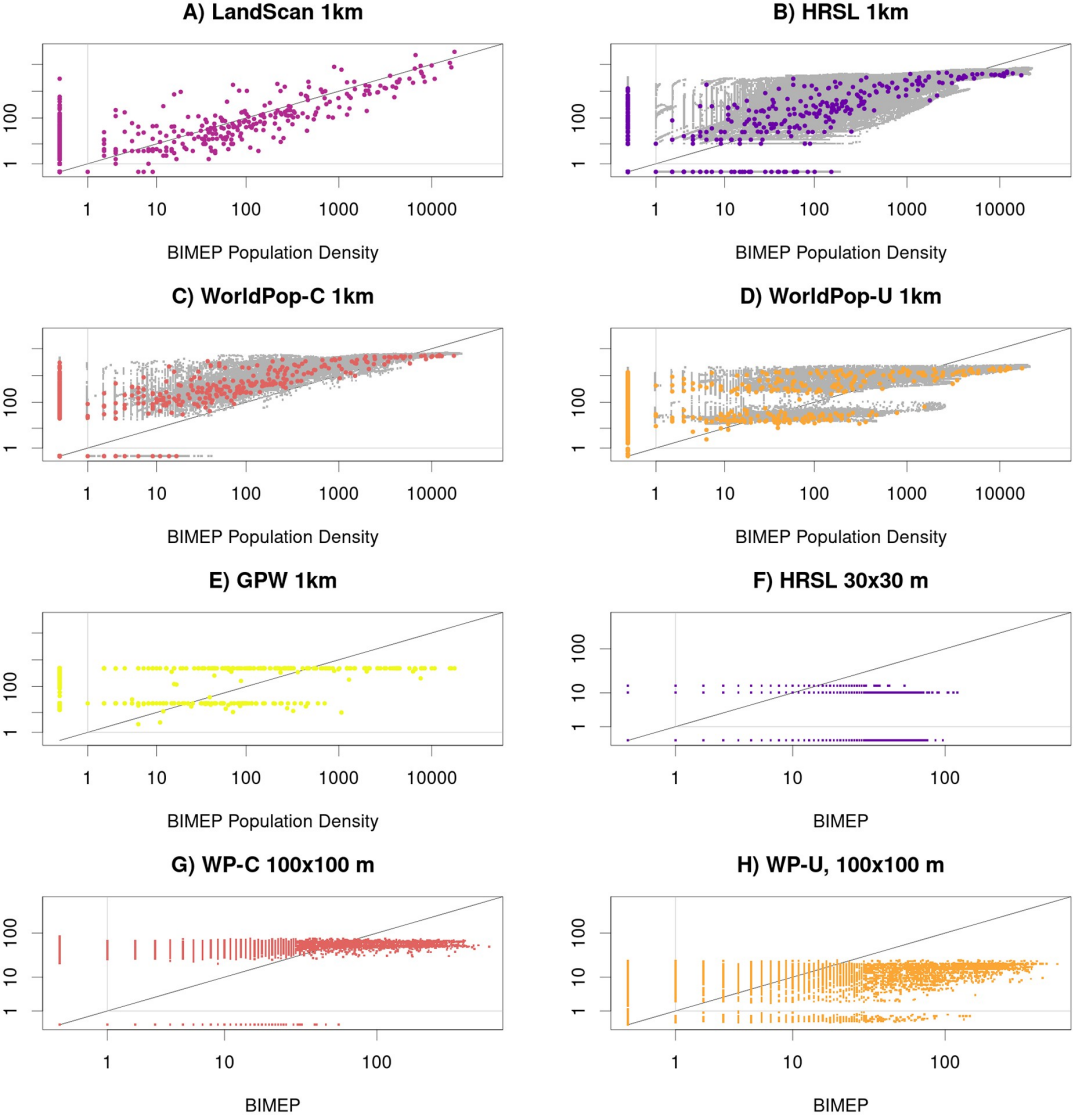
These are scatter plots of the population count in each pixel as a function of the population count in each pixel according to BIMEP. The solid black line indicates one-to-one agreement. The HRSL, WP-C, and WP-U maps are also shown aggregated to the 1 km scale. These maps have a background of grey points, which are the 1 km density of each pixel of these three maps. The *R*^2^ values of the 1 km maps are 51% for LS, 70% for HRSL, 62% for WP-C, -6% for WP-U, and -11% for GPW. At finer scales, where registration can be more difficult, the *R*^2^ values are -4% for HRSL, 48% for WP-C, and -2% for WP-U.

The biases and exactness of the algorithms used to generate the maps can be observed in the scatter plots (Fig 4). The LS scatter plot shows a roughly linear distribution across population densities which indicates a natural density per pixel vs categorization of population density of some of the other surfaces. The HRSL layer, which had a similarly linear distribution, underestimated at the highest distributions and mischaracterized some pixels as empty at the lower population level compared to the BIMEP layer. WP-C does a better job of evenly distributing density but overestimates densities across the total range and, like HRSL, lacks low range densities between 1–10 persons per pixel. GPW like WP-U at 1x1 km displayed the two horizontal lines where it classified all of the range of BIMEP population densities into two levels of density per pixel. The plots for the 30x30 m maps for HRSL and 100x100 m maps for WP-U and WP-C show distinct horizontal striping patterns (Fig 4). The stripes indicate that for a large range of actual (BIMEP) population densities, both WP surfaces and, to a lesser extent, HRSL predicted constant density; this is a visual indication of model inability to fully characterize spatial variation in population density. The HRSL estimated map is produced by an algorithm which, in this case, identifies a maximum of 20 households in each pixel, and allocates the total population evenly among households, producing here an integer multiple of 10.144 individuals to each household. In WP-U, each grid square is assigned to one of seven distinct population density values. These patterns are obscured in population density estimates or in aggregating data up to 1x1 km grid cells. The adjusted *R*^2^ values for the per-km maps are higher than for their respective estimated population density, which are higher than for the population size as well.

### Cumulative distribution by area

**Fig 5:**
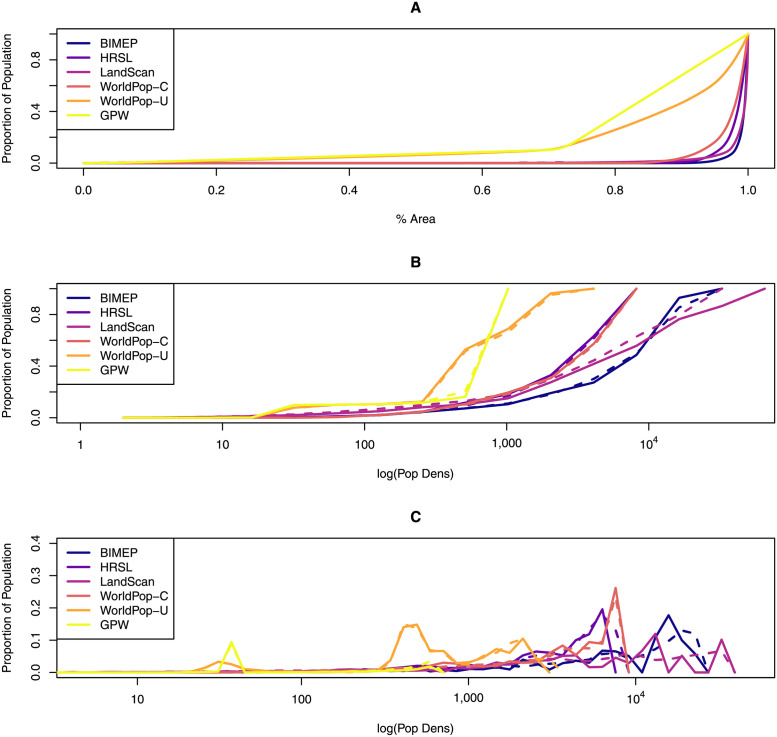
A comparison of the distributions by land area and population density. Solid lines are 1x1 km maps, and dashed lines are 100x100 m maps. A) To show how the population is distributed, we plotted the empirical cumulative distribution functions (eCDFs) of population density by land area; B) To show how the population is aggregated, we plotted the eCDFs by log population density. C) The population density binned by powers of 1.2.

Some of the same patterns are evident in the empirical cumulative distribution functions (eCDFs) and smoothed density plots showing population density and its distribution by land area (Fig 5), which highlight the large fraction of empty space in most of the maps (Fig 5A). In the BIMEP maps, the fraction of empty pixels was 99% for the 30 m map and 89% for the 1 km map; both the HRSL and LS were similar (Fig 5B). WP-U, by way of contrast, reported a positive population density for almost every pixel. We have also plotted the eCDFs of population density vs. the proportion of the human population living at that density, and also the empirical probability distribution functions (Fig 5B and 5C), which highlight the inaccuracy of GPW and WP-U at population densities greater than 100. It also shows the similar performance of the HRSL and WP-C layers. These maps highlight important differences, such as maximum population density: 34,344 per km^2^ for the LS map, 19,909 for the BIMEP map, 7,525 for the HRSL map, 565 for GPW, and 7,139 and 2,547 for WP-C and WP-U respectively. (Table 1).

### Goodness of fit ratios

We applied the GOFR to all population maps at the 1 km resolution for comparison. The HRSL was an improvement over the average population density for all of Bioko Island and for each one of the third administrative levels (Table 3). The LS map was an improvement for all but the Luba district. The WP-U and GPW maps were remarkably close to an average population density map overall. The GOFR values for Luba and Riaba were larger, in general. A small registration error in a map could result in a large GOFR because the individual districts’ populations are small (5500 in Luba, 2300 in Riaba) and highly aggregated. WP-U and GPW are approximately as informative as the null-hypothesis map.

**Table 3:** This compares the goodness-of-fit ratio across the three maps, aggregating HRSL and both WP surfaces to 1 km resolution to match LS and GPW. Normalization discounts the effect of uniform changes in population size, which provides a better comparison between high-and-low population districts.

	GOFR					Normalized GOFR				
	LS	HRSL	WP-C	WP-U	GPW	LS	HRSL	WP-C	WP-U	GPW
Bioko Island	0.472	0.414	0.433	0.758	0.980	0.545	0.406	0.491	0.814	0.971
Baney	0.374	0.157	0.427	0.917	1.772	0.136	0.137	0.343	0.908	0.997
Luba	5.484	2.458	1.707	1.049	1.223	1.658	0.164	0.195	0.987	1.000
Malabo	0.487	0.452	0.446	0.775	0.987	0.570	0.424	0.488	0.782	0.986
Riaba	0.824	1.337	6.183	1.151	1.207	0.458	0.384	0.287	0.978	1.003

### Accuracy profiles

**Fig 6:**
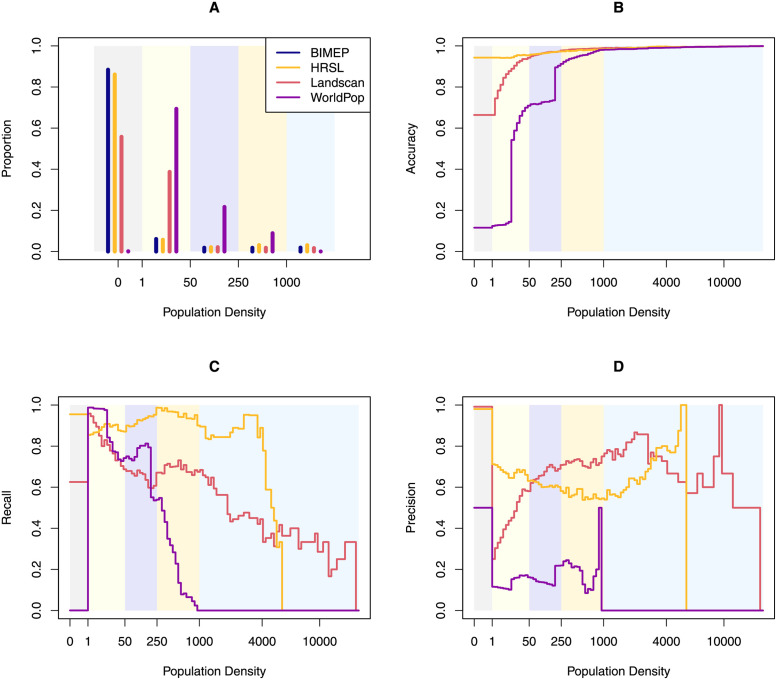
A) The proportion of the population in density categories defined by breakpoints of 1, 50, 250, and 1,000 people. B) The accuracy profile; C) The recall profile; D) The precision profile.

The accuracy profile shows measures of accuracy, recall, and precision as binary classification statistics for a mesh on population density for values spanning the range of the gold standard (Fig 6). Of these metrics, the maximum population is most sensitive to grid placement, especially for the coarsest grid choice. The BIMEP and HRSL maps were the most similar across all three binary metrics. LS and WP-C had a much higher fraction of pixels in the lowest population category (Fig 6A). Overall, the HRSL tended to be the most accurate and with the best recall for population densities up to 5,000 per km^2^, which was the upper limit of population densities in that map (Fig 6B and 6C). The precision of the LS map was highest from 250 people per km^2^ up to around 2,000 people per km^2^ (Fig 6D).

The HRSL map identified 98% of the empty pixels (precision) and 93.5% of the urban pixels correctly (recall), while LS identified 74.5% of the urban pixels correctly. Notably, the accuracy metrics are dominated by true negatives, since there are so many empty cells (Table 4). WP-C nearly matched the performance of HRSL in accuracy, recall, and precision, except in the 1–50 range of values. Unlike the other WP layer, WP-U had significantly worse accuracy profiles except that its precision matched BIMEP’s empty pixels 100%. GPW overwhelmingly had the worst recall and precision of all layers.

**Table 4:** The accuracy, recall, and precision for the population classifications shown in the header and illustrated in Fig 6.

*H*	(0, 1)	[1, 50)	[50, 250)	[250, 1000)	[1000, ∞)
Accuracy					
HRSL	0.9440	0.9480	0.9600	0.9670	0.986
LS	0.6720	0.6740	0.9530	0.9750	0.989
WP-C	0.9340	0.9470	0.9170	0.9420	0.974
WP-U	0.1150	0.3260	0.9280	0.7550	0.976
GPW	0.1150	0.3160	0.9570	0.7110	0.980
Recall					
HRSL	0.9530	0.3640	0.4000	0.4320	0.935
LS	0.6320	0.7500	0.5380	0.2270	0.745
WP-C	0.9310	0.0818	0.1270	0.2780	0.978
WP-U	0.0028	0.5270	0.0380	0.3610	0.870
GPW	0.0000	0.6200	0.0125	0.7270	0.000
Precision					
HRSL	0.9840	0.3640	0.3950	0.2130	0.581
LS	0.9970	0.0869	0.3770	0.3030	0.729
WP-C	0.9950	0.2570	0.0714	0.0800	0.421
WP-U	1.0000	0.0356	0.0300	0.0223	0.430
GPW	0.0000	0.0358	0.0500	0.0469	0.000

LS had the highest precision (i.e. positive predictive value) for “empty”: if a pixel was reported empty in LS, it was empty in the BIMEP map 99.7% of the time. The HRSL was a close second at 98% precision. The precision values for urban classification were lower: LS was the highest at 72.9%, while HRSL had 58.1% (Table 4).

### Malaria mapping

**Fig 7:**
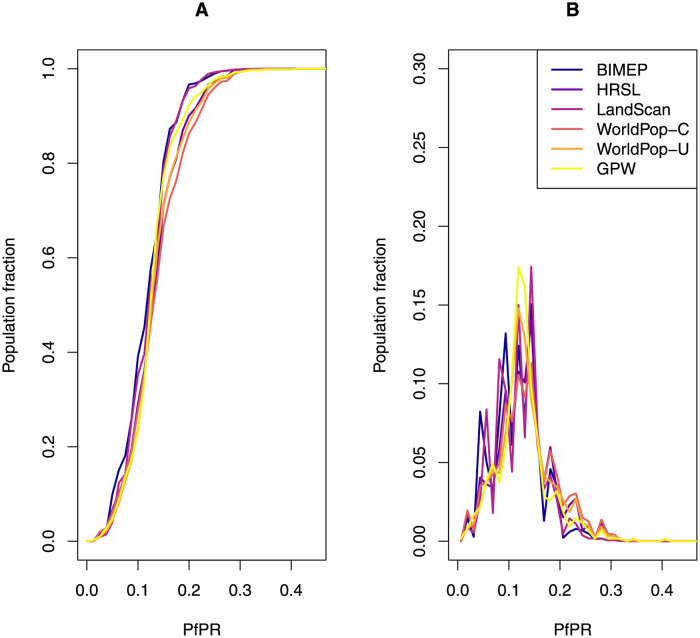
Fraction of the population according to *Pf*PR, expressed as cumulative distribution (A) and probability density functions (B).

In our analysis, human population density was only weakly correlated with *Pf*PR on Bioko Island, so the resulting *Pf*PR maps were virtually indistinguishable. The main difference was how the distribution of people affected calculation of average *Pf*PR: 11.3% for the BIMEP map, 12.3% for the HRSL, 13.4% for WP-U, and 10.6% for LS (Fig 7A). Calculating the fraction of the population at greatest risk, with *Pf*PR above 20%, was sensitive to the map’s ability to identify urban areas, leading to 2.5% in BIMEP, 2.6% in LS, 6.2% in the HRSL, and 12.4% in WP-U (Fig 7B).

## Discussion

If you want to compare maps qualitatively, then an image, such as Fig 2, tells the whole story. It does not, however, give you the tools you need to interpret how bias in maps affects their use as inputs to further calculations. The metrics in this paper offer multiple ways to understand maps better. The per-pixel scatter plot shows tell-tale quantization of pixels. The eCDF shows rural and urban bias. The GOFR offers a new summary statistics, and accuracy profiles expand our estimate of correct and incorrect pixels to categories of accuracy, recall, and precision. Together these provide insight into optimal use and limitations of these datasets.

There are limitations to this work as a case study because the study area is small enough to have only one urban cluster and because the maps it compares will be superseded by updated maps. More important may be limitations on the metrics. Both GOFR and accuracy profiles rely on having a gold standard map, while the other metrics are helpful without it. While the metrics do identify biases in maps, they point to a need to better understand how these lead to biases in derived outputs.

Overall, WP-C and HRSL datasets both characterized the population of Bioko Island, as gathered in the BIMEP census data, the most correctly. Metrics like the Pareto number, recall fraction, and GOFR show that the HRSL is better at representing sub-urban densities. We say that LS has the best characterization of urban/rural ratios because its precision and recall scores are best for high population densities. Lastly, WP-U and GPW have similarly low *R*^2^ values and high GOFR values for this area.

### WorldPop

The WorldPop results for both WP-C and WP-U consistently under-reported population levels in urban areas (Fig 6). In Table 1, WP-U is close to lowest max population density. This is likely due to WP-U maxing out population at 2547 people per km^2^, which is a low estimation for a primarily urban population like we see in Bioko Island where a majority of the population lives in Malabo. The average population density in Malabo is nearly 14,000 per km^2^ which results in a significant undercount. On the opposite end of the population spectrum, WP-U severely overestimates population densities everywhere else, with the lowest number of empty pixels (Fig 5). This has been seen before in other African countries and may be a product of the random-forest model approach running on the entire map [14]. WP-U, like GPW, shows population living in the large nature reserves in the North and South of the island where there are no settlements (Fig 1). Both underestimation of high population areas and overestimation of low population areas may be the result of the WP-U population model being anchored by official census data at the second administrative division [14]. Heavy reliance on the mean of census data could be problematic for countries where census data is of poor quality because these countries report data only at higher administrative levels.

In contrast, WP-C had much better performance, with similar overall empty space and max population density to HRSL. WP-C was able to capture the population distribution pattern more similar to BIMEP across the island (Fig 2) and in the urban areas in and around Malabo (Fig 3). The scatter plots revealed a much more continuous distribution of values similar to LS at 1x1 km and although there was some binning of data, an even better distribution than HRSL at 100x100 m (Fig 4). The eCDFs plots (Fig 5) also show WP-C to be a much better population density match to BIMEP. The GOFR of WP-C was also consistently greater than 90% across all population density classifications. This improvement in population characterization compared WP-U is a result of using satellite imagery as a “mask” to rule out uninhabited areas and only run the random-forest model within those 100x100 m grids identified with buildings/structures. In doing this, WP-C improves both identification of zero population spaces and urban population resolution [16].

### LandScan

The LandScan global gridded population surface was the best at characterizing high-density urban population distribution but was the only of the three to overestimate population density compared to BIMEP (Table 1). The LS 1 km grid layer had the highest accuracy and precision in Malabo and high density (greater than 1000 people per pixel) areas (Table 4). If the two highest density pixels were removed, the LS layer’s GOFR scores improved significantly. Similarly, LS was only able to categorize 56% of the empty pixels, which results in high GOFR scores for rural districts of Bioko, but when normalization for each district by population was applied, the GOFR score improved in Riaba and Baney, which are largely rural districts. Since LS is not currently available at the 100 m resolution, we do not know if it would improve performance at categorizing empty space as we observed in the HRSL, WP-C and WP-U. The distribution of the mapped population density compared to the BIMEP population density was the most linear relationship and did not show the binning of population which is seen in the other gridded surfaces. The misallocated pixels on the y-axis were also distributed from both high and low population distributions without an obvious skew. LS outperforms WP-U at all population densities (Fig 6) and outperforms HRSL at high population densities in precision and recall. The construction of LS datasets incorporates population area weights based on administrative areas as well as land use classification down to the 1 km pixels. In places such as Bioko Island, in the absence of reliable official local geo-referencing, this could result in the LS surface distributing the entire district population according to only the population likelihood locations and not from imagery and census data [27]. Fuzzy spatial characterization of land use assignments, which is common in Africa at the fine scale gridded resolutions we are looking at, could result in an output reflective of a residential only population distribution rather than an ambient population distribution with mixed use or areas where people are not permitted to live.

### High-resolution settlement layer

The HRSL had the best performance overall. The HRSL still underestimated the maximum population density compared to the BIMEP gridded census data by around 66% in both the 1 km and 100 m pixel grid surfaces (Table 1). Both BIMEP and HRSL were very close in percent urban, percent empty, and overall island population total. It also provides the highest population accuracy, recall, and precision across the majority of the population density categories, especially for all the empty pixels (Table 4). The HRSL had the lowest GOFR ratio after normalization across all four districts and visually was the most like BIMEP (Fig 2). The HRSL scatter plot at 1 km had the best *R*^2^ value of any surface at 70%. The HRSL scatter plot at 30 m showed population count binning lines like we observed in WP-C, and a negative *R*^2^ value for this scatter plot suggests that any individual 30 m pixel is not as informative as a mean value. We found the HRSL did not match the BIMEP distribution only at population densities greater than 1,000 (Fig 5B), and this was true for both 1 km and 30 m maps. The HRSL defines an urban area as 10,000 people or greater, so it is possible that the HRSL is unable to assign greater than 10,000 people to a single gridded pixel. The proportion of population by density for HRSL was closest to BIMEP at each density category (Fig 5), but while HRSL had the greatest accuracy across all population densities, there was a drop off in recall and precision at the same point before 10,000 people per pixel. The HRSL settlement layer most closely matched the BIMEP surface, which indicated that for Bioko Island it was the most correct human population map we examined. The challenges the HRSL population surface had characterizing high density populations bear further examination but may be due to the structural image mining approach the model is based on. Even with this consideration, our findings show that approaches to human population maps that rely more on remote sensing and image processing are better able to discern where there are not any people, which, in combination with official census data, produces an informative map.

### Gridded population of the world

The GPW population surface when projected onto a Bioko map showed how the layer displayed even population levels across the whole island (Fig 2E). GPW allocated population only by high/low population levels, contrasting between the more populous north and less populated south of the island. The two population levels were between 10–100 and 100–1000 (Fig 4F), which resulted in zero empty pixels and zero high (above 1000) population pixels (Table 4). GPW was unable to identify urban (dense) population areas at all as its population density maxed out at 565, the lowest of all the datasets we looked at. In both the empirical cumulative distribution functions and goodness-of-fit ratios it had the worst match to BIMEP’s distribution. GPW’s GOFR did not improve much even after normalization. Based on our results, GPW seems to be suitable only for regional-level population estimates and does not seem adequate for use even in country-level analyses. This is not unexpected as it is meant to be a “world” population dataset and illustrates the trade-offs of creating an accurate macro vs. micro level population surface. Although it had the worst performance of the datasets compared, this does not attest that the dataset is bad, only that it does not match the BIMEP census results and is likely not useful for creating sub-1km^2^ population estimates.

### Malaria burden estimation

The disparity in the results for *Pf*PR values computed from the population density surfaces was consistent with previous studies examining the effect of population layers on malaria modeling estimates [28]. The *Pf*PR estimates were very similar between WP, LS, and the HRSL but there was a noticeable difference between the estimated population fraction infected between layers when the *Pf*PR was between 10% and 30% (Fig 7A). LS tended to overestimate the population fraction at *Pf*PR around 10% compared to BIMEP but at 20% and greater had similar cumulative and probability density curves. WP and HRSL had a consistently lower population fraction at *Pf*PR levels than BIMEP, however the HRSL had a smaller difference in population fraction in both the cumulative distribution and probability density functions (Fig 7B). Although the HRSL had a more similar dispersal overall compared with BIMEP, the LS surface had a more accurate distribution of population in central Malabo. This improved urban quantification could better fit population curves with BIMEP population distribution at *Pf*PR cutoffs above 15%. While the population fractions were only several percentage points different between surfaces, on an island wide scale this represents several thousand potential malaria cases. Additionally, the range where we see the difference in results is around the mesoendemic to hypoendemic transmission threshold, which is where Bioko Island’s parasite prevalence rate is currently estimated [29]. Our results suggest the disparity in models is most apparent at these transition points between endemicity levels, which demonstrates the importance of using the most correct human population maps for modeling and estimating malaria.

## Conclusion

Having gold standard data at any spatial scale is useful as a benchmark for gridded human population density surfaces. This data provides the scale with which to evaluate the goodness-of-fit ratio GOFR. It provides true values for accuracy profiles, whose recall metric was a strong discriminator of these maps. Even in the absence of gold standard data, plots of empirical cumulative distribution functions (eCDFs) for some representative area would give a detailed understanding of biases among available population maps. These metrics are demonstrated in the repository of code for this article, provided for Guidelines for Accurate and Transparent Health Estimates Reporting (GATHER) compliance [30, 31].

All of these maps are, themselves, models, which carry traces of their chosen source data and algorithms. This quantitative analysis highlighted strengths and limitations of those models, from caps on population per pixel to remarkably good identification of rural house locations. The human population datasets that included satellite imagery to identify structures: HRSL, LS, and WP-C, were a clear improvement over WP-U and GPW that did not. If, in the future, the HRSL could improve its estimation of the relative size of each household, it could provide a single source for both urban and rural populations at 30 m resolution. Meanwhile, some combination of LS and HRSL would be the closest match in BIMEP.

## Supporting information

S1 Appendix (PDF)

S1 File (DOCX)
