# Supplementary material for: Measuring the accuracy of gridded human population density surfaces: A case study in Bioko Island, Equatorial Guinea
Source: PLoS One. 2021 Sep 1;16(9):e0248646. doi: 10.1371/journal.pone.0248646 (PMC8409626; doi:10.1371/journal.pone.0248646)
Supplement: S1 Appendix — (PDF) [file pone.0248646.s001.pdf]

Measuring the accuracy of gridded human population density surfaces: a case study in Bioko Island, Equatorial Guinea: Supplement A

Brendan F Fries, Carlos A Guerra, et al

Contents

1 Overview **1**
1.1 Data from different years . . . 2
1.2 Conventions . . . 2

2 Download Datasets **2**

3 Align to Grids from BIMEP GPS Data **3**
3.1 HRSL . . . 3
3.2 LandScan . . . 5
3.3 WorldPop Unconstrained . . . 6
3.4 WorlPop Constrained . . . 9
3.5 Gridded Population of the World . . . 10

From git@github.com:dd-harp/population_comparison_bioko.git on Sat May 22 10:48:56 2021, generated by adolgert.

1 Overview

There are three parts to this supplement. This first section aligns and formats maps. The next section, appended below, performs calculations and makes graphs. Finally, there is a GATHER statement, which is one of the protocols for open sharing of data and computation.

This notebook retrieves population maps for Bioko Island, in Equatorial Guinea, and converts it into gridded data about population density. This notebook should retrieve and clean data so that we can pass that data to another notebook to do statistics.

This notebook performs these tasks:

1. Read public gridded population datasets and read the Bioko Island household survey data.
2. For each fine-grained grid dataset, aggregate it to a nearly-1 km version. For each coarse-grained grid dataset, disaggregate it to a 100 m version.
3. For each grid dataset, use the point data from the Bioko Island household survey to create a gold-standard equivalent on the same grid.

The BIMEP GPS data is not publicly available. You would direct a request to the BIMEP Mapping Team, which produced this data set with the support of the National Malaria Control Program and the Ministry of Health and Social Welfare of Equatorial Guinea, as well as Marathon Oil, Noble Energy, AMPCO (Atlantic Methanol Production Company) and the Ministry of Mines and Energy of Equatorial Guinea.

The code for this work is too large for the notebook, so it is in the Github repository listed at the top of the document. All specialized function calls in this document refer to that repository.

1.1 Data from different years

Some of the datasets come from different years. Many of the metrics are insensitive to total population because they look at distribution functions, but there are places where adjustment for years makes sense. We can rescale populations according to the growth rate for Bioko. A commonly-used adjustment is to multiply values by $\exp(rt)$, where r is the yearly growth rate. For Equatorial Guinea, that's 3.7% in 2017-2018, according to UN sources. This wouldn't affect the structure of zeroes, though.

UN World Population Prospects 2019. Equatorial Guinea, average annual rate of population change (percentage), from https://population.un.org/wpp/DataQuery/:

```
df = data.frame(
  five_year = c("2000-2005", "2005-2010", "2010-2015", "2015-2020"),
  annual_percent_change = c(4.24, 4.61, 4.28, 3.66)
)
```

If we take the yearly growth as 1.0366, then $e^rt = 1.0366$, so $r = log(1.0366)/t$.

```
growth_rate_per_year = log(1.0366) / 1 # year
```

If WorldPop has a size of 362,962 people in 2020, what would that mean in 2018?

```
c(362962 * exp(-2 * growth_rate_per_year), exp(-2 * growth_rate_per_year))
#> [1] 3.377837e+05 9.306312e-01
```

So that's 337,784, a decrease to 93% of what it was, and BIMEP is at 239,056.

1.2 Conventions

Incoming datasets sometimes mark water as a missing value and sometimes set the population over water to zero. We will use a template for the boundary of land and mark pixels in the water as missing, or NA in R.

All work will use pixels in latitude and longitude (lat-long). This is also known as an unprojected space. All of the gridded datasets are rectilinear in lat-long.

2 Download Datasets

Specify a directory into which to put the data. The `inst/extdata` subdirectory is a popular place.

```
data_dir <- params$data_directory
if (!dir.exists(data_dir)) {
  dir.create(data_dir, recursive = TRUE)
}
```

The data will be stored in /home/adolgert/dev/popbioko/inst/extdata.

The Bioko shapefile has one geometry, a polygon outline of Bioko. We will use it to project population from rasters. This Shapefile comes from BIMEP, but you could use a cropped GADM shapefile.

```
latitude_longitude_projection <- "+proj=longlat +ellps=WGS84 +datum=WGS84 +no_defs"
bioko_sf <- sf::st_read(fs::path(data_dir, "source", "bioko.shp"))
#> Reading layer `bioko' from data source `/home/adolgert/dev/popbioko/inst/extdata/source/
#> Simple feature collection with 1 feature and 1 field
#> Geometry type: POLYGON
#> Dimension:     XY
```

```
#> Bounding box:  xmin: 8.414334 ymin: 3.208967 xmax: 8.938047 ymax: 3.788305
#> Geodetic CRS:  WGS 84
```

The edges of the island, and where people live, are not fixed in time, and datasets will disagree. LandScan, in particular, intentionally extends shoreline in order to include littoral human settlement.

3 Align to Grids from BIMEP GPS Data

This section uses the original BIMEP GPS data, so it's a longitude-latitude for each house an an integer count of the number of people. That means there is no section or area grid to consider. We align BIMEP to any location.

The output of this section is a set of GeoTIFFs in the `aligned` subdirectory of your data directory (likely `inst/extdata/aligned`). The names are of the form `<grid><resolution>_<source>.tif`. So the HRSL is on a 100m grid. It is saved as `HRSL100_HRSL.tif`. Bioko data on that grid is saved as `HRSL100_Bioko.tif`.

The incoming BIMEP data is GPS coordinates, and a few are farther in the ocean than one could comfortably kayak, so let's remove those. We spoke with the people who collected the data, and the few wild coordinates are GPS errors with particular devices.

```
bimep_points_file <- fs::path(data_dir, "bimep_gps.shp")
if (!file.exists(bimep_points_file)) {
  bimep_raw <- bimep_population_as_points()
  intersected_with_bioko <- sf::st_intersection(bimep_raw, bioko_sf)
  sf::st_write(intersected_with_bioko, bimep_points_file, driver = "ESRI Shapefile")
}
```

For each grid, the code to project the GPS data is the same.

```
bimep_on_grid <-
function(grid, bioko_sf, local_directory = "inst/extdata") {
  bimep_points_file <- fs::path(local_directory, "bimep_gps.shp")
  if (!file.exists(bimep_points_file)) {
    bimep_raw <- bimep_population_as_points()
    intersected_with_bioko <- sf::st_intersection(bimep_raw, bioko_sf)
    sf::st_write(intersected_with_bioko, bimep_points_file, driver = "ESRI Shapefile")
    features <- intersected_with_bioko
  } else {
    features <- sf::st_read(bimep_points_file)
  }
  geolocated_on_grid(grid, features, bioko_sf)
}
```

3.1 HRSL

The High-Resolution Settlement Layer marks where houses are with values and everywhere else with NA. The incoming data is for all of Equatorial Guinea, so we crop it. We need to have zeroes where there is land, so add the zeroes to the HRSL. Pixels with no land are marked NA. Otherwise, we don't modify values.

```
hrsl_raster <- read_hrsl(local_directory = data_dir)
hrsl_raster_crop <- raster::crop(hrsl_raster, bioko_sf, snap = "out")
hrsl_zero_mask <- raster::rasterize(bioko_sf, hrsl_raster_crop, field = 0)
hrsl_raster_zero <- raster::cover(hrsl_raster_crop, hrsl_zero_mask)
plot(hrsl_raster_zero)
```

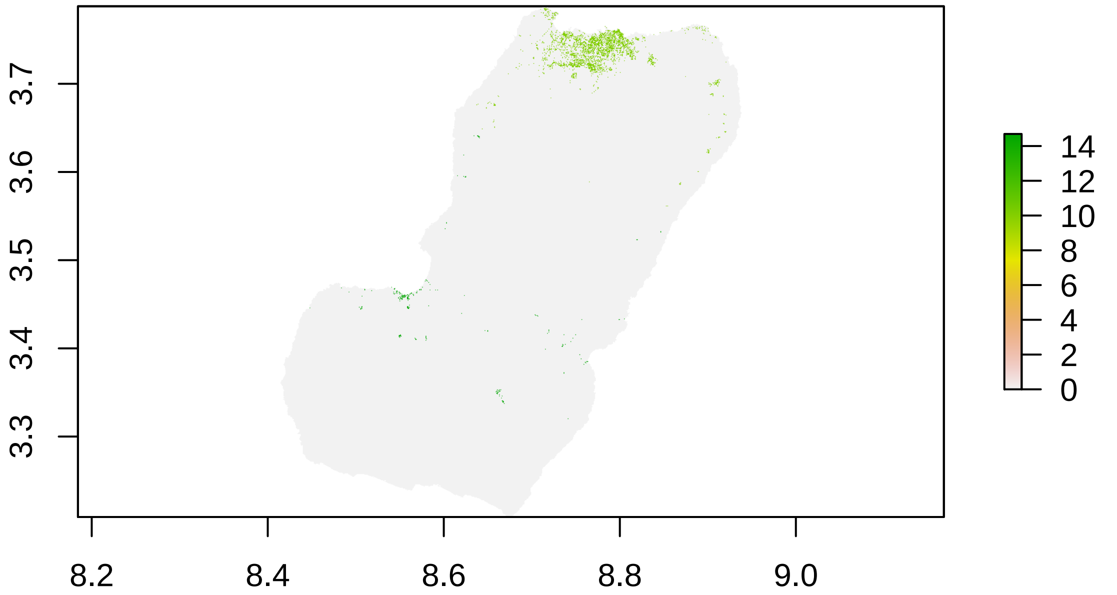

Now put Bioko data on that grid:

```
bimep_on_hrsl <- bimep_on_grid(hrsl_raster_zero, bioko_sf, local_directory = data_dir)
#> Reading layer `bimep_gps' from data source `/home/adolgert/dev/popbioko/inst/extdata/bin
#> Simple feature collection with 63337 features and 2 fields
#> Geometry type: POINT
#> Dimension:     XY
#> Bounding box:  xmin: 8.447933 ymin: 3.254711 xmax: 8.920785 ymax: 3.773996
#> Geodetic CRS:  GCS_unknown
plot(bimep_on_hrsl)
```

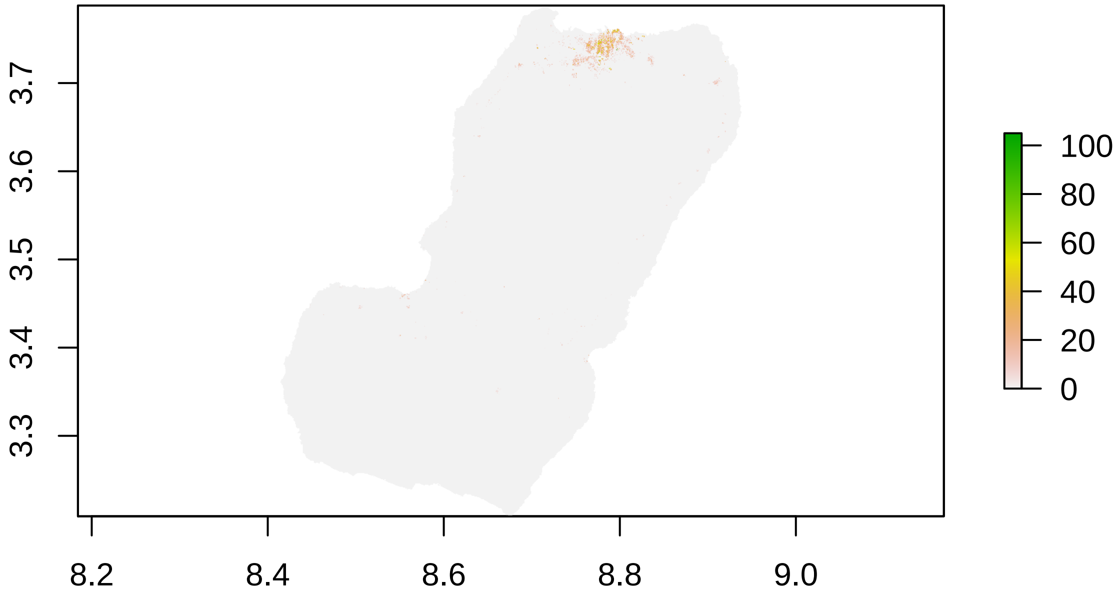


```
popbioko::write_aligned_raster(
  hrsl_raster_zero,
  list(source = "HRSL", grid = "HRSL", resolution = 30),
  local_directory = data_dir
)
popbioko::write_aligned_raster(
```

```
  bimep_on_hrsl,
  list(source = "BIMEP", grid = "HRSL", resolution = 30),
  local_directory = data_dir
)
```

For comparison with coarser datasets, we aggregate it. It turns out that the coarse datasets, at this latitude, have grid cells that are 924 m on a side.

```
aggregate_and_write <- function(raster, name) {
  pixel_area <- square_meters_per_pixel.raster(raster)
  desired_area <- 924^2  # The largest grid is 924x924, not 1km.
  fact <- round(sqrt(desired_area / pixel_area))
  cat(paste("aggregating factor for", name, "is", fact, "with pixel side", sqrt(pixel_area
  aggregated <- raster::aggregate(raster, fact = fact, fun = sum, expand = TRUE)
  agg_pixel_side <- sqrt(square_meters_per_pixel.raster(aggregated))
  popbioko::write_aligned_raster(
    aggregated,
    list(source = name$source, grid = name$grid, resolution = agg_pixel_side),
    local_directory = data_dir)
}
```

Write the HRSL for 1km.

```
aggregate_and_write(
  hrsl_raster_zero,
  list(source = "HRSL", grid = "HRSL", resolution = 30)
)
#> aggregating factor for HRSL is 30 with pixel side 30.789804349904
#>  aggregating factor for HRSL is 30 with pixel side 30.789804349904
#>  aggregating factor for 30 is 30 with pixel side 30.789804349904
```

Write BIMEP for 1km.

```
aggregate_and_write(
  bimep_on_hrsl,
  list(source = "BIMEP", grid = "HRSL", resolution = 30)
)
#> aggregating factor for BIMEP is 30 with pixel side 30.789804349904
#>  aggregating factor for HRSL is 30 with pixel side 30.789804349904
#>  aggregating factor for 30 is 30 with pixel side 30.789804349904
```

3.2 LandScan

LandScan is 1 km data, so we don't aggregate it.

```
landscan <- read_landscan(local_directory = data_dir)
landscan <- raster::crop(landscan, bioko_sf, snap = "out")
bimep_on_landscan <- bimep_on_grid(
  landscan, bioko_sf, local_directory = data_dir)
#> Reading layer `bimep_gps' from data source `/home/adolgert/dev/popbioko/inst/extdata/bim
#> Simple feature collection with 63337 features and 2 fields
#> Geometry type: POINT
#> Dimension:     XY
#> Bounding box:  xmin: 8.447933 ymin: 3.254711 xmax: 8.920785 ymax: 3.773996
#> Geodetic CRS:  GCS_unknown
popbioko::write_aligned_raster(
```

```
  landscan,
  list(source = "LandScan", grid = "LandScan", resolution = 1000),
  local_directory = data_dir
)
popbioko::write_aligned_raster(
  bimep_on_landscan,
  list(source = "BIMEP", grid = "LandScan", resolution = 1000),
  local_directory = data_dir
)
```

Actually, let's disaggregate it, to see what happens if we use its estimates on a 100m grid. There is no interpolation in this conversion, just disaggregation of a pixel into 100 smaller pixels.

```
factor <- 10
landscan100 <- raster::disaggregate(landscan, fact = factor) / factor^2
bimep_on_landscan100 <- bimep_on_grid(
  landscan100, bioko_sf, local_directory = data_dir)
#> Reading layer `bimep_gps' from data source `/home/adolgert/dev/popbioko/inst/extdata/bim
#> Simple feature collection with 63337 features and 2 fields
#> Geometry type: POINT
#> Dimension:     XY
#> Bounding box:  xmin: 8.447933 ymin: 3.254711 xmax: 8.920785 ymax: 3.773996
#> Geodetic CRS:  GCS_unknown
popbioko::write_aligned_raster(
  landscan100,
  list(source = "LandScan", grid = "LandScan", resolution = 100),
  local_directory = data_dir
)
popbioko::write_aligned_raster(
  bimep_on_landscan100,
  list(source = "BIMEP", grid = "LandScan", resolution = 100),
  local_directory = data_dir
)
```

3.3 WorldPop Unconstrained

WorldPop is 100m, like the HRSL data, so we aggregate it. This is the unconstrained, individual-country data. The "adjusted" version changes the total population to match the UN estimates.

```
wp_options <- list(raw = "gnq_ppp_2018.tif", adjusted = "gnq_ppp_2018_UNadj.tif")
worldpop <- raster::raster(fs::path(
  data_dir, "Equatorial_Guinea_100m_Population", wp_options[["adjusted"]]))
worldpop_na <- raster::crop(worldpop, bioko_sf, snap = "out")
worldpop_zero_mask <- raster::rasterize(bioko_sf, worldpop_na, field = 0)
worldpop <- raster::cover(worldpop_na, worldpop_zero_mask)
worldpop_id <- list(source = "WorldPop-U", grid = "WorldPop", resolution = 100)
bimep_id <- worldpop_id
bimep_id$source <- "BIMEP"
popbioko::write_aligned_raster(
  worldpop,
  worldpop_id,
  local_directory = data_dir
)
bimep_on_worldpop <- bimep_on_grid(
  worldpop, bioko_sf, local_directory = data_dir)
```

```
#> Reading layer `bimep_gps' from data source `/home/adolgert/dev/popbioko/inst/extdata/bim
#> Simple feature collection with 63337 features and 2 fields
#> Geometry type: POINT
#> Dimension:     XY
#> Bounding box:  xmin: 8.447933 ymin: 3.254711 xmax: 8.920785 ymax: 3.773996
#> Geodetic CRS:  GCS_unknown
popbioko::write_aligned_raster(
  bimep_on_worldpop,
  bimep_id,
  local_directory = data_dir
)
aggregate_and_write(
  worldpop,
  worldpop_id
)
#> aggregating factor for WorldPop-U is 10 with pixel side 92.369391712888
#>  aggregating factor for WorldPop is 10 with pixel side 92.369391712888
#>  aggregating factor for 100 is 10 with pixel side 92.369391712888
aggregate_and_write(
  bimep_on_worldpop,
  bimep_id
)
#> aggregating factor for BIMEP is 10 with pixel side 92.369391712888
#>  aggregating factor for WorldPop is 10 with pixel side 92.369391712888
#>  aggregating factor for 100 is 10 with pixel side 92.369391712888
```

Let's look at WorldPop Unconstrained, aka WorldPop-U.

```
tm_shape(worldpop) + tm_raster()
```

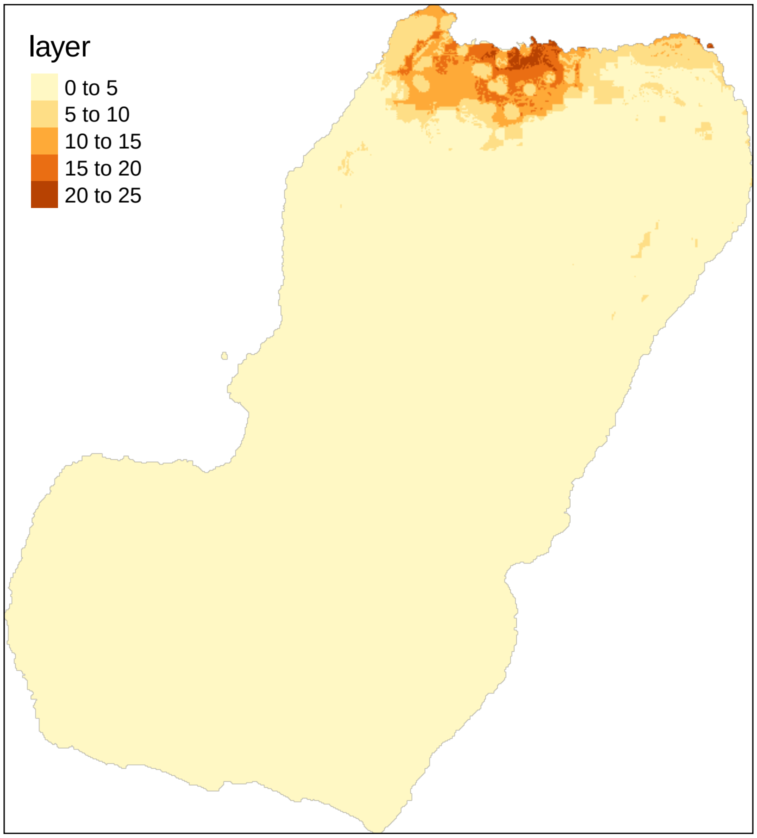


```r
tm_shape(bimep_on_worldpop) + tm_raster()
```

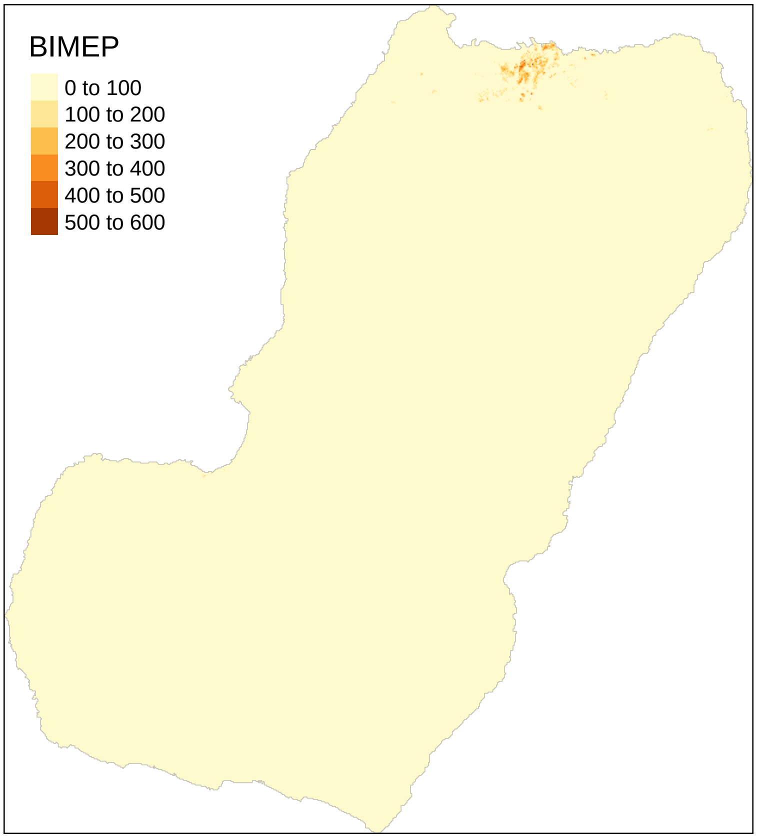

3.4 WorlPop Constrained

This WorldPop dataset is the constrained, individual-country data. Again, we use the version whose population is adjusted to the UN totals.

```
wp_options <- list(adjusted = "gnq_ppp_2020_UNadj_constrained.tif")
worldpop_constrained <- raster::raster(fs::path(
  data_dir, "worldpop_constrained", wp_options[["adjusted"]]))
worldpop_constrained_na <- raster::crop(worldpop_constrained, bioko_sf, snap = "out")
worldpop_c_zero_mask <- raster::rasterize(bioko_sf, worldpop_constrained_na, field = 0)
worldpop_constrained2020 <- raster::cover(worldpop_constrained_na, worldpop_c_zero_mask)
# Here we apply a growth rate to go from 2020 to 2018. It's rough but an improvement.
worldpop_constrained <- exp(-2 * growth_rate_per_year) * worldpop_constrained2020
worldpop_constrained_id <- list(source = "WorldPop-C", grid = "WorldPop", resolution = 100)
popbioko::write_aligned_raster(
  worldpop_constrained,
  worldpop_constrained_id,
  local_directory = data_dir
)
aggregate_and_write(
  worldpop_constrained,
  worldpop_constrained_id
)
#> aggregating factor for WorldPop-C is 10 with pixel side 92.369391712888
#>  aggregating factor for WorldPop is 10 with pixel side 92.369391712888
#>  aggregating factor for 100 is 10 with pixel side 92.369391712888
```

Plot WorldPop-C.

```
tm_shape(worldpop_constrained) + tm_raster()
```

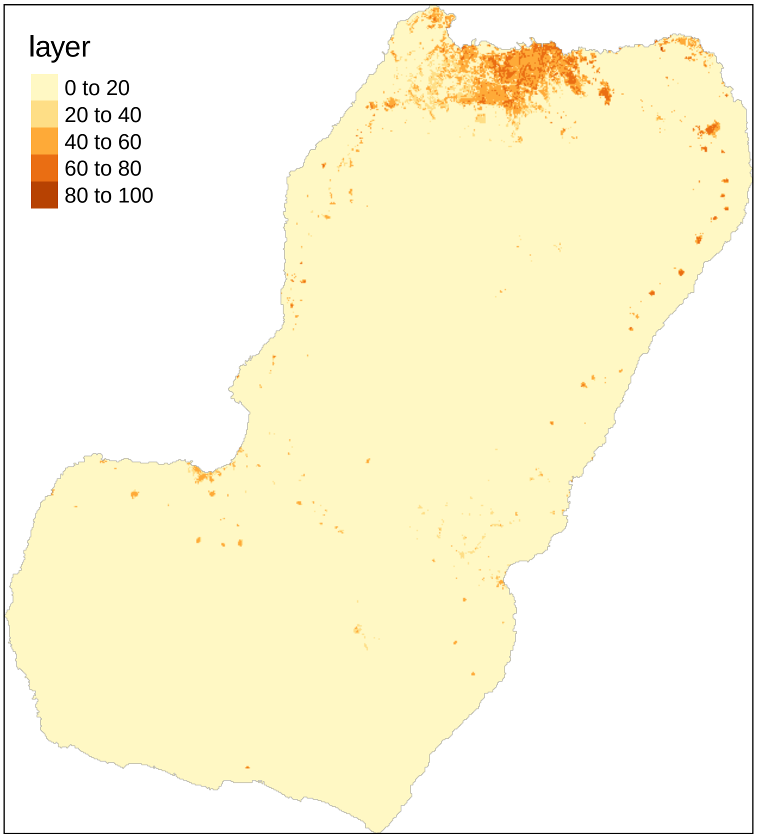

3.5 Gridded Population of the World

This is on the same grid as LandScan, so we won't need to map the BIMEP data to this grid.

```
gpw = raster::raster(fs::path(
  data_dir, "gpw", "gpw_v4_population_count_rev11_2020_30_sec.tif"))
gpw <- raster::crop(gpw, bioko_sf, snap = "out")
gpw_id <- list(source = "GPW", grid = "LandScan", resolution = 1000)
stopifnot(as.character(raster::crs(gpw)) == "+proj=longlat +datum=WGS84 +no_defs")
stopifnot(raster::extent(gpw) == raster::extent(landscan))
stopifnot(raster::res(gpw) == raster::res(landscan))
```

Disaggregate this like we did LandScan.

```
factor <- 10
gpw100 <- raster::disaggregate(gpw, fact = factor) / factor^2
popbioko::write_aligned_raster(
  gpw,
  gpw_id,
  local_directory = data_dir
)
popbioko::write_aligned_raster(
  gpw100,
  list(source = "GPW", grid = "LandScan", resolution = 100),
  local_directory = data_dir
```

```
)
```

Plot GPW.

```
tm_shape(gpw) + tm_raster()
```

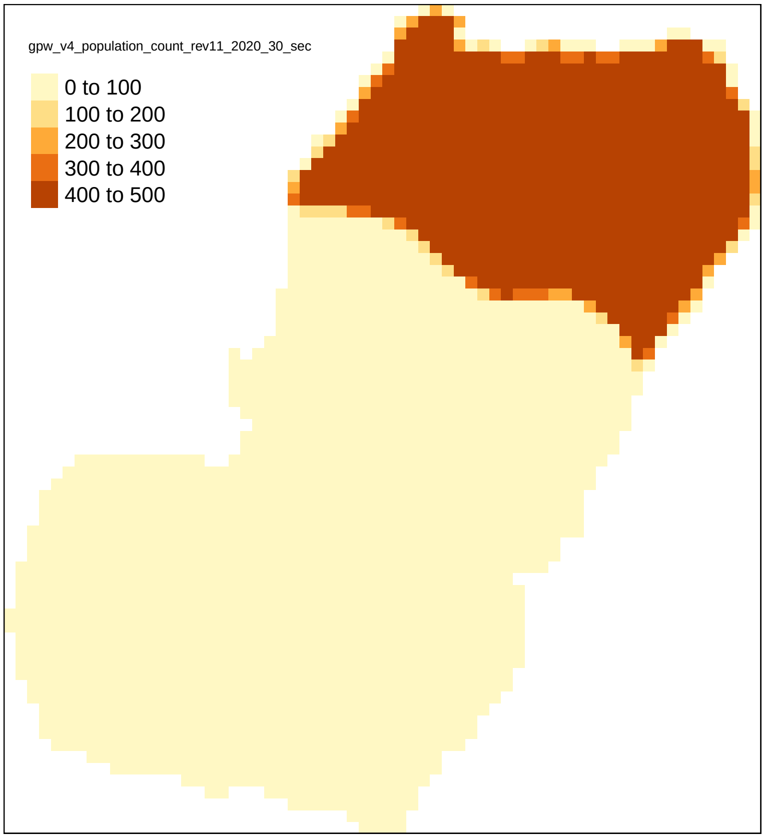

Measuring the accuracy of gridded human population density surfaces: a case study in Bioko Island, Equatorial Guinea: Supplement B

Brendan F Fries, Carlos A Guerra, et al

Contents

1 Overview **1**

2 Load Maps **2**

3 Summary Statistics **3**
3.1 Urban Fraction . . . 3
3.2 Construct the Summary Statistics Table . . . 5
3.3 Comparison Tables . . . 6
3.4 Error Bounds . . . 14

4 Population Scatter Plots **21**

5 Cumulative Maps **35**

6 Proportion, Accuracy, Recall, and Precision **41**
6.1 Define the values . . . 41
6.2 Proportion . . . 42
6.3 Precision . . . 44
6.4 Accuracy . . . 46
6.5 Recall . . . 47
6.6 Table of ranges . . . 49

7 Goodness of Fit Ratio **50**

8 Map Plots **54**

9 Figure 7: PfPR by Population **60**

From git@github.com:dd-harp/population_comparison_bioko.git on Sat May 22 11:02:24 2021, generated by adolgert.

1 Overview

Both BIMEP gold standard population data and maps from LandScan, WorldPop, GPW, and HRSL were aligned and formatted by the previous vignette. This notebook reads those datasets and calculates metrics from a population density map by comparing them with the BIMEP map.

2 Load Maps

The maps were aligned and formatted by the "get-data" vignette. We load them all at once here for convenience. The naming scheme is dataset-on-grid, where grids are 100m or 1km, according to where they come from.

These maps are estimates of the number of people in each pixel, not estimates of the density of people per square kilometer at the pixel location.

```r
data_dir <- params$data_directory
map_root <- fs::path(data_dir, "aligned")
files <- list.files(map_root, pattern = "*.tif$")
files_df <- filenames_to_description(files)
maps <- lapply(files, function(x) raster::raster(fs::path(map_root, x)))
names(maps) <- rownames(files_df)
files_df
```

	filename	name	resolution	source	grid
BIMEP on HRSL coarse	HRSL_coarse_BIMEP.tif	BIMEP on HRSL coarse	coarse	BIMEP	HRSL
HRSL on HRSL coarse	HRSL_coarse_HRSL.tif	HRSL on HRSL coarse	coarse	HRSL	HRSL
BIMEP on HRSL fine	HRSL_fine_BIMEP.tif	BIMEP on HRSL fine	fine	BIMEP	HRSL
HRSL on HRSL fine	HRSL_fine_HRSL.tif	HRSL on HRSL fine	fine	HRSL	HRSL
BIMEP on LandScan coarse	LandScan_coarse_BIMEP.tif	BIMEP on LandScan coarse	coarse	BIMEP	LandScan
GPW on LandScan coarse	LandScan_coarse_GPW.tif	GPW on LandScan coarse	coarse	GPW	LandScan
LandScan on LandScan coarse	LandScan_coarse_LandScan.tif	LandScan on LandScan coarse	coarse	LandScan	LandScan
BIMEP on LandScan fine	LandScan_fine_BIMEP.tif	BIMEP on LandScan fine	fine	BIMEP	LandScan
GPW on LandScan fine	LandScan_fine_GPW.tif	GPW on LandScan fine	fine	GPW	LandScan
LandScan on LandScan fine	LandScan_fine_LandScan.tif	LandScan on LandScan fine	fine	LandScan	LandScan
BIMEP on WorldPop coarse	WorldPop_coarse_BIMEP.tif	BIMEP on WorldPop coarse	coarse	BIMEP	WorldPop
WorldPop-C on WorldPop coarse	WorldPop_coarse_WorldPop-C.tif	WorldPop-C on WorldPop coarse	coarse	WorldPop-C	WorldPop
WorldPop-U on WorldPop coarse	WorldPop_coarse_WorldPop-U.tif	WorldPop-U on WorldPop coarse	coarse	WorldPop-U	WorldPop
BIMEP on WorldPop fine	WorldPop_fine_BIMEP.tif	BIMEP on WorldPop fine	fine	BIMEP	WorldPop
WorldPop-C on WorldPop fine	WorldPop_fine_WorldPop-C.tif	WorldPop-C on WorldPop fine	fine	WorldPop-C	WorldPop
WorldPop-U on WorldPop fine	WorldPop_fine_WorldPop-U.tif	WorldPop-U on WorldPop fine	fine	WorldPop-U	WorldPop

There are 16 rows for (5 data sources) x (2 resolutions each) x (data source and BIMEP comparison).

Maps from BIMEP are sometimes in projection using Universal Transverse Mercator (UTM) for zone 32N.

```
utm_projection <- "+proj=utm +zone=32N +ellps=WGS84  +no_defs +units=m +datum=WGS84"
bioko_sf <- sf::st_read(fs::path(data_dir, "source", "bioko.shp"))
```

```
## Reading layer `bioko' from data source `/home/adolgert/dev/popbioko/inst/extdata/source/
## Simple feature collection with 1 feature and 1 field
## Geometry type: POLYGON
## Dimension:     XY
## Bounding box:  xmin: 8.414334 ymin: 3.208967 xmax: 8.938047 ymax: 3.788305
## Geodetic CRS:  WGS 84
```

3 Summary Statistics

Table 1 is summary statistics, done at the pixel level, so let's walk through those here.

3.1 Urban Fraction

There isn't a single, standard definition for urban fraction. Countries each choose their own ways to assess the important demographic movement from rural to urban. These assessments often combine population density, administrative boundaries, resource availability (like sewer and water), and functional use patterns. The baseline, however, is to use population density, by itself, using a lower bound of either 1000 people per square kilometer or 1500 people per square kilometer.

We found one document where Equatorial Guinea defined urban population density as 1500 people per square kilometer, but there isn't an official cutoff and the more common limit is 1000 per square kilometer. That's 1000 people per pixel for a grid with 1 km pixels and 10 people per pixel for a grid with 100 m pixels. This is equivalent to 0.001 people per meter squared. You'll see that we look at both below.

It can be complicated to estimate population density, even though these datasets are all counts of people per grid square. Here are three examples of how to estimate urban fraction.

1. Count each pixel that reaches the threshold.
2. Count each pixel that has 1000 people within a circle whose area is a square kilometer.
3. Use a kernel density estimator to treat pixels as samples from a population density surface.

The distinction matters because these datasets range in resolution from 30m to 924m on a side. The 30m is almost point data. What we see below is that the second and third methods give very similar numbers for this dataset, across all resolutions.

3.1.1 Raw Urban Fraction

We measure pixel threshold as a number of people per square meter. We need to know the size of each pixel in order to do that. It helps to know how many pixels we're talking about, so we return the numerator and denominator of each fraction.

```
urban_fraction_population <-
function(density_raster, urban_per_kilometer_sq) {
  urban_per_meter_sq <- urban_per_kilometer_sq / 10^6
  urban_per_pixel_sq <- urban_per_meter_sq * square_meters_per_pixel.raster(density_raster)
  vals <- raster::getValues(density_raster)
  c(sum(vals > urban_per_pixel_sq, na.rm = TRUE), sum(!is.na(vals)))
}
```

We can reuse this function for the kernel density estimation by applying it to the estimated density instead of the raw density.

3.1.2 Kernel Density Estimation

Kernel density estimation is a way to determine density from a point set. We have a grid, not points, so we take a few steps.

1. Convert the grid into a rate per unit area.
2. Sample points from that grid using a poisson process for each cell.
3. Project the points into a plane measured in meters.
4. Estimate percent urban from the resulting points.

This work will use the `raster` and `spatstat` packages for statistics, and it will use the `proj4` package to project the points from latitude-longitude to UTM.

We pull the values.

```
pop_raster <- maps[["LandScan on LandScan coarse"]]
sum(raster::values(pop_raster) > 1500, na.rm = TRUE) / nrow(pop_raster) / ncol(pop_raster)
```

```
## [1] 0.006919643
```

Then estimate the count.

```
population_count_estimator <-
function(projected_raster) {
  # Make a point pattern
  raster_sum <- as.integer(raster::cellStats(projected_raster, stat = "sum", na.rm = TRUE))
  pop_raster_im <- maptools::as.im.RasterLayer(projected_raster)
  point_pattern <- spatstat::rpoint(raster_sum, pop_raster_im)
  stopifnot(point_pattern$n == raster_sum)
  density <- spatstat::density.ppp(
    point_pattern,
    sigma = spatstat::bw.diggle,
    dimyx = c(raster::ncol(projected_raster), raster::nrow(projected_raster))
    )
  density * raster_sum / sum(density)
}
```

And look at point density.

It will help to use the Bioko outlines for to ensure the statistical process respects the island boundaries. It's called windowing in spatstat. You need to translate sf to sp, to sp geometry, to spatstat owin format.

```
bioko_sp <- as(bioko_sf, Class = "Spatial")
bioko_sp_polygon <- as(bioko_sp, "SpatialPolygons")
# Cannot make an owin from an unprojected space. Apparently.
bioko_proj_sf <- sf::st_transform(bioko_sf, crs = utm_projection)
# The projected space will have points outside. Let's make a little
# buffer to catch those points.
meters <- 1
bioko_buffer_sf <- sf::st_buffer(bioko_proj_sf, 500 * meters)
bioko_proj_sp <- as(bioko_proj_sf, Class = "Spatial")
bioko_proj_polygon_sp <- as(bioko_proj_sp, "SpatialPolygons")
bioko_owin <- as.owin(bioko_proj_polygon_sp)
```

Landscan measures people in thousands, so urban is pixels above 10^{-6}. The population density is on a map in meters, so let's count the pixel size in square meters.

Let's make population maps for every incoming map. This step approximates the map values by projecting them from lat-long to UTM coordinates. It uses bilinear interpolation. We avoid this kind of approximation

through most of our work. This step is less sensitive to interpolation because it will construct point process models during the `population_count_estimator`.

```
kde_dir <- fs::path(params$data_directory, "kde_raster")
if (!dir.exists(kde_dir)) {
  dir.create(kde_dir)
}
for (map_idx in 1:nrow(files_df)) {
  filename <- fs::path(kde_dir, files_df[map_idx, "filename"])
  if (!file.exists(filename)) {
    # or method = "ngb" for nearest-neighbor
    proj_raster <- raster::projectRaster(maps[[map_idx]], crs = utm_projection, method = "b
    # Bilinear interpolation, which can cause negative values.
    proj_raster <- raster::clamp(proj_raster, lower = 0)
    pop_density_im <- population_count_estimator(proj_raster)
    pop_density_raster <- im_to_raster(pop_density_im, utm_projection)
    raster::writeRaster(pop_density_raster, filename = filename, format = "GTiff")
  }
}
```

This makes density maps by drawing a 1 km-square circle around each pixel and summing population in that circle. By using the spatstat fourier transform functions, the algorithm effectively integrates through pixels.

```
density_dir <- fs::path(params$data_directory, "density_raster")
if (!dir.exists(density_dir)) {
  dir.create(density_dir)
}
for (map_idx in 1:nrow(files_df)) {
  filename <- fs::path(density_dir, files_df[map_idx, "filename"])
  if (!file.exists(filename)) {
    # or method = "ngb" for nearest-neighbor
    proj_raster <- raster::projectRaster(maps[[map_idx]], crs = utm_projection, method = "n
    pop_density_raster <- density_from_disc(proj_raster)
    raster::writeRaster(pop_density_raster, filename = filename, format = "GTiff")
  }
}
```

The density maps are saved as GeoTIFFs in the `density_raster` subdirectory, so load them here before making the summary table. *Loading from `density_dir` means we are using values calculated on a disc.*

```
density <- lapply(files_df$filename, function(x) raster::raster(fs::path(density_dir, x)))
names(density) <- names(maps)
```

3.2 Construct the Summary Statistics Table

This function makes the first table in the paper. It makes data for two urban densities, 1000 people per square km and 1500 people per square km. We'll compare results to see whether they say anything different about the data.

```
summary_help <- function(urban_cutoff) {
  function(map_idx) {
    popbioko::summary_statistics(
      maps[[map_idx]], density[[map_idx]], urban_per_kilometer_sq = urban_cutoff
      )
  }
```

```
}

make_summary_df <- function(urban_cutoff) {
  summary_stats <- lapply(1:length(maps), summary_help(urban_cutoff))
  summary_list <- do.call(rbind, summary_stats)
  rownames(summary_list) <- names(maps)
  summary_df <- as.data.frame(summary_list)
  summary_df["name"] <- rownames(summary_list)
  summary_df <- merge(summary_df, files_df, by = "name")
}
summary1000_df <- make_summary_df(1000)
summary1500_df <- make_summary_df(1500)
names(summary1500_df)
```

```
##  [1] "name"             "total"            "side_meters"      "maximum"
##  [5] "max_density"      "empty_percent"    "pareto_fraction" "urban_num"
##  [9] "urban_den"        "urban_raw"        "urban_fit_num"    "urban_fit_den"
## [13] "urban_fit"        "na_percent"       "filename"         "resolution"
## [17] "source"           "grid"
```

Those dataframes are both strings and numeric. When we rotate them, they will be all strings, so let's do the conversion by hand.

```
show1000_df <- significant_digit_display(summary1000_df)
show1500_df <- significant_digit_display(summary1500_df)
```

3.3 Comparison Tables

The dataframes just created have many metrics for many datasets. This section walks through different comparisons in order to get a better look.

3.3.1 Compare two urban fractions

We're looking for trends that show there are fewer urban areas when we raise the urban criteria from 1000 people to 1500 people per square kilometer.

```
as.data.frame(t(show1000_df[show1000_df$grid == "HRSL", ]))
```

	1	2	9	10
source	BIMEP	BIMEP	HRSL	HRSL
grid	HRSL	HRSL	HRSL	HRSL
resolution	923.7	30.8	923.7	30.8
total	239056	239056	231210	231210
max_density	19909	22212	6304	7525
empty_percent	89.16	99.13	86.39	98.92
na_percent	45.17	47.86	45.17	47.86
urban_raw	1.902	0.8733	3.06	1.076
urban_fit	1.902	2.029	3.06	3.283
pareto_fraction	3.763	3.904	5.749	5.643

```
as.data.frame(t(show1500_df[show1500_df$grid == "HRSL", ]))
```

	1	2	9	10
source	BIMEP	BIMEP	HRSL	HRSL
grid	HRSL	HRSL	HRSL	HRSL
resolution	923.7	30.8	923.7	30.8
total	239056	239056	231210	231210
max_density	19909	22212	6304	7525
empty_percent	89.16	99.13	86.39	98.92
na_percent	45.17	47.86	45.17	47.86
urban_raw	1.613	0.8254	2.481	1.076
urban_fit	1.613	1.637	2.481	2.665
pareto_fraction	3.763	3.904	5.749	5.643

3.3.2 Compare maps of raw and estimated urban fractions

We would like to see whether the estimated urban fraction has a reasonable-looking Gaussian kernel size.

```
sq_m_per <- c(square_meters_per_pixel.raster(maps[[2]]), square_meters_per_pixel.raster(der
sq_m_per
```

```
## [1] 853212.7 852846.0
```

```
cutoffs <- data.frame(urban_per_meter_sq = c(1000, 1500) / 10^6)
cutoffs["raw"] <- cutoffs[["urban_per_meter_sq"]] * square_meters_per_pixel.raster(maps[[2]
cutoffs["density"] <- cutoffs[["urban_per_meter_sq"]] * square_meters_per_pixel.raster(dens
cutoffs
```

urban_per_meter_sq	raw	density
0.0010	853.2127	852.846
0.0015	1279.8190	1279.269

Plot these with cutoffs to see that the maps are similar.

```
plot(maps[[2]] / cutoffs[1, "raw"])
```

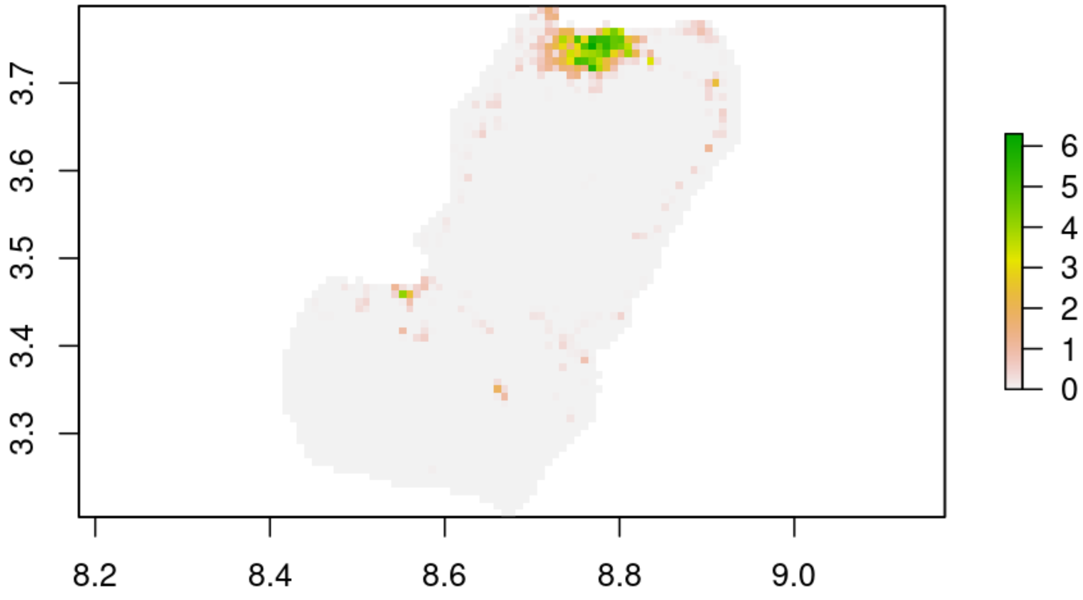


```
plot(maps[[2]] / cutoffs[2, "raw"])
```

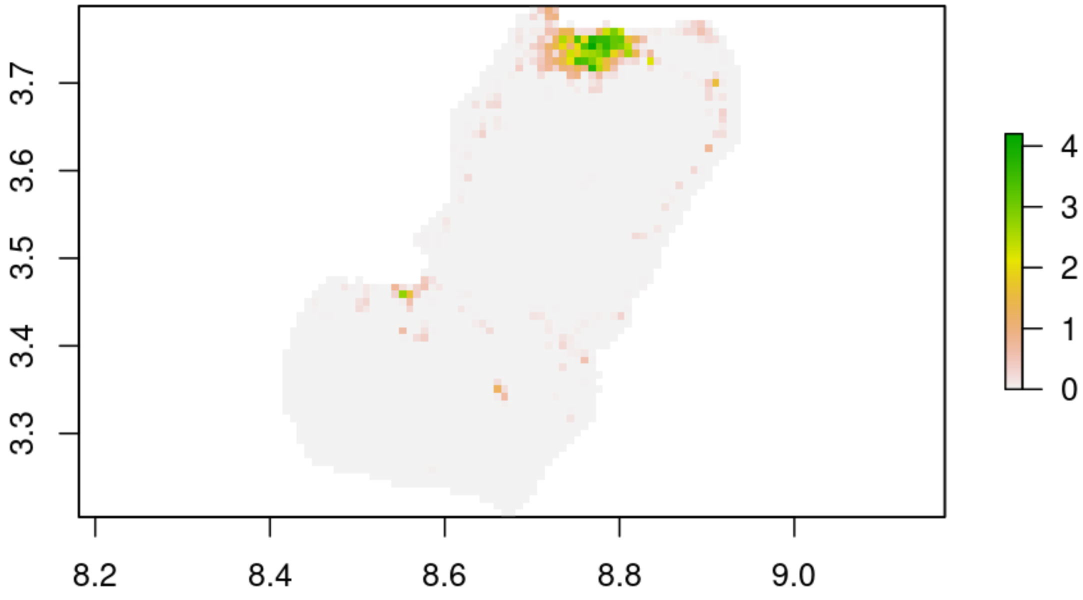


```
plot(density[[2]] / cutoffs[1, "density"])
```

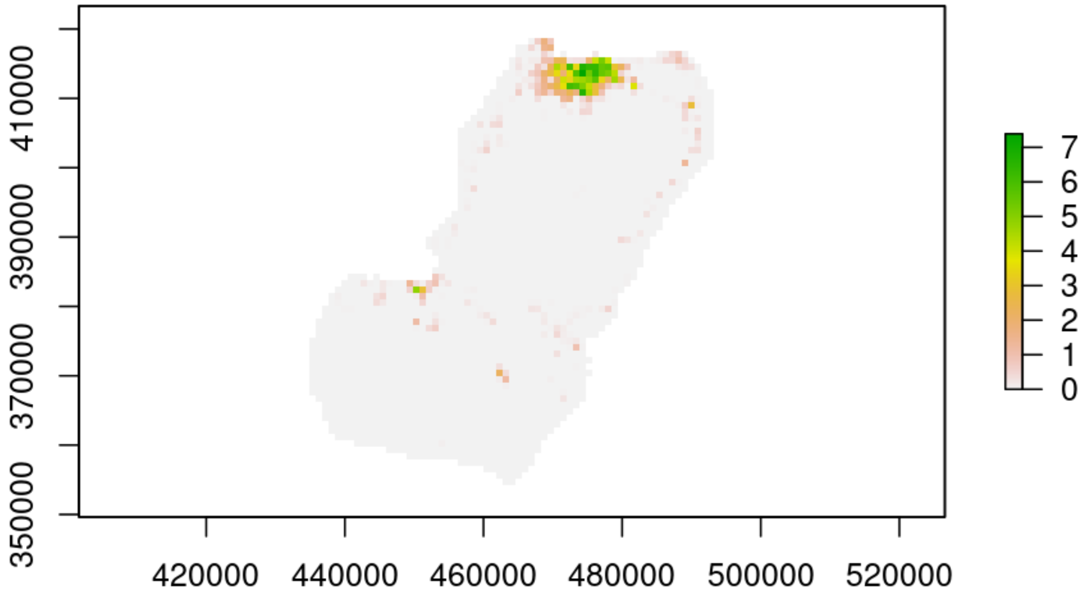


```r
plot(density[[2]] / cutoffs[2, "density"])
```

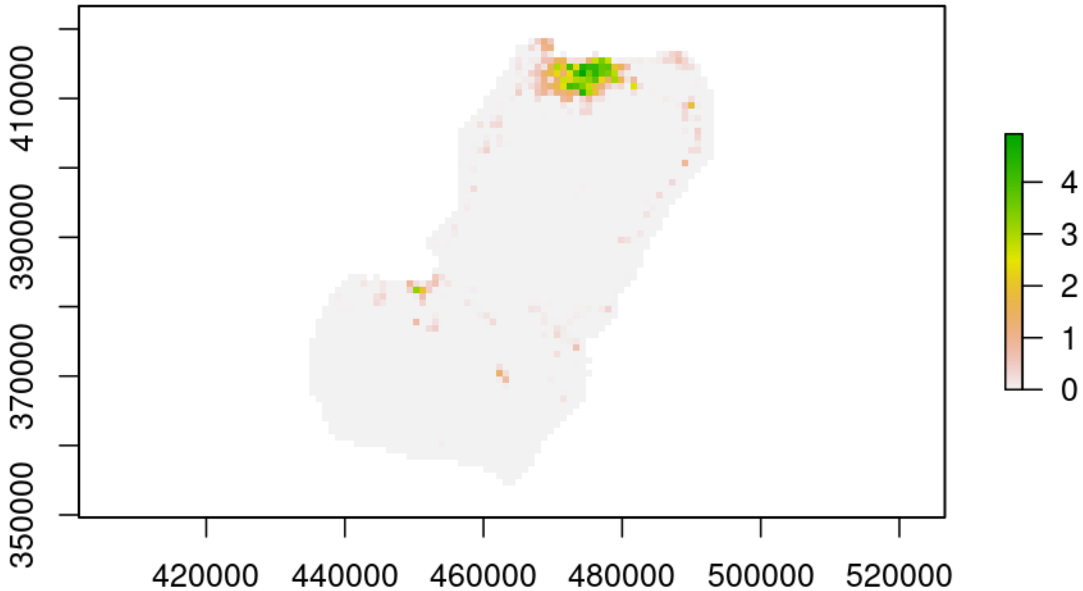

3.3.3 Compare across source grids

This shows all HRSL grid together, then all WorldPop, then all LandScan.

```
as.data.frame(t(show1000_df[show1000_df$grid == "HRSL", ]))
```

	1	2	9	10
source	BIMEP	BIMEP	HRSL	HRSL
grid	HRSL	HRSL	HRSL	HRSL
resolution	923.7	30.8	923.7	30.8
total	239056	239056	231210	231210
max_density	19909	22212	6304	7525
empty_percent	89.16	99.13	86.39	98.92
na_percent	45.17	47.86	45.17	47.86
urban_raw	1.902	0.8733	3.06	1.076
urban_fit	1.902	2.029	3.06	3.283
pareto_fraction	3.763	3.904	5.749	5.643

```
as.data.frame(t(show1000_df[show1000_df$grid == "WorldPop", ]))
```

	5	6	13	14	15	16
source	BIMEP	BIMEP	WorldPop-C	WorldPop-C	WorldPop-U	WorldPop-U
grid	WorldPop	WorldPop	WorldPop	WorldPop	WorldPop	WorldPop
resolution	923.7	92.4	923.7	92.4	923.7	92.4
total	239056	239056	365265	365265	362962	362962

	5	6	13	14	15	16
max_density	20305	20992	6842	7139	2356	2547
empty_percent	88.76	98.06	83.13	96.63	0.04114	0.1112
na_percent	45.31	48.05	45.31	48.05	44.88	47.48
urban_raw	1.907	1.463	4.436	3.367	3.826	3.951
urban_fit	1.907	2.041	4.436	4.772	3.826	4.272
pareto_fraction	3.731	3.945	7.794	7.812	22.05	22.29

```
as.data.frame(t(show1000_df[show1000_df$grid == "LandScan", ]))
```

	3	4	7	8	11	12
source	BIMEP	BIMEP	GPW	GPW	LandScan	LandScan
grid	LandScan	LandScan	LandScan	LandScan	LandScan	LandScan
resolution	923.7	92.4	923.7	92.4	923.7	92.4
total	239056	239056	365766	365766	218044	218044
max_density	20611	21144	565	565	34344	30640
empty_percent	88.53	98.09	0.2876	0.2876	56.29	56.29
na_percent	48.82	49.15	45.67	45.67	48.37	48.37
urban_raw	2.05	1.431	0	0	2.075	2.075
urban_fit	2.05	2.041	0	0	2.075	2.049
pareto_fraction	3.925	3.945	23.79	23.79	5.275	6.244

3.3.4 Compare across resolutions

This groups data with the same resolution.

```
as.data.frame(t(show1000_df[as.numeric(show1000_df$resolution) < 500, ]))
```

	2	4	6	8	10	12	14	16
source	BIMEP	BIMEP	BIMEP	GPW	HRSL	LandScan	WorldPop-C	WorldPop-U
grid	HRSL	LandScan	WorldPop	LandScan	HRSL	LandScan	WorldPop	WorldPop
resolution	30.8	92.4	92.4	92.4	30.8	92.4	92.4	92.4
total	239056	239056	239056	365766	231210	218044	365265	362962
max_density	22212	21144	20992	565	7525	30640	7139	2547
empty_percent	99.13	98.09	98.06	0.2876	98.92	56.29	96.63	0.1112
na_percent	47.86	49.15	48.05	45.67	47.86	48.37	48.05	47.48
urban_raw	0.8733	1.431	1.463	0	1.076	2.075	3.367	3.951
urban_fit	2.029	2.041	2.041	0	3.283	2.049	4.772	4.272
pareto_fraction	3.904	3.945	3.945	23.79	5.643	6.244	7.812	22.29

```
as.data.frame(t(show1000_df[as.numeric(show1000_df$resolution) > 500, ]))
```

	1	3	5	7	9	11	13	15
source	BIMEP	BIMEP	BIMEP	GPW	HRSL	LandScan	WorldPop-C	WorldPop-U
grid	HRSL	LandScan	WorldPop	LandScan	HRSL	LandScan	WorldPop	WorldPop
resolution	923.7	923.7	923.7	923.7	923.7	923.7	923.7	923.7
total	239056	239056	239056	365766	231210	218044	365265	362962
max_density	19909	20611	20305	565	6304	34344	6842	2356
empty_percent	89.16	88.53	88.76	0.2876	86.39	56.29	83.13	0.04114
na_percent	45.17	48.82	45.31	45.67	45.17	48.37	45.31	44.88

	1	3	5	7	9	11	13	15
urban_raw	1.902	2.05	1.907	0	3.06	2.075	4.436	3.826
urban_fit	1.902	2.05	1.907	0	3.06	2.075	4.436	3.826
pareto_fraction	3.763	3.925	3.731	23.79	5.749	5.275	7.794	22.05

```r
display_order <- data.frame(
  source = c("BIMEP", "HRSL", "LandScan", "WorldPop-C", "WorldPop-U", "GPW", "BIMEP", "HRSL
  granularity = c(rep("coarse", 6), rep("fine", 4)),
  grid = c("HRSL", "HRSL", "LandScan", "WorldPop", "WorldPop", "LandScan", "HRSL", "HRSL",
)
short_source <- c(
  BIMEP = "BIMEP", HRSL = "HRSL", LandScan = "LS", "WorldPop-C" = "WP-C",
  "WorldPop-U" = "WP-U", GPW = "GPW")
show1000_df$granularity <- ifelse(as.numeric(show1000_df$resolution) < 500, rep("fine", nro
show1000_df
```

source	grid	resolution	total	max_density	empty_percent	cent_percent	urban_raw	urban_fit	pareto_fraction	granularity
BIMEP	HRSL	923.7	239056	19909	89.16	45.17	1.902	1.902	3.763	coarse
BIMEP	HRSL	30.8	239056	22212	99.13	47.86	0.8733	2.029	3.904	fine
BIMEP	LandScan	923.7	239056	20611	88.53	48.82	2.05	2.05	3.925	coarse
BIMEP	LandScan	92.4	239056	21144	98.09	49.15	1.431	2.041	3.945	fine
BIMEP	WorldPop	923.7	239056	20305	88.76	45.31	1.907	1.907	3.731	coarse
BIMEP	WorldPop	92.4	239056	20992	98.06	48.05	1.463	2.041	3.945	fine
GPW	LandScan	923.7	365766	565	0.2876	45.67	0	0	23.79	coarse
GPW	LandScan	92.4	365766	565	0.2876	45.67	0	0	23.79	fine
HRSL	HRSL	923.7	231210	6304	86.39	45.17	3.06	3.06	5.749	coarse
HRSL	HRSL	30.8	231210	7525	98.92	47.86	1.076	3.283	5.643	fine
LandScan	LandScan	923.7	218044	34344	56.29	48.37	2.075	2.075	5.275	coarse
LandScan	LandScan	92.4	218044	30640	56.29	48.37	2.075	2.049	6.244	fine
WorldPop-C	WorldPop	923.7	365265	6842	83.13	45.31	4.436	4.436	7.794	coarse
WorldPop-C	WorldPop	92.4	365265	7139	96.63	48.05	3.367	4.772	7.812	fine
WorldPop-U	WorldPop	923.7	362962	2356	0.04114	44.88	3.826	3.826	22.05	coarse
WorldPop-U	WorldPop	92.4	362962	2547	0.1112	47.48	3.951	4.272	22.29	fine

```r
display_order
```

source	granularity	grid
BIMEP	coarse	HRSL
HRSL	coarse	HRSL
LandScan	coarse	LandScan
WorldPop-C	coarse	WorldPop
WorldPop-U	coarse	WorldPop
GPW	coarse	LandScan
BIMEP	fine	HRSL
HRSL	fine	HRSL
WorldPop-C	fine	WorldPop

source	granularity	grid
WorldPop-U	fine	WorldPop

```
do1000_df <- merge(display_order, show1000_df, by = c("source", "granularity", "grid"), so
stopifnot(nrow(do1000_df) == nrow(display_order))
ord1000_df <- do.call(rbind, lapply(1:nrow(display_order), function(row_idx) {
  with(do1000_df, do1000_df[source == display_order[row_idx, "source"] & granularity == di
}))
col_ord_df <- ord1000_df[, c("source", "resolution", "total", "max_density", "empty_percent
col_ord_df$source <- short_source[col_ord_df$source]
col_ord_df
```

	source	resolution	total	max_density	empty_percent	urban_raw	pareto_fraction
1	BIMEP	923.7	239056	19909	89.16	1.902	3.763
4	HRSL	923.7	231210	6304	86.39	3.06	5.749
6	LS	923.7	218044	34344	56.29	2.075	5.275
7	WP-C	923.7	365265	6842	83.13	4.436	7.794
9	WP-U	923.7	362962	2356	0.04114	3.826	22.05
3	GPW	923.7	365766	565	0.2876	0	23.79
2	BIMEP	30.8	239056	22212	99.13	0.8733	3.904
5	HRSL	30.8	231210	7525	98.92	1.076	5.643
8	WP-C	92.4	365265	7139	96.63	3.367	7.812
10	WP-U	92.4	362962	2547	0.1112	3.951	22.29

Use this on command line: `knitr::kable(t(col_ord_df), format = "latex")`

3.4 Error Bounds

How much does the exact alignment of the grid matter? What would happen if the grids were shifted slightly? The gold data is point data. Grids that are shifted slightly will have slightly different values in the tables. So let's shift each major grid a bunch of times and see what the values are. This work was excluded from the paper for space.

```
# With side_len = 10, this can take two hours to run 100 realizations.
error_dir <- fs::path(params$data_directory, "errors")
if (!dir.exists(error_dir)) {
  dir.create(error_dir)
}
side_len <- 10
for (grid in c("landscan", "hrsl", "worldpop")) {
  stats_file <- fs::path(error_dir, paste(grid, "csv", sep = "."))
  if (!file.exists(stats_file)) {
    popbioko::summary_statistics_multiple_grids(grid, side_len, stats_file)
  } else {
    warning(paste("file already exists", stats_file))
  }
}
```

```
error_dir <- fs::path(params$data_directory, "errors")
csvs <- do.call(
  rbind,
  lapply(
```

```
    list.files(error_dir, pattern = ".csv$", full.names = TRUE),
    function(csv) {read.csv(csv, header = TRUE, stringsAsFactors = TRUE)}
    ),
)
```

Given the small size of the city, the urban fit could be particularly sensitive. What we see is that the mean doesn't change much but the more coarse grids have a larger standard deviation.

```
ggplot(csvs, aes(x = urban_fit, group = grid)) +
  geom_histogram(bins = 50) +
    facet_grid(grid ~ .)
```

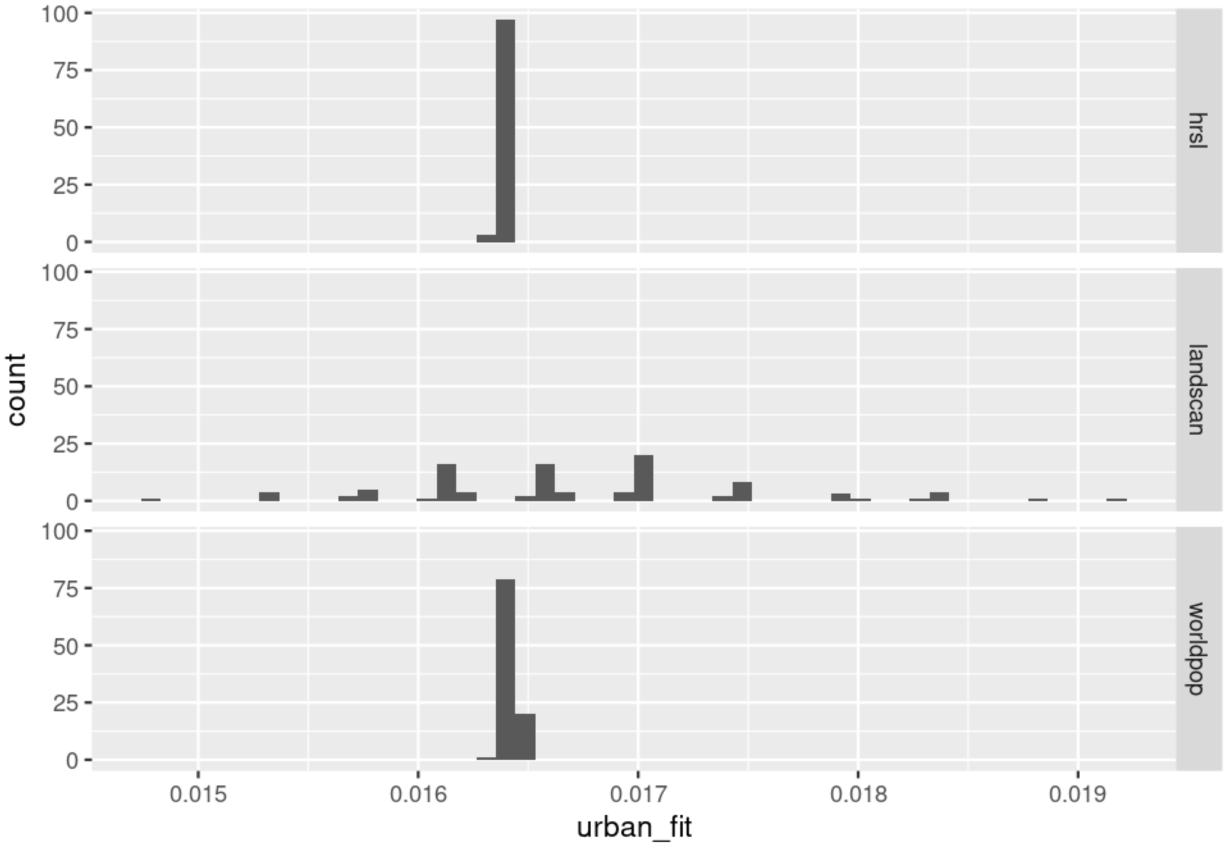

The percent of NA values is the size of the border around the island. This is kind of a control. We see it doesn't vary a lot.

```
ggplot(csvs, aes(x = na_percent, group = grid)) +
  geom_histogram(bins = 200) +
    facet_grid(grid ~ .)
```

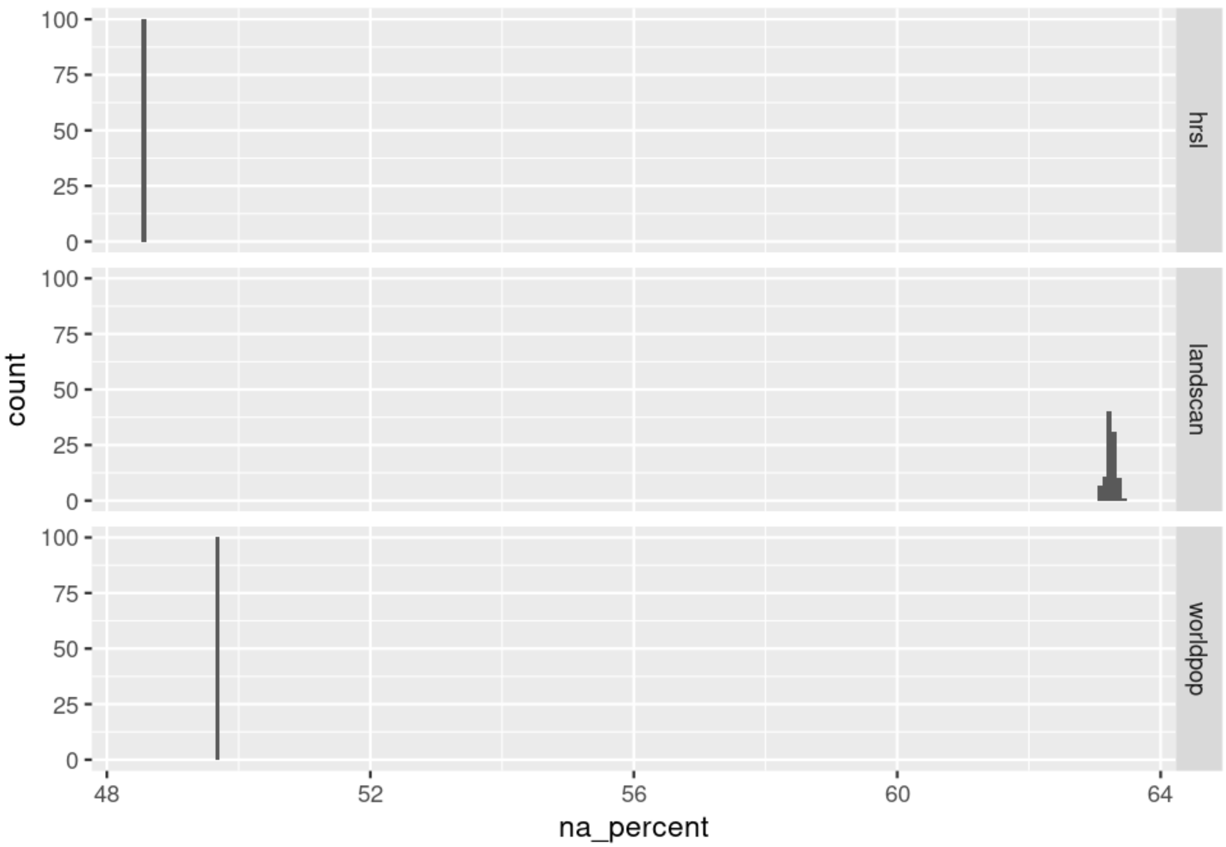

The Pareto fraction could also be sensitive because it looks at skewness in the distribution. Even for the coarsest grid, the values are fairly close to each other, and fine grids show very little variance.

```
ggplot(csvs, aes(x = pareto_fraction, group = grid)) +
  geom_histogram(bins = 200) +
    facet_grid(grid ~ .)
```

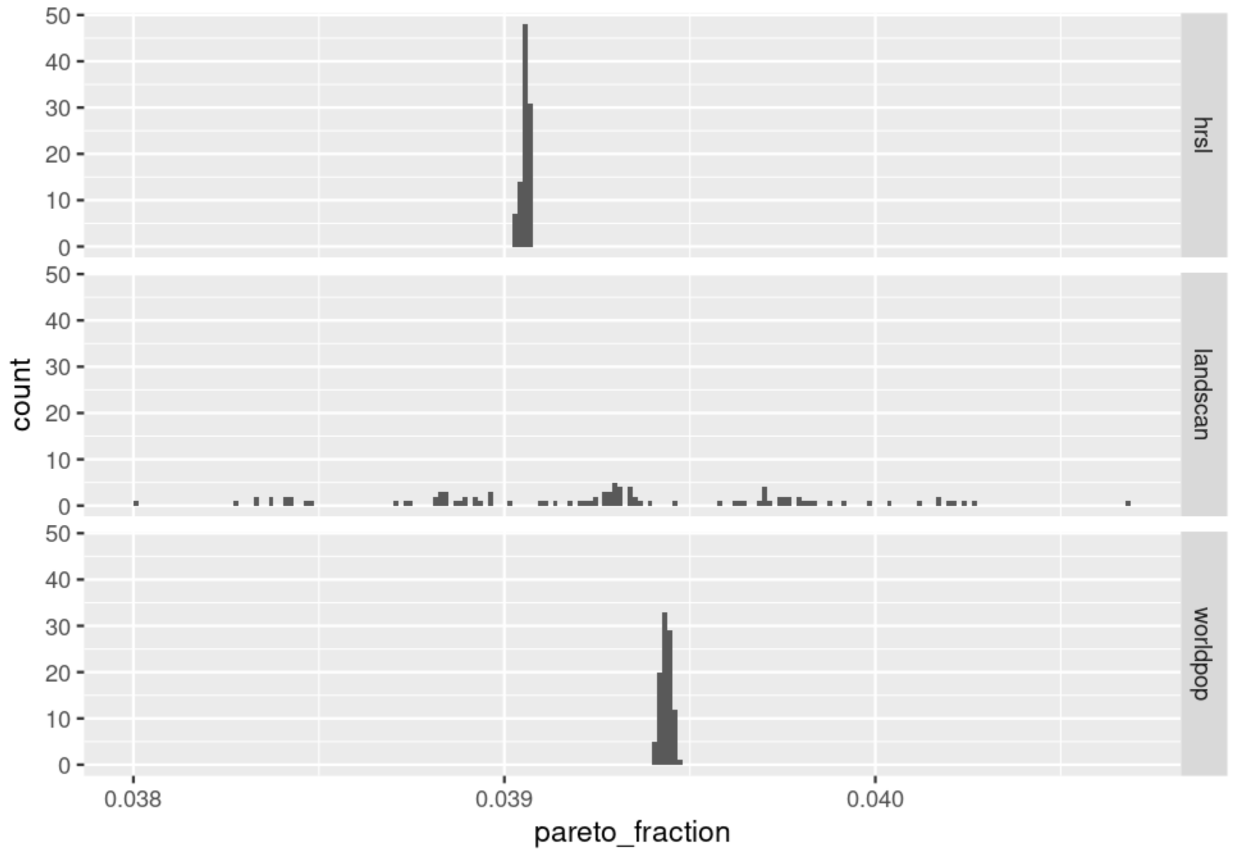

The maximum pixel value, of the population, not the population density. It looks like there's more noise in the coarse grid.

```
ggplot(csvs, aes(x = max_density, group = grid)) +
  geom_histogram(bins = 200) +
    facet_grid(grid ~ .)
```

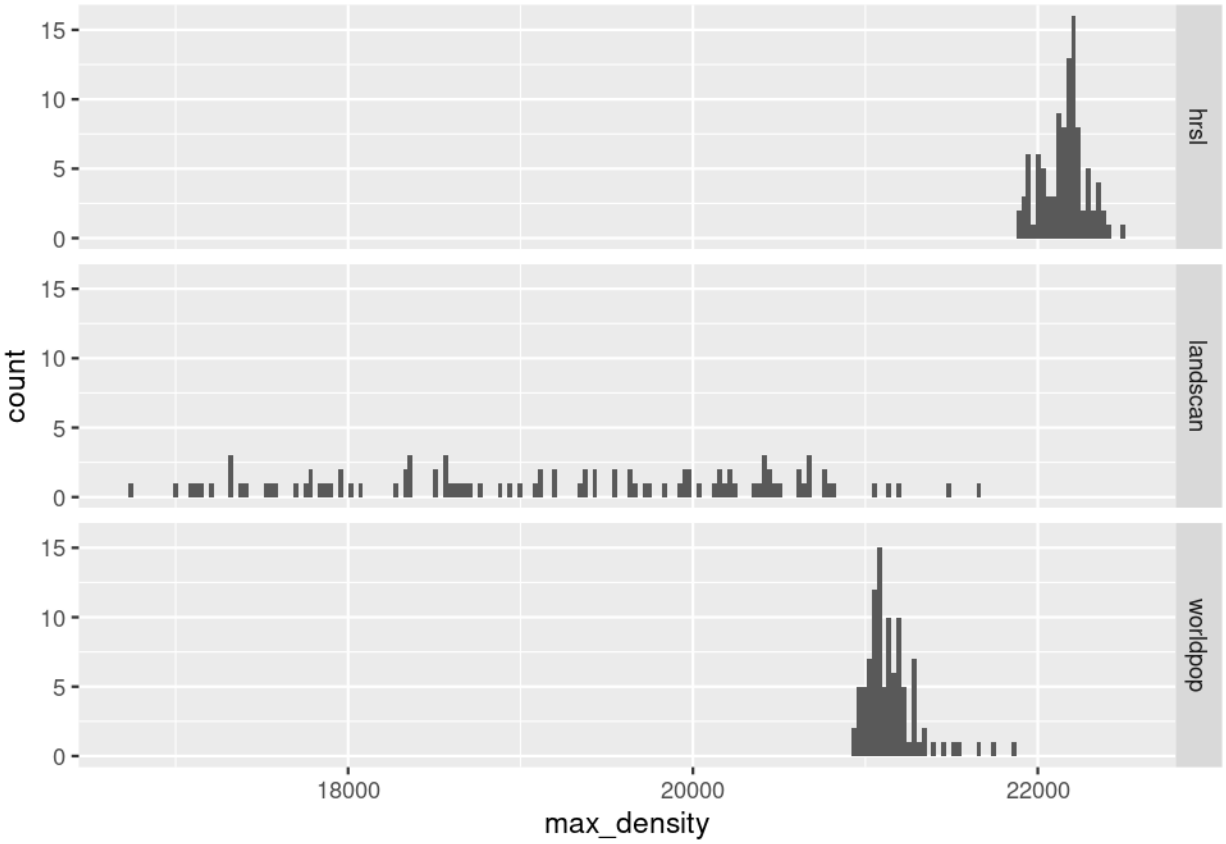

Let's look more closely by dividing by the mean in each case. We see that they all have about the same uncertainty in the maximum when you divide by the mean.

```
mean_max <- aggregate(max_density ~ grid, FUN = mean, data = csvs)
names(mean_max)[names(mean_max) %in% "max_density"] <- "mean_max"
with_mean <- merge(csvs, mean_max)
with_mean["nmax"] <- with_mean$max_density / with_mean$mean_max
ggplot(with_mean, aes(x = nmax, group = grid)) +
  geom_histogram(bins = 50) +
    facet_grid(grid ~ .)
```

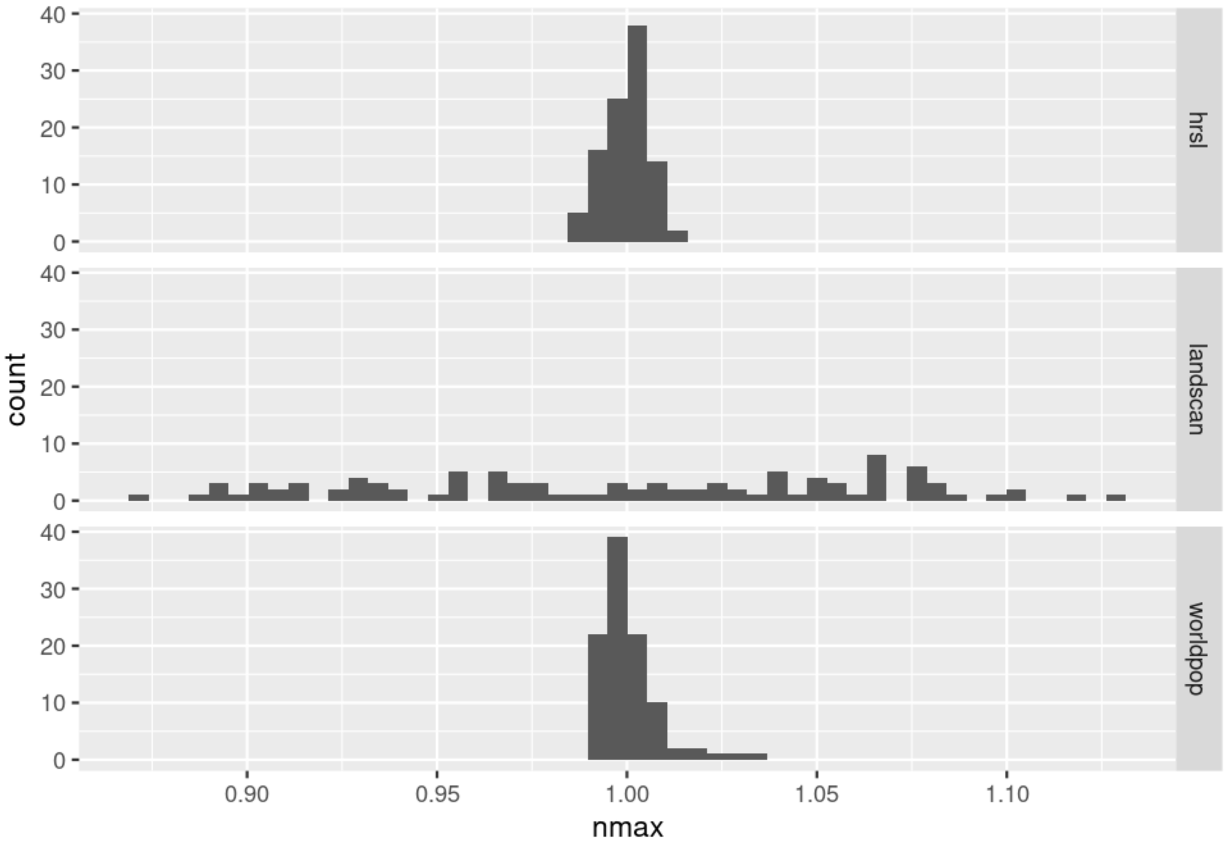

OK, so the errors aren't terribly skewed. We can use regular confidence intervals instead of bootstrapping. Maybe small values should use a Wald. Let's make values with confidence intervals for each of these datasets and take a look.

```
add_ci_to_column <- function(df, column) {
  names(df)[names(df) %in% column] <- "target"
  counted <- aggregate(target ~ grid, data = df, FUN = function(x) sum(!is.na(x)))
  names(counted)[names(counted) %in% "target"] <- "n"

  mean_col <- aggregate(target ~ grid, FUN = mean, data = df)
  names(mean_col)[names(mean_col) %in% "target"] <- "mean_val"

  sd_col <- aggregate(target ~ grid, FUN = sd, data = df)
  names(sd_col)[names(sd_col) %in% "target"] <- "sd_val"

  with_mean <- merge(merge(counted, mean_col), sd_col)
  lower <- paste(column, "low", sep = "_")
  upper <- paste(column, "up", sep = "_")
  with_mean[lower] <- with(with_mean, mean_val - 1.96 * sd_val / sqrt(n))
  with_mean[upper] <- with(with_mean, mean_val + 1.96 * sd_val / sqrt(n))
  names(with_mean)[names(with_mean) %in% "mean_val"] <- column
  with_mean[names(with_mean) %in% c(column, lower, upper, "grid")]
}

add_sd_to_column <- function(df, column) {
  names(df)[names(df) %in% column] <- "target"
  counted <- aggregate(target ~ grid, data = df, FUN = function(x) sum(!is.na(x)))
```

```r
  names(counted)[names(counted) %in% "target"] <- "n"

  mean_col <- aggregate(target ~ grid, FUN = mean, data = df)
  names(mean_col)[names(mean_col) %in% "target"] <- column

  sd_col <- aggregate(target ~ grid, FUN = sd, data = df)
  sd_name <- paste(column, "sd", sep = "_")
  names(sd_col)[names(sd_col) %in% "target"] <- sd_name
  together <- merge(mean_col, sd_col, by = "grid")
  together[names(together) %in% c(column, sd_name, "grid")]
}

zdf <- data.frame(
  rate = c(rnorm(10, mean = 0.5, sd = 0.2), rnorm(10, mean = 0.7, sd = 0.1)),
  grid = c(rep("HRSL", 10), rep("LandScan", 10)),
  stringsAsFactors = FALSE
)

# A little test
zzdf <- add_ci_to_column(zdf, "rate")
stopifnot(nrow(zzdf) == 2)
stopifnot(all(zzdf$rate_low < zzdf$rate))
stopifnot(all(zzdf$rate < zzdf$rate_up))

zzdf <- add_sd_to_column(zdf, "rate")
zzdf
```

grid	rate	rate_sd
HRSL	0.4477949	0.2415397
LandScan	0.7343114	0.1091575

```r
consider_error <- c("max_density", "empty_percent", "urban_fit", "pareto_fraction")
stopifnot(all(consider_error %in% names(csvs)))
single_ci <- function(col) add_sd_to_column(csvs, col)
ans_df <- single_ci(consider_error[1])
for (col in consider_error[2:length(consider_error)]) {
  ans_df <- merge(ans_df, single_ci(col))
}
t(ans_df)
```

```
##                     [,1]           [,2]           [,3]
## grid                "hrsl"         "landscan"     "worldpop"
## max_density         "22153.31"     "19184.18"     "21149.80"
## max_density_sd      " 124.5085"    "1239.8245"    " 160.1162"
## empty_percent       "99.12567"     "88.44104"     "98.08220"
## empty_percent_sd    "0.001459251"  "0.172281247"  "0.008865416"
## urban_fit           "0.01636731"   "0.01674714"   "0.01641798"
## urban_fit_sd        "6.877591e-06" "7.954545e-04" "2.703839e-05"
## pareto_fraction     "0.03905708"   "0.03927917"   "0.03943666"
## pareto_fraction_sd  "1.101992e-05" "5.357613e-04" "1.376481e-05"
```

In general, the 100m and 30m data behave more like point data. The point smoothing makes a big difference for their urban numbers. Overall, kernel density estimation was most consistent over all datasets, but it took

a day or two of computation the way we did it.

4 Population Scatter Plots

This section makes figure 4, scatter plots of population data versus ground truth population data. The plots of density use maps of either resolution to plot estimated density per square km. The plots of population size instead use the count of people in a pixel.

Most of the work here is getting consistent colors for datasets, allowing choice for point size and opacity, and setting log scales that contain all the data.

```
log_offset <- function(offset) {
  function(x) {
    log(x + offset)
  }
}
```

```
logoff <- log_offset(1)
symbol_size <- c(0.5, 0.25)
names(symbol_size) <- c("coarse", "fine")
symbol_choice <- c(19, 15)
names(symbol_choice) <- c("coarse", "fine")

check_name <- function(value, which, res, source) {
  if (length(value) != 1) {
    cat(sprintf("Could not find %s-%s-%s\n", which, res, source))
  }
  value
}

map_from_source_resolution <- function(source, resolution, map_list) {
  grid_to_use <- unique(files_df[files_df$source == source, "grid"])
  xy_df <- files_df[files_df$resolution == resolution & files_df$grid == grid_to_use, ]
  x_map_name <- check_name(xy_df[xy_df$source == "BIMEP", "name"], "bimep", resolution, sou
  y_map_name <- check_name(xy_df[xy_df$source == source, "name"], "y", resolution, source)
  gv <- function(a_map_name) {
    if (!a_map_name %in% names(map_list)) {
      msg <- sprintf("%s not in map names", a_map_name)
      cat(paste(msg, "\n"))
      stop(msg)
    }
    raster::getValues(map_list[[a_map_name]])
  }
  x <- gv(x_map_name)
  y <- gv(y_map_name)
  list(x = logoff(x), y = logoff(y))
}

#' This gets population counts, not density.
xy_from_source_resolution <- function(source, resolution) {
  map_from_source_resolution(source, resolution, maps)
}

density_from_source_resolution <- function(source, resolution) {
```

```
  map_from_source_resolution(source, resolution, density)
}

#' This is r^2 for a model with y = 1 * x + 0.
#' That's our pixel model. You don't want to fit with y = m * x + 0.
#' That would be the wrong model, so calculate our own r^2.
r_squared <- function(xy) {
  # NA are supposed to be in x and y to represent empty cells.
  xyfinite = !(is.na(xy$x) | is.na(xy$y))
  x <- xy$x[xyfinite]
  y <- xy$y[xyfinite]
  ss_total <- sum((y - mean(x))^2)
  ss_res <- sum((y - x)^2)
  1 - ss_res / ss_total
}

pop_scatter <- function(
  y_name, resolution, labels, title, maximum, background = NULL, counts = NULL,
  rsquared = 0
  ) {
  plot(
    vector(mode = "numeric", length = 0),
    xlim = c(0, maximum), ylim = c(0, maximum),
    xlab = labels[[1]], ylab = labels[[2]], main = title,
    xaxt = "n", yaxt = "n"
    )
  axis(1, logoff(10^c(0:5)), 10^c(0:5))
  axis(2, logoff(10^c(0:5)), 10^c(0:5))
  segments(logoff(0), logoff(0), logoff(10^5), logoff(10^5))
  segments(logoff(1), logoff(1), logoff(1), logoff(10^5), col = colors["grey"])
  segments(logoff(1), logoff(1), logoff(10^5), logoff(1), col = colors["grey"])

  coarsest_r2 <- list(res="none", value=NULL)
  for (plot_idx in 1:length(y_name)) {
    y_source <- y_name[plot_idx]
    y_resolution <- resolution[plot_idx]

    if (is.null(counts) && y_resolution == "fine") {
      data_func <- "density"
      data_function <- density_from_source_resolution
    } else {
      data_func <- "count"
      data_function <- xy_from_source_resolution
    }

    xy <- data_function(y_source, y_resolution)
    cex <- symbol_size[as.character(y_resolution)]
    pch <- symbol_choice[as.character(y_resolution)]
    if (resolution[plot_idx] == "coarse") {
      color <- colors[y_source]
    } else {
      color <- colors[y_source]  #scales::alpha(colors[y_source], 0.2)
    }
```

```r
    if (!is.null(background) & plot_idx %in% background) {
      color <- colors["grey"]
    }
    cat(paste(
      "source", y_source,
      "resolution", y_resolution,
      "preprocess", data_func,
      "r-squared", r_squared(xy),
      # Calculate a linear model to see if it is very different... it isn't.
      "fitr2", summary(lm(xy$y~xy$x))$adj.r.squared,
      "\n"
    ))

    if (coarsest_r2$res == "none" || y_resolution == "coarse") {
      coarsest_r2$res <- y_resolution
      coarsest_r2$value <- r_squared(xy)
    }
    points(xy, col = color, cex = cex, pch = pch)
  }
  if (rsquared > 0) {
    percent <- sprintf("%2.0f%%", 100 * coarsest_r2$value)
    text(rsquared, y=2, labels = substitute(R^2 == pc, list(pc=percent)), adj=0, cex=1.5)
  }
}
```

Given the functions, now create a plot with everything together. It looks crowded with all of the datasets in place.

```r
density_label <- c("BIMEP Population Density", "Mapped Population Density")
maximum1k <- 1.01 * max(density_from_source_resolution("LandScan", "coarse")$y, na.rm = TRU

scatter1k_together <- function() {
  maximum1k <- 1.01 * max(density_from_source_resolution("LandScan", "coarse")$y, na.rm =
  subjects <- c("HRSL", "LandScan", "WorldPop-C", "WorldPop-U",
                "GPW", "HRSL", "WorldPop-C", "WorldPop-U")
  uniq_cnt <- length(unique(subjects))
  resolution <- c(rep("coarse", uniq_cnt), rep("fine", length(subjects) - uniq_cnt))
  pop_scatter(subjects, resolution, density_label, "A", maximum1k)
  uniq <- unique(subjects)
  legend("topleft", legend =  uniq, col = colors[uniq],
            lty = 1, lwd = 2, cex = 1, bg = "white", bty = "n")
}
scatter1k_together()
```

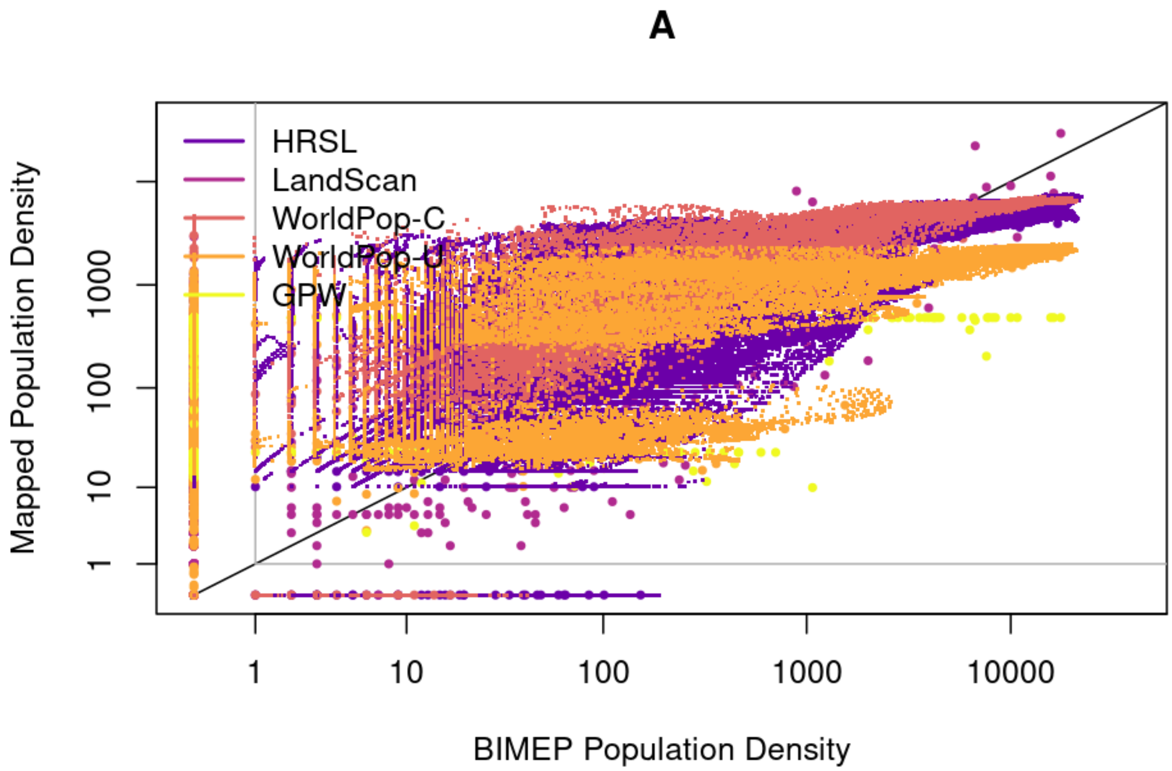


```
## source HRSL resolution coarse preprocess count r-squared 0.701374498047521 fitr2 0.7087
## source LandScan resolution coarse preprocess count r-squared 0.509956969635 fitr2 0.594
## source WorldPop-C resolution coarse preprocess count r-squared 0.620161015429768 fitr2 (
## source WorldPop-U resolution coarse preprocess count r-squared -0.0620970152604292 fitr
## source GPW resolution coarse preprocess count r-squared -0.10559825133495 fitr2 0.07064
## source HRSL resolution fine preprocess density r-squared 0.727402046600685 fitr2 0.7333
## source WorldPop-C resolution fine preprocess density r-squared 0.636287259992594 fitr2 (
## source WorldPop-U resolution fine preprocess density r-squared -0.0710896310204641 fitr
```

In case it isn't shown, the R^2 values are these, where the second value is the finer resolution:

```
plot name HRSL plot index 1 r-squared 0.699043441824052
plot name LandScan plot index 2 r-squared 0.496735170243049
plot name WorldPop-C plot index 3 r-squared 0.620247080322208
plot name WorldPop-U plot index 4 r-squared -0.0646295272850235
plot name GPW plot index 5 r-squared -0.106461272679354
plot name HRSL plot index 6 r-squared 0.727402046600685
plot name WorldPop-C plot index 7 r-squared 0.636287259992594
plot name WorldPop-U plot index 8 r-squared -0.0710896310204641
```

```
scatter1k_ls <- function() pop_scatter(
  "LandScan", "coarse",
  density_label, "A) LandScan 1km", maximum1k)
scatter1k_ls()
```

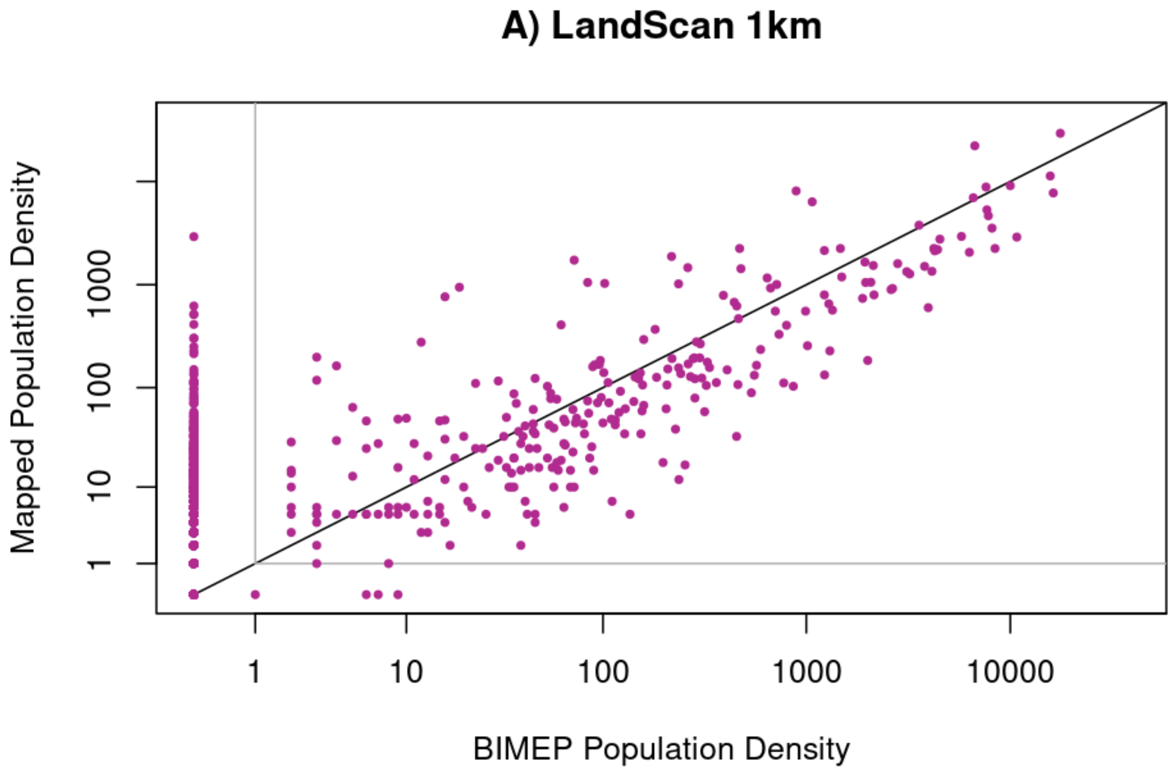


```
## source LandScan resolution coarse preprocess count r-squared 0.509956969635 fitr2 0.594[illegible]
```

```r
scatter1k_hrsl <- function() pop_scatter(
  rep("HRSL", 2), c("fine", "coarse"),
  density_label, "B) HRSL 1km", maximum1k, background = 1)
scatter1k_hrsl()
```

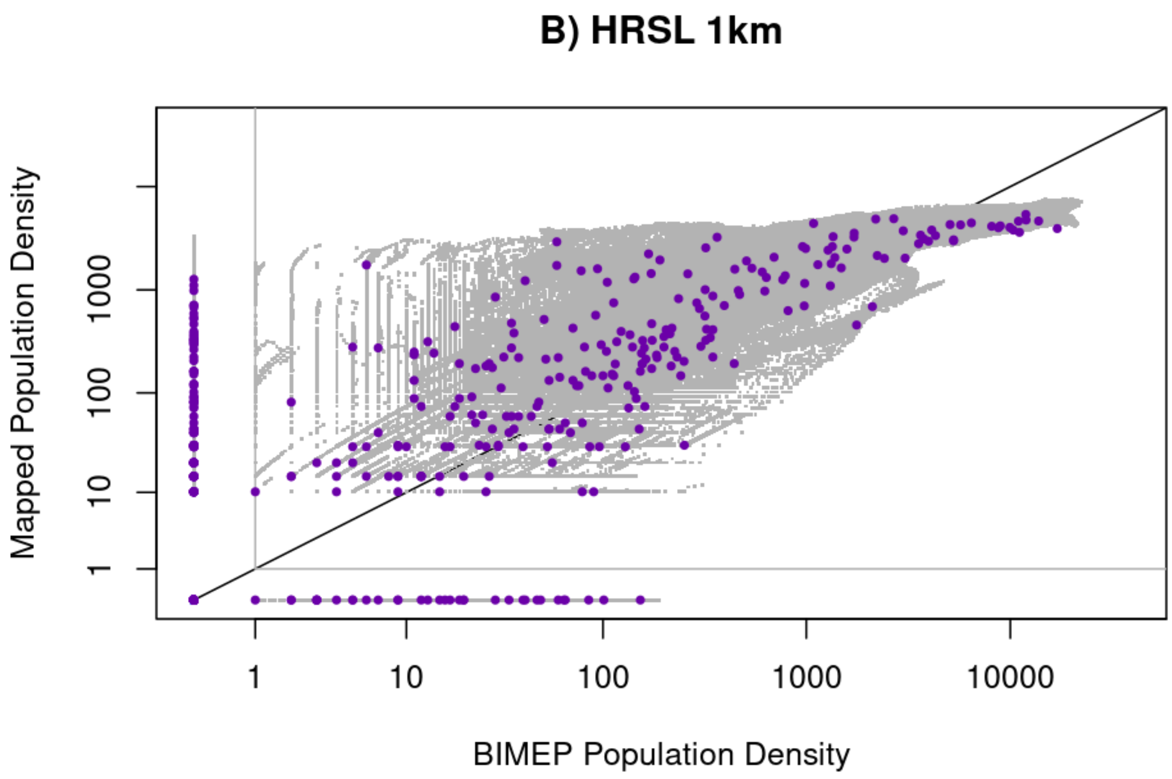


```
## source HRSL resolution fine preprocess density r-squared 0.727402046600685 fitr2 0.7333
## source HRSL resolution coarse preprocess count r-squared 0.701374498047521 fitr2 0.7087
```

```
scatter1k_wpc <- function() pop_scatter(
  rep("WorldPop-C", 2), c("fine", "coarse"),
  density_label, "C) WorldPop-C 1km", maximum1k, background = 1)
scatter1k_wpc()
```

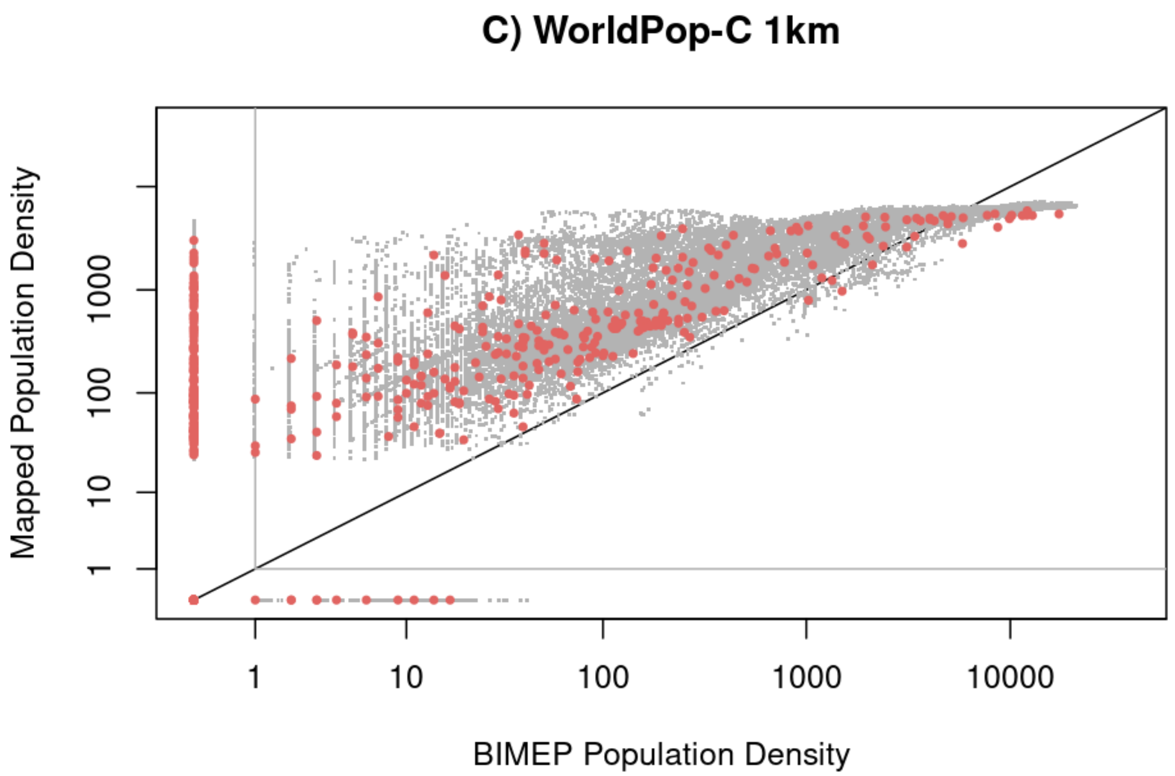


```
## source WorldPop-C resolution fine preprocess density r-squared 0.636287259992594 fitr2 (
## source WorldPop-C resolution coarse preprocess count r-squared 0.620161015429768 fitr2 (
```

```r
scatter1k_wpu <- function() pop_scatter(
  rep("WorldPop-U", 2), c("fine", "coarse"),
  density_label, "D) WorldPop-U 1km", maximum1k, background = 1)
scatter1k_wpu()
```

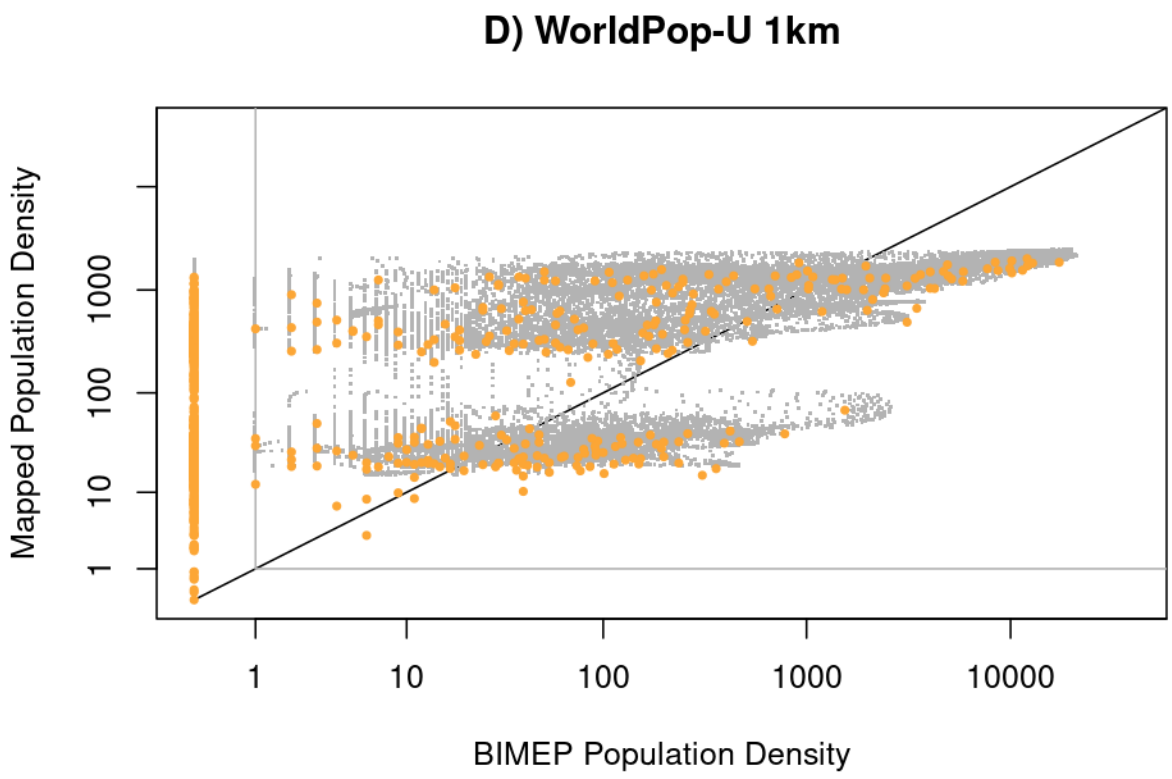


```
## source WorldPop-U resolution fine preprocess density r-squared -0.0710896310204641 fitr2
## source WorldPop-U resolution coarse preprocess count r-squared -0.0620970152604292 fitr2
```

```
scatter1k_gpw <- function() pop_scatter(
  rep("GPW", 1), c("coarse"),
  density_label, "E) GPW 1km", maximum1k)
scatter1k_gpw()
```

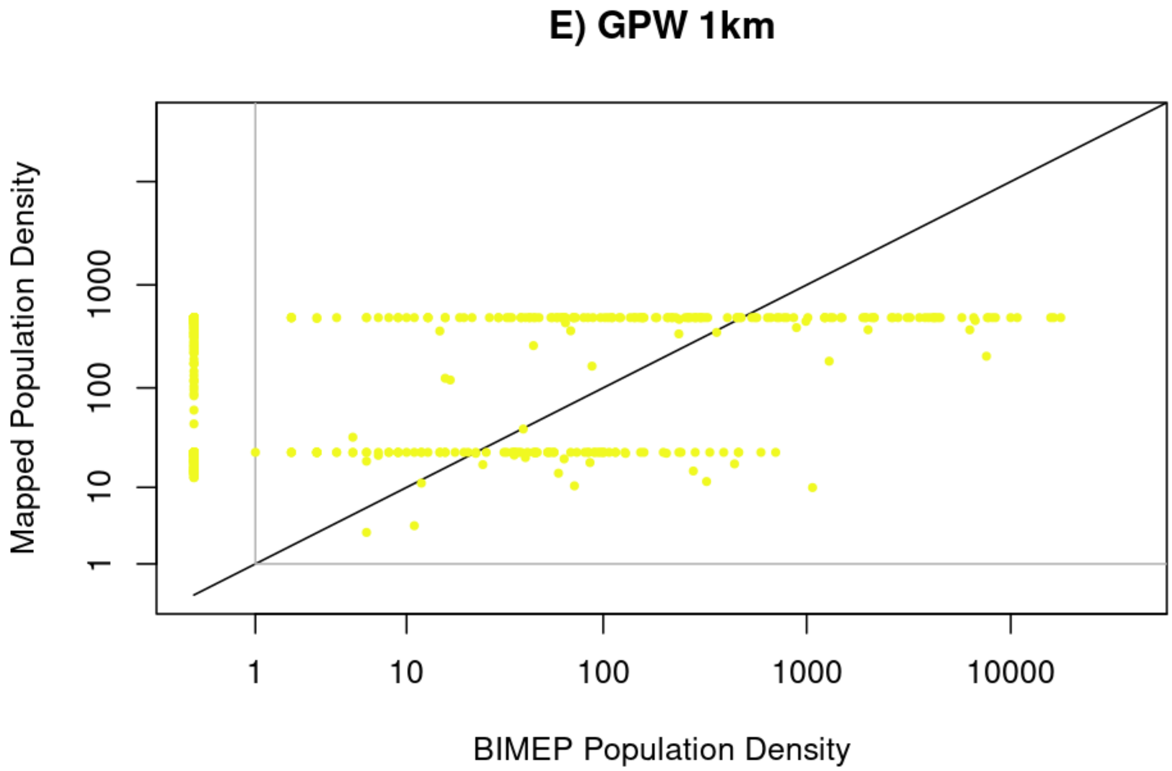


```
## source GPW resolution coarse preprocess count r-squared -0.10559825133495 fitr2 0.07064[illegible]
```

```
maximum100 <- 6.2
pop_scatter_label <- c("BIMEP", "Population Size")
scatter1k_hrsl_count <- function() pop_scatter(
  rep("HRSL", 1), c("fine"),
  pop_scatter_label, "F) HRSL 30x30 m", maximum100, counts = TRUE)
scatter1k_hrsl_count()
```

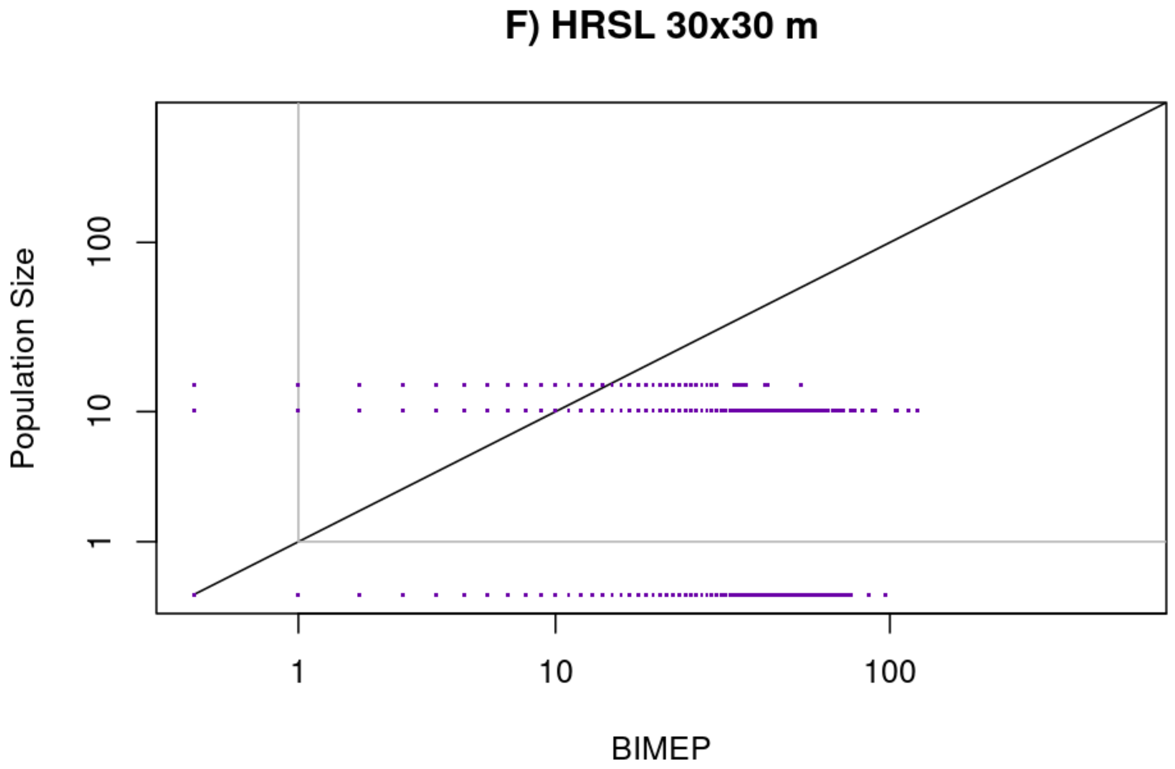


```
## source HRSL resolution fine preprocess count r-squared -0.0375299671679765 fitr2 0.1914
```

```
maximum100 <- 6.2
pop_scatter_label <- c("BIMEP", "Population Size")
scatter1k_wpc_count <- function() pop_scatter(
  rep("WorldPop-C", 1), c("fine"),
  pop_scatter_label, "G) WP-C 100x100 m", maximum100, counts = TRUE)
scatter1k_wpc_count()
```

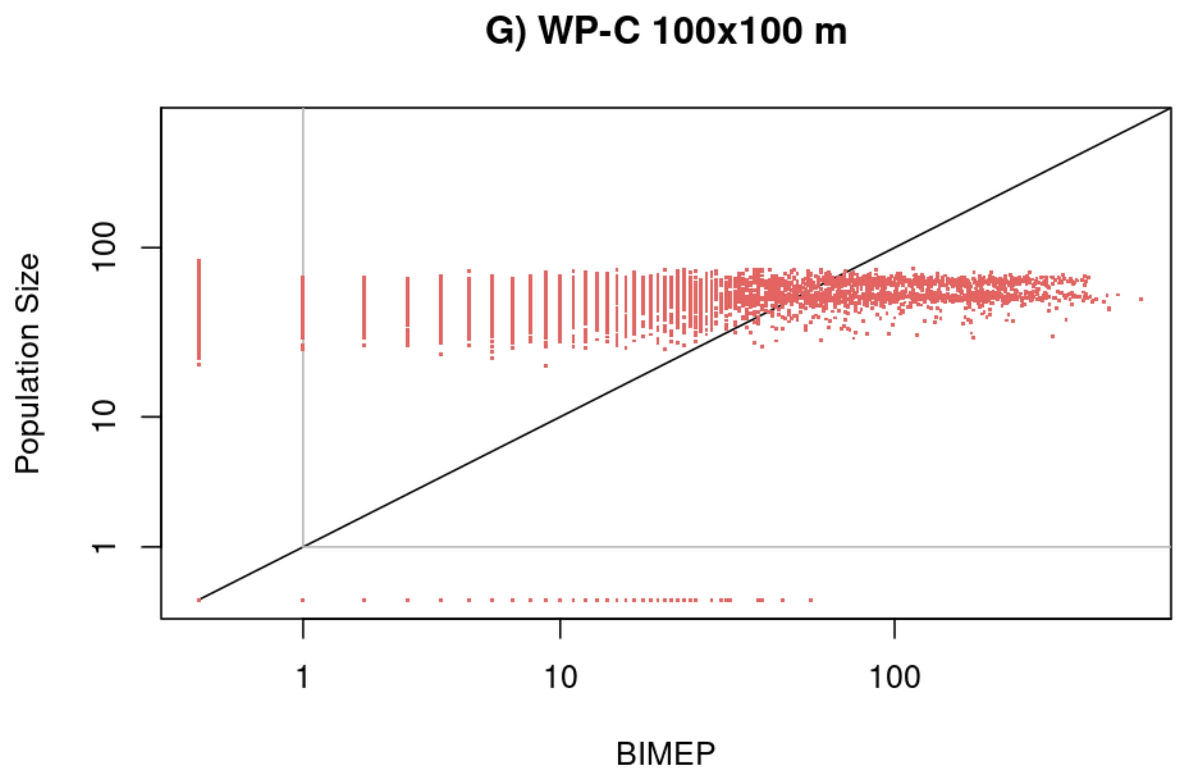


```
## source WorldPop-C resolution fine preprocess count r-squared 0.48156278277266 fitr2 0.48
```

```
maximum100 <- 6.2
pop_scatter_label <- c("BIMEP", "Population Size")
scatter1k_wpu_count <- function() pop_scatter(
  rep("WorldPop-U", 1), c("fine"),
  pop_scatter_label, "H) WP-U, 100x100 m", maximum100, counts = TRUE)
scatter1k_wpu_count()
```

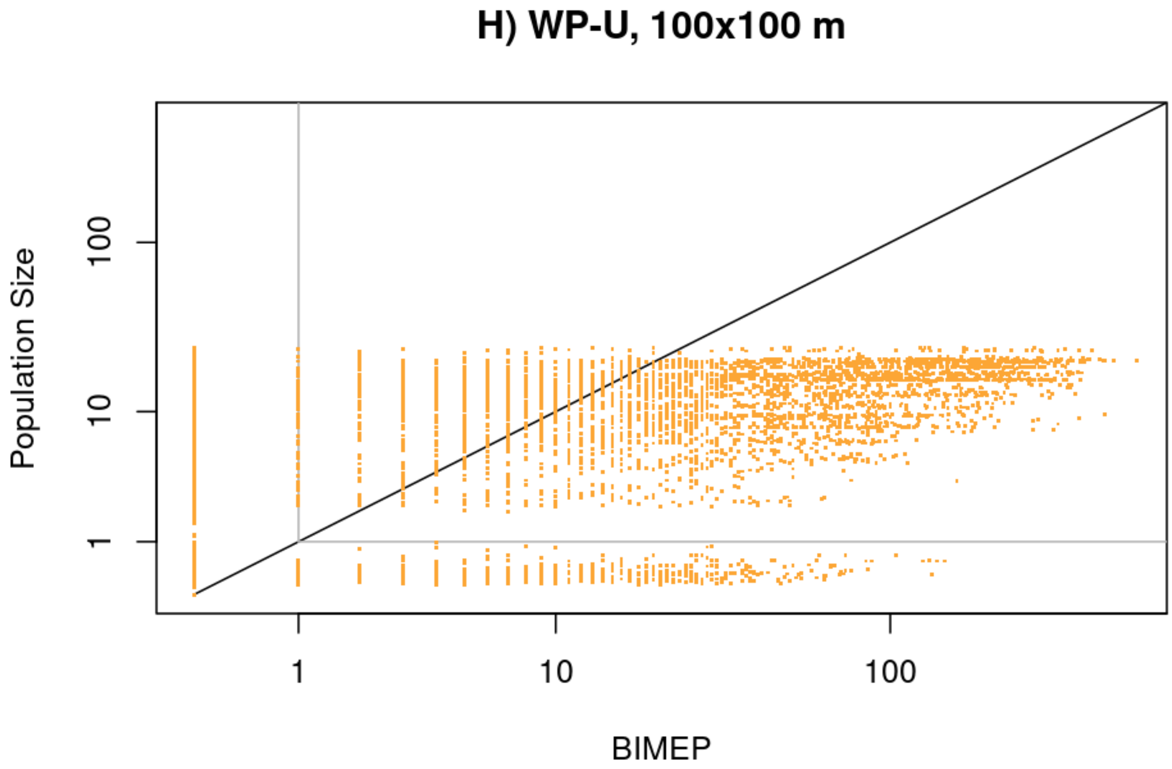


```
## source WorldPop-U resolution fine preprocess count r-squared -0.0166007450175778 fitr2 (
```

This makes the figure for the paper from those above.

```
make_figure <- function() {
  #par(mfrow = c(5, 2), mar = c(5, 4.2, 3, 2))
  par(mfrow = c(4, 2), mar = c(4, 3, 3, 2))
  # scatter1k_together()
  scatter1k_ls()
  scatter1k_hrsl()
  scatter1k_wpc()
  scatter1k_wpu()
  scatter1k_gpw()
  scatter1k_hrsl_count()
  scatter1k_wpc_count()
  scatter1k_wpu_count()
}
popbioko::save_plot(make_figure, "scatter1k")
```

```
## source LandScan resolution coarse preprocess count r-squared 0.509956969635 fitr2 0.594

## source HRSL resolution fine preprocess density r-squared 0.727402046600685 fitr2 0.7333
## source HRSL resolution coarse preprocess count r-squared 0.701374498047521 fitr2 0.7087

## source WorldPop-C resolution fine preprocess density r-squared 0.636287259992594 fitr2 (
## source WorldPop-C resolution coarse preprocess count r-squared 0.620161015429768 fitr2 (

## source WorldPop-U resolution fine preprocess density r-squared -0.0710896310204641 fitr
## source WorldPop-U resolution coarse preprocess count r-squared -0.0620970152604292 fitr
```

```
## source GPW resolution coarse preprocess count r-squared -0.10559825133495 fitr2 0.07064

## source HRSL resolution fine preprocess count r-squared -0.0375299671679765 fitr2 0.1914

## source WorldPop-C resolution fine preprocess count r-squared 0.48156278277266 fitr2 0.4

## source WorldPop-U resolution fine preprocess count r-squared -0.0166007450175778 fitr2

## source LandScan resolution coarse preprocess count r-squared 0.509956969635 fitr2 0.594

## source HRSL resolution fine preprocess density r-squared 0.727402046600685 fitr2 0.7333
## source HRSL resolution coarse preprocess count r-squared 0.701374498047521 fitr2 0.7087

## source WorldPop-C resolution fine preprocess density r-squared 0.636287259992594 fitr2
## source WorldPop-C resolution coarse preprocess count r-squared 0.620161015429768 fitr2

## source WorldPop-U resolution fine preprocess density r-squared -0.0710896310204641 fitr
## source WorldPop-U resolution coarse preprocess count r-squared -0.0620970152604292 fitr

## source GPW resolution coarse preprocess count r-squared -0.10559825133495 fitr2 0.07064

## source HRSL resolution fine preprocess count r-squared -0.0375299671679765 fitr2 0.1914

## source WorldPop-C resolution fine preprocess count r-squared 0.48156278277266 fitr2 0.4

## source WorldPop-U resolution fine preprocess count r-squared -0.0166007450175778 fitr2

## source LandScan resolution coarse preprocess count r-squared 0.509956969635 fitr2 0.594

## source HRSL resolution fine preprocess density r-squared 0.727402046600685 fitr2 0.7333
## source HRSL resolution coarse preprocess count r-squared 0.701374498047521 fitr2 0.7087

## source WorldPop-C resolution fine preprocess density r-squared 0.636287259992594 fitr2
## source WorldPop-C resolution coarse preprocess count r-squared 0.620161015429768 fitr2

## source WorldPop-U resolution fine preprocess density r-squared -0.0710896310204641 fitr
## source WorldPop-U resolution coarse preprocess count r-squared -0.0620970152604292 fitr

## source GPW resolution coarse preprocess count r-squared -0.10559825133495 fitr2 0.07064

## source HRSL resolution fine preprocess count r-squared -0.0375299671679765 fitr2 0.1914

## source WorldPop-C resolution fine preprocess count r-squared 0.48156278277266 fitr2 0.4

## source WorldPop-U resolution fine preprocess count r-squared -0.0166007450175778 fitr2
```

```r
make_figure()
```

```
## source LandScan resolution coarse preprocess count r-squared 0.509956969635 fitr2 0.594

## source HRSL resolution fine preprocess density r-squared 0.727402046600685 fitr2 0.7333
## source HRSL resolution coarse preprocess count r-squared 0.701374498047521 fitr2 0.7087

## source WorldPop-C resolution fine preprocess density r-squared 0.636287259992594 fitr2
## source WorldPop-C resolution coarse preprocess count r-squared 0.620161015429768 fitr2

## source WorldPop-U resolution fine preprocess density r-squared -0.0710896310204641 fitr
## source WorldPop-U resolution coarse preprocess count r-squared -0.0620970152604292 fitr

## source GPW resolution coarse preprocess count r-squared -0.10559825133495 fitr2 0.07064

## source HRSL resolution fine preprocess count r-squared -0.0375299671679765 fitr2 0.1914

## source WorldPop-C resolution fine preprocess count r-squared 0.48156278277266 fitr2 0.4
```

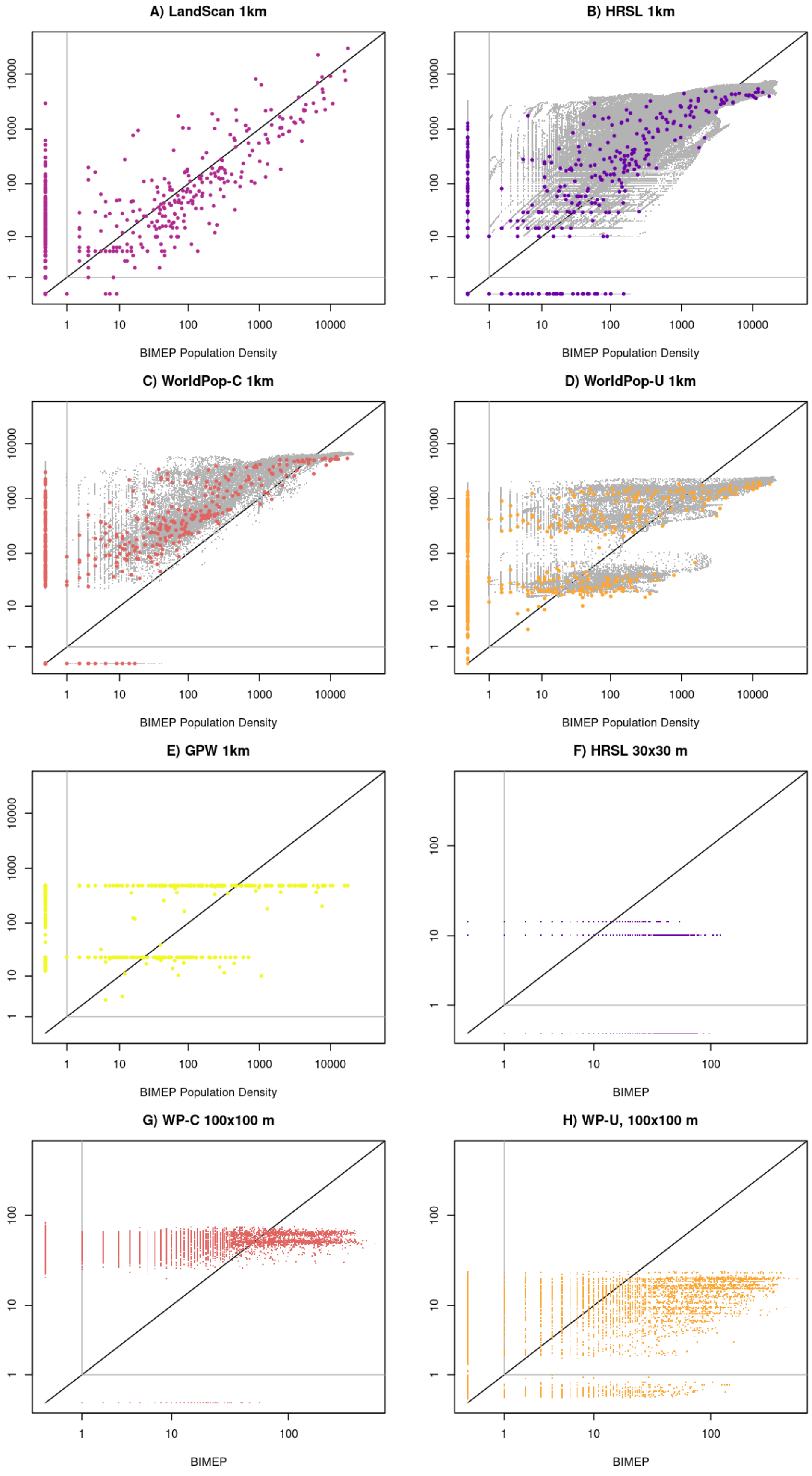
A) LandScan 1km
B) HRSL 1km
C) WorldPop-C 1km
D) WorldPop-U 1km
E) GPW 1km
F) HRSL 30x30 m
G) WP-C 100x100 m
H) WP-U, 100x100 m
BIMEP Population Density
BIMEP
1
10
100
1000
10000

```
## source WorldPop-U resolution fine preprocess count r-squared -0.0166007450175778 fitr2 (
```

5 Cumulative Maps

This section builds the cumulative maps in figure 5. It separates the work into one part to generate x and y data and one part to decide axes and such for that data.

```
linear_from_source_resolution <- function(source, resolution, map_list) {
  grid <- unique(files_df[files_df$source == source, "grid"])
  xy_df <- files_df[files_df$resolution == resolution & files_df$grid == grid, ]
  x_map_name <- xy_df[xy_df$source == "BIMEP", "name"]
  y_map_name <- xy_df[xy_df$source == source, "name"]
  x <- raster::getValues(map_list[[x_map_name]])
  y <- raster::getValues(map_list[[y_map_name]])
  list(x = x, y = y)
}

sources <- c("BIMEP", "HRSL", "LandScan", "WorldPop-C", "WorldPop-U", "GPW")

# This draws each line for the plots, taking care of color and line type.
lines_for_datasets <- function(datasets, linewidth) {
  for (data_idx in 1:length(datasets)) {
    lines(
      datasets[[data_idx]][["x"]],
      datasets[[data_idx]][["y"]],
      type = "l",
      col = colors[datasets[[data_idx]][["source"]]],
      lwd = linewidth,
      lty = ifelse(datasets[[data_idx]][["resolution"]] == "coarse", 1, 2)
      )
  }
}
```

```
cdfAreaLines <- function(H, source, resolution, linewidth = 2) {
  H <- sort(H)
  area <-  c(1:length(H)) / length(H)
  lines(
    area,
    cumsum(H) / sum(H),
    col = colors[source],
    lwd = linewidth,
    lty = ifelse(resolution == "coarse", 1, 2)
    )
}

cdfAreaFigure <- function(llwd = 1, label = "") {
  plot(
    vector(mode = "numeric", length = 0),
    xlim = c(0, 1), ylim = c(0, 1),
    xlab =  "% Area", ylab =  "Proportion of Population",
    main = label
    )
  sources <- c("BIMEP", "HRSL", "LandScan", "WorldPop-C", "WorldPop-U", "GPW")
  for (source in sources) {
```

```
    for (resolution in c("coarse", "fine")) {
      if (source == "BIMEP") {
        data <- linear_from_source_resolution("HRSL", resolution, density)$x
      } else {
        data <- linear_from_source_resolution(source, resolution, density)$y
      }
      cdfAreaLines(data, source, resolution, linewidth = llwd)
    }
  }
  legend("topleft", legend =  sources, col = colors[sources],
         lty = 1, lwd = 2, cex = 1)
}

cdfAreaFigure(2)
```

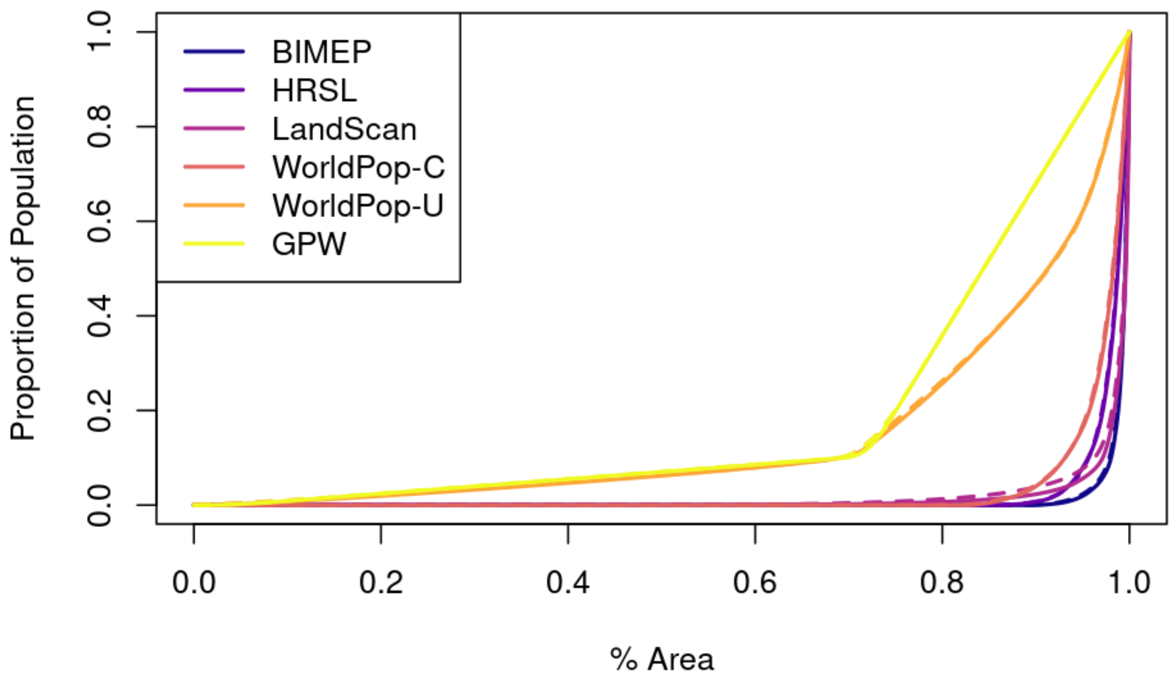


```
cdfDens <- function(H, log_base) {
  H <- sort(H)
  mx <- ceiling(log(max(H), base = log_base))

  c2 <- 1:mx
  ixz <- which(H == 0)
  Pz <- length(ixz)
  for (i in c2) {
    ix <- which(H < log_base^i)
    Pz <- c(Pz, sum(H[ix]))
  }
```

```
  pop <- c(0, log_base^c2)
  cbind(pop, Pz)
}


logPopAreaLines <- function(H, source, resolution, log_base, linewidth = 2) {
  dd <- cdfDens(H,log_base)[-1,]
  P <- dd[,1]
  H <- dd[,2]
  l <- log10(max(H))

  lines(
    P,
    H / max(H),
    col = colors[source],
    lwd = linewidth,
    lty = ifelse(resolution == "coarse", 1, 2)
    )
}


logPopAreaFigure <- function(llwd = 1, log_base = 2, label = "") {
  plot(
    vector(mode = "numeric", length = 0),
    log = "x",
    xlim = c(1.05, 0.5 * 10^5),
    ylim = c(0, 1),
    xlab =  "log(Pop Dens)",
    ylab =  "Proportion of Population",
    xaxt = "n",
    main = label
    )

  log_max <- 0
  for (source in sources_ordered) {
    for (resolution in c("coarse", "fine")) {
      if (source == "BIMEP") {
        data <- linear_from_source_resolution("HRSL", resolution, density)$x
      } else {
        data <- linear_from_source_resolution(source, resolution, density)$y
      }
      log_max <- max(log_max, log10(max(data, na.rm = TRUE)))
      logPopAreaLines(data, source, resolution, log_base, linewidth = llwd)
    }
  }
  axis(1, 10^(0:(log_max + 1)), logarithmic_axis_labels[1:(log_max + 2)])
  legend("topleft", legend =  sources, col = colors[sources],
         lty = 1, lwd = 2, cex = 1)
}

logPopAreaFigure(2)
```

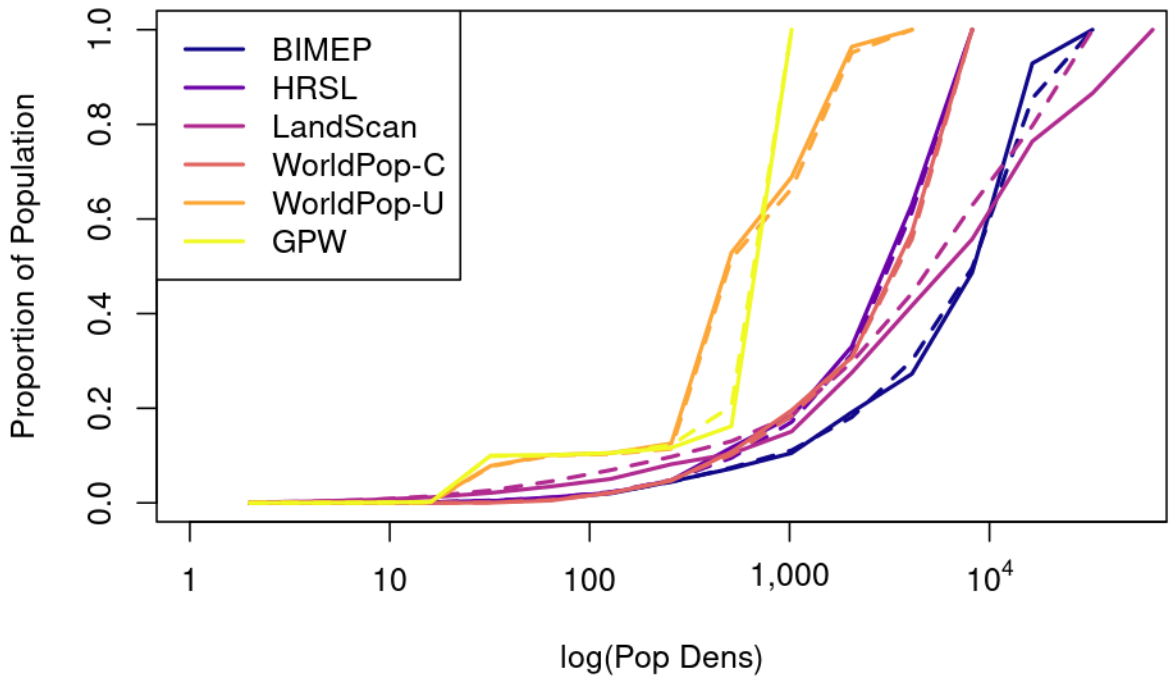

Now the third part of Figure 5, a density plot that is just density, not cumulative.

```
log_density <- function(H, base_power = 2) {
  H <- H[!is.na(H)]
  c2 <- ceiling(max(log(H[H > 0], base = base_power)))
  ixz <- which(H <= 0)
  upper <- 0
  if (length(ixz) > 0) {
    HH <- H[-ixz]
  } else {
    HH <- H
  }
  Pz <- length(ixz)
  for (i in 1:c2) {
    upper <- c(upper, base_power^(i - 1))
    ix <- which(HH < base_power^(i - 1))
    if (length(ix) > 0) {
      Pz <- c(Pz, sum(HH[ix]))
      HH <- HH[-ix]
    } else {
      Pz <- c(Pz, 0)
    }
  }
  while (Pz[length(Pz)] == 0) {
    Pz <- Pz[-length(Pz)]
    upper <- upper[-length(Pz)]
  }
```

```r
  cbind(round(Pz), upper)
}

single_test <- linear_from_source_resolution("LandScan", "coarse", density)$y
dens_test <- log_density(single_test, base_power = 1.2)


density_kernel_data <- function(base_power, offset = 0) {
  datasets <- vector(mode = "list", length = 2 * length(sources))
  running_idx <- 1
  for (source in sources_ordered) {
    for (resolution in c("coarse", "fine")) {
      if (source == "BIMEP") {
        data <- linear_from_source_resolution("HRSL", resolution, density)$x
      } else {
        data <- linear_from_source_resolution(source, resolution, density)$y
      }
      D <- log_density(data, base_power)[-1, 1]
      l <- length(D)
      x <- base_power^c(c(1:(l + 1))) * base_power^offset
      y <- c(D / sum(data, na.rm = TRUE), 0)
      datasets[[running_idx]] <- list(source = source, resolution = resolution, x = x, y =
      running_idx <- running_idx + 1
    }
  }
  datasets
}


density_kernel_figure <- function(base_power, linewidth = 1, label = "") {
  datasets <- density_kernel_data(base_power)
  l = max(sapply(datasets, function(ds) ds$l))
  plot(
    vector(mode = "numeric", length = 0),
    xaxt = "n",
    log = "x",
    xlim = c(5, base_power^(l + 2)),
    ylim = c(0, 0.4),
    xlab =  "log(Pop Dens)", ylab =  "Proportion of Population",
    main = label
    )
  lines_for_datasets(datasets, linewidth)
  L <- 4
  axis(1, 10^(0:(L + 1)) + 0.5, logarithmic_axis_labels[1:(L + 2)])
  legend("topleft", legend =  sources, col = colors[sources],
         lty = 1, lwd = 2, cex = 1)
}

density_kernel_figure(1.2, 2, "C")
```

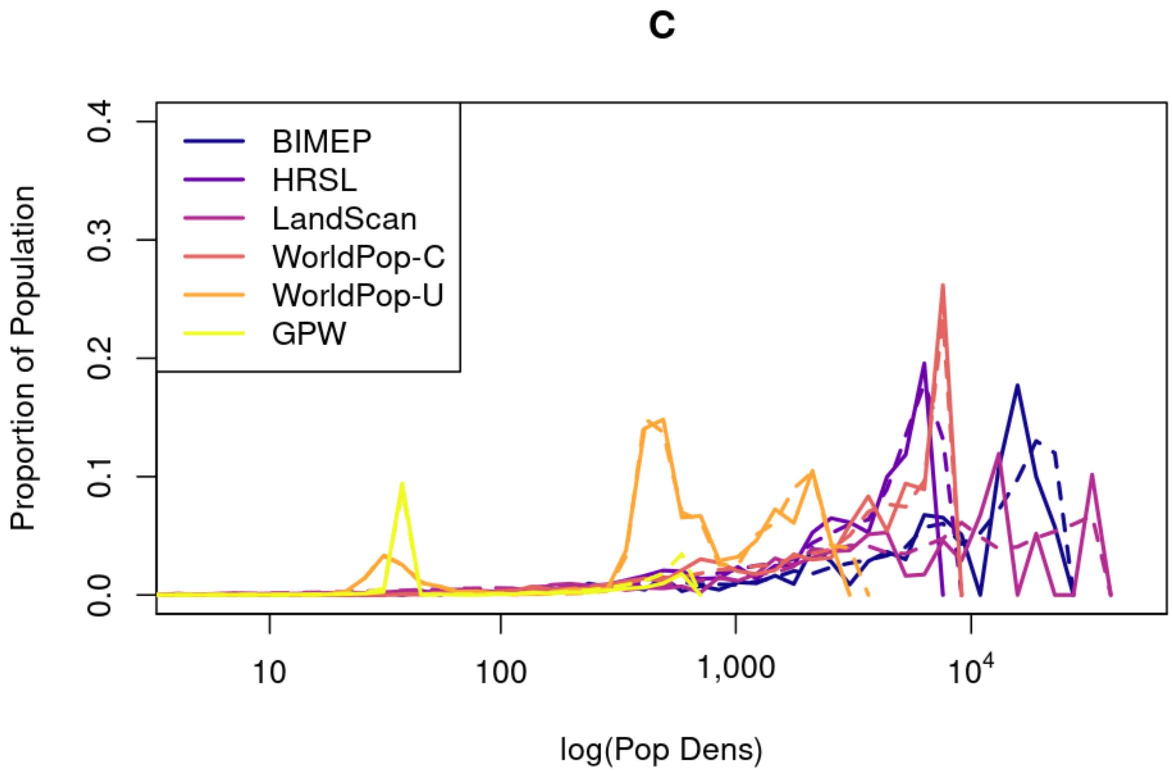


```
cdf_figure <- function() {
  par(mfrow = c(3, 1), mar = c(5, 5, 3, 2))
  cdfAreaFigure(2, label = "A")
  logPopAreaFigure(2, label = "B")
  density_kernel_figure(1.2, 2, "C")
}
save_plot(cdf_figure, "CDF")
cdf_figure()
```

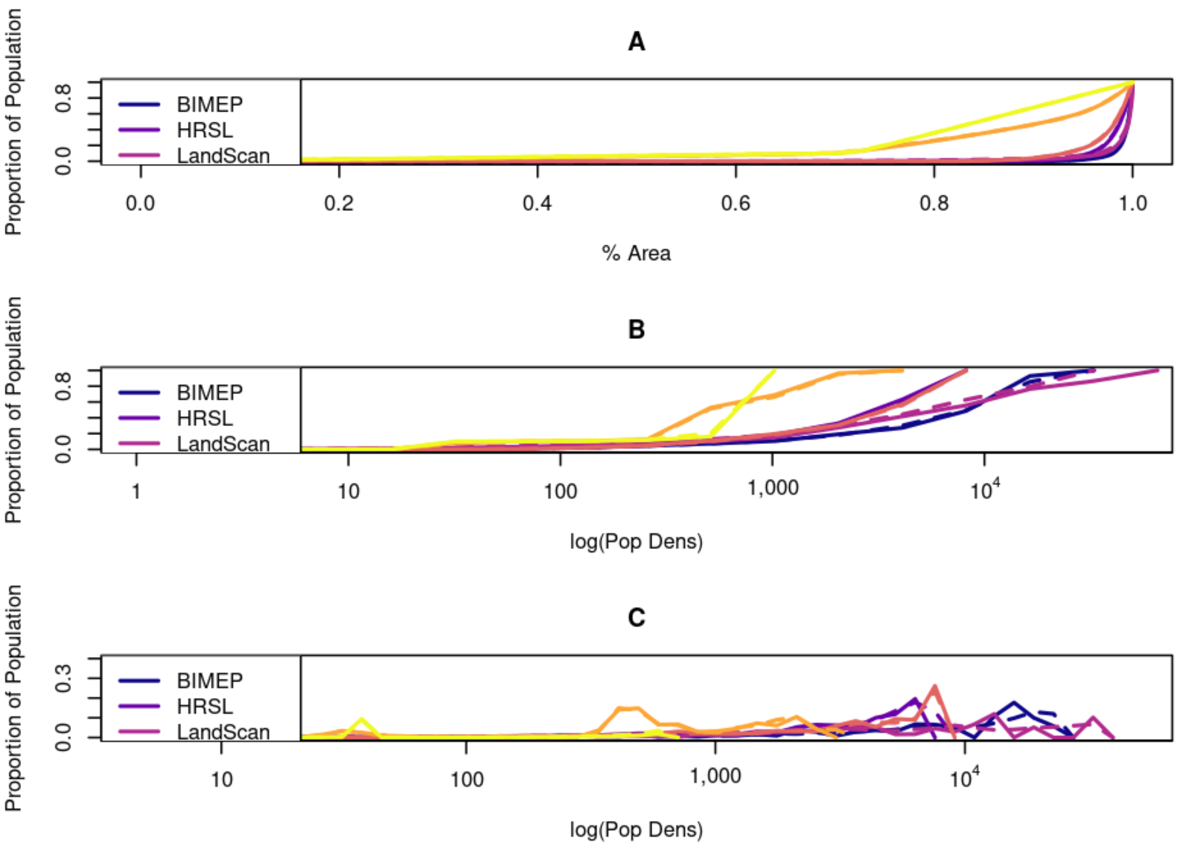

6 Proportion, Accuracy, Recall, and Precision

Figure 6 is proportion, accuracy, recall, and precision.

6.1 Define the values

These metrics are on truth or falsity, and it applies here when we assign cutoff values, in order to create categories. This section is our canonical definition for those categories.

```
breakpoints <- c(
  empty = 0,
  rural1 = 1,
  rural2 = 50,
  periurban = 250,
  urban = 1000
)
break_colors <- c(
  empty = grey(0.95),
  rural1 = "ivory",
  rural2 = "lavender",
  periurban = "cornsilk",
  urban = "aliceblue"
)
stopifnot(all(names(breakpoints) == names(break_colors)))
```

```
lessThanCat <- function(tau, map, gold, km2 = 1) {
  tau <- tau * km2
  accuracy_profile(truth_table(function(observed) {observed < tau}, gold, map))
}

greaterThanCat <- function(tau, map, gold, km2 = 1) {
  tau <- tau * km2
  accuracy_profile(truth_table(function(observed) {tau <= observed}, gold, map))
}

betweenCat <- function(L, U, map, gold, km2 = 1) {
  L <- L * km2
  U <- U * km2
  # Choose comparison so that it is cadlag.
  accuracy_profile(truth_table(function(obs) {L <= obs & obs < U }, gold, map))
}

fullCat <- function(map, gold, km2 = 1) {
  with(as.list(breakpoints), {
    data.frame(as.vector(rbind(
      empty = lessThanCat(rural1, map, gold, km2),
      rural1 = betweenCat(rural1, rural2, map, gold, km2),
      rural2 = betweenCat(rural2, periurban, map, gold, km2),
      periurban = betweenCat(periurban, urban, map, gold, km2),
      urban = greaterThanCat(urban, map, gold, km2)
    )))
  })
}
```

6.2 Proportion

```
landAA <- function(H, breakpoints, area = 1) {
  stopifnot(class(breakpoints) == "numeric")
  breakpoints <- c(breakpoints, Inf) / area

  Pz <- rep(0, length(breakpoints))
  for (cut_idx in 1:length(breakpoints)) {
    ixz <- which(H < breakpoints[cut_idx])
    Pz[cut_idx] <- length(ixz)
    if (Pz[cut_idx] > 0) {
      H <- H[-ixz]
    }  # Nothing to remove
  }
  Pz / sum(Pz)
}

over_arrays <- function(transform) {
  datasets <- vector(mode = "list", length = 2 * length(sources))
  running_idx <- 1
  for (source in sources) {
    for (resolution in c("coarse", "fine")) {
      if (source == "BIMEP") {
        data <- linear_from_source_resolution("HRSL", resolution, density)$x
```

```r
      } else {
        data <- linear_from_source_resolution(source, resolution, density)$y
      }
      datasets[[running_idx]] <- list(source = source, resolution = resolution, x = transfo
      running_idx <- running_idx + 1
    }
  }
  datasets
}

proportion_data <- function(breakpoints) {
  over_arrays(function(data) {
    landAA(data, breakpoints)
  })
}
example_data <- proportion_data(c(1, 50, 250, 1000))
```

```r
#' Make shaded backgrounds for all four figures.
#' @param splits The sides of the boxes, where colors change.
make_boxes <- function(splits) {
  stopifnot(length(splits) == length(break_colors) + 1)
  boxes <- vector(mode = "list", length = length(break_colors))
  for (color_idx in 1:length(break_colors)) {
    boxes[[color_idx]] <- list(splits[color_idx], splits[color_idx + 1], break_colors[colo
  }
  makebox <- function(x1, x2, color) {
    xx <- c(x1, x1, x2, x2)
    yy <- c(0,1,1,0)
    polygon(cbind(xx, yy), border = NA, col = color)
  }
  for (box_idx in 1:length(boxes)) do.call(makebox, boxes[[box_idx]])
}


pdfAreaFigure <- function(lb = "") {
  # Start from 2 because this function uses the upper cutoff.
  prop_data <- proportion_data(breakpoints[2:length(breakpoints)])
  plot(
    vector(mode = "numeric", length = 0),
    xlim = c(1, 7),
    ylim = c(0, 1),
    xaxt = "n",
    xlab = "",
    ylab = "Proportion",
    main = lb
    )
  axis(1, c(2, 2:5 + 0.5), c(0,1,50,250,1000))

  make_boxes(1:6 + 0.5)

  offset_idx <- 0
  y_data <- NULL
  for (find_source in sources_ordered) {
    for (data_idx in 1:length(prop_data)) {
```

```
      y_data <- with(prop_data[[data_idx]], {
        if ((source == find_source) & (resolution == "coarse")) {
          return(x)
        }
      })
      if (!is.null(y_data)) break
    }
    offset <- -.21 + 0.14 * offset_idx
    stopifnot(!is.null(y_data))
    lines(2:6 + offset, y_data, type = "h", lwd = 5, col = colors[find_source], lty = 1)
    offset_idx <- offset_idx + 1
  }

  legend("topright", legend = sources_ordered, col = colors[sources_ordered],
         lty = 1, lwd = 2, cex = 1, bg = "white")
  #mtext("A", side = 3, line = 0.2, outer = FALSE, cex = 1.6)
}

pdfAreaFigure()
```

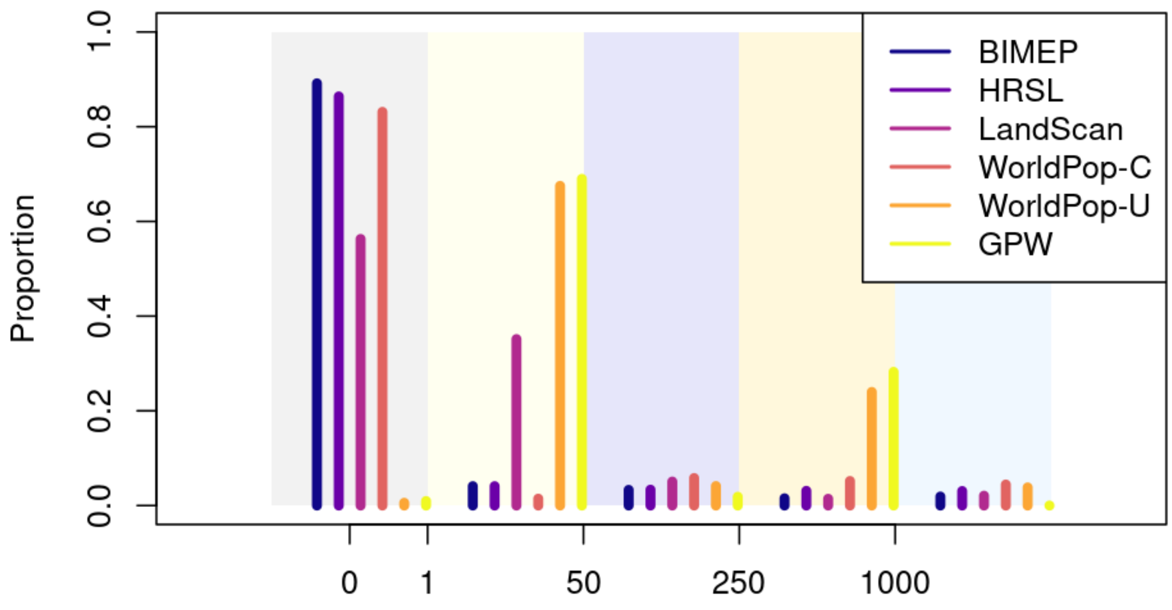

6.3 Precision

Precision is also known as positive predictive value, so it's the ppv variable.

```
get_measure <- function(x, map, gold, measure) {
  if (x == 0) {
```

```
    lessThanCat(x, map, gold)[[measure]]
  } else {
    greaterThanCat(x, map, gold)[[measure]]
  }
}
getPPV <- function(x, map, gold) get_measure(x, map, gold, "ppv")
```

```
compare_sources <- sources[!sources %in% "BIMEP"]
ppv_data <- sapply(compare_sources, function(x) {
  linear_from_source_resolution(x, "coarse", density)
})
dimnames(ppv_data) <- list(c("x", "y"), compare_sources)
mn <- 1
ppv_mx <- 0.7 * max(ppv_data["y", "LandScan"][[1]], na.rm = TRUE)
tk <- c(breakpoints, 4000, 10000)

xx <- c(0, seq(sqrt(mn), sqrt(ppv_mx), length.out = 100)^2)
xxp <- sqrt(xx)
tk0 <- sqrt(tk)

pw <- 1 / 3.5
xx <- c(0, seq(mn^pw, ppv_mx^pw, length.out = 100)^(1 / pw))
xxp <- xx^pw
tk0 <- tk^pw

ppv_splits <- c(breakpoints, ppv_mx)^pw

generate_accuracy_y <- function(xx, measure) {
  ppv_y <- matrix(0, nrow = length(xx), ncol = length(compare_sources))
  for (source_idx in 1:length(compare_sources)) {
    source <- compare_sources[source_idx]
    gold <- ppv_data["x", source][[1]]
    observed <- ppv_data["y", source][[1]]
    ppv_y[1:length(xx), source_idx] <- as.vector(unlist(sapply(
      xx,
      function(x, map, gold) get_measure(x, map, gold, measure),
      map = observed,
      gold = gold
      )))
  }
  colnames(ppv_y) <- compare_sources
  ppv_y
}
ppv_y <- generate_accuracy_y(xx, "ppv")
```

```
PPVProfile <- function(ppv_y, name, lb = "") {
  plot(
    vector(mode = "numeric", length = 0),
    xlim = c(0, max(xxp)),
    ylim = c(0, 1),
    xaxt = "n",
    xlab = "Population Density",
    ylab = name,
    main = lb
```

```
    )
  axis(1, tk0, tk)

  make_boxes(ppv_splits)

  for (col_idx in 1:ncol(ppv_y)) {
    source <- colnames(ppv_y)[col_idx]
    lines(xxp, as.numeric(ppv_y[, col_idx]), type = "s", col = colors[source], lwd = 2)
  }
}
PPVProfile(ppv_y, "Precision")
```

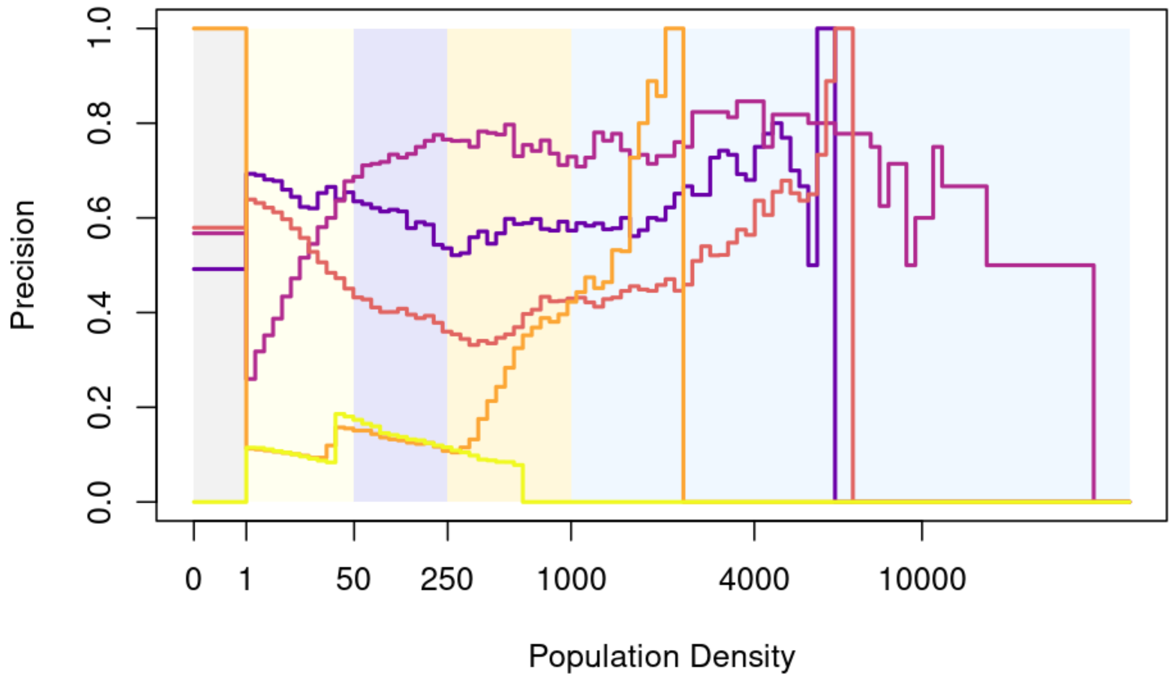

6.4 Accuracy

```
acc_y <- generate_accuracy_y(xx, "acc")
PPVProfile(acc_y, "Accuracy")
```

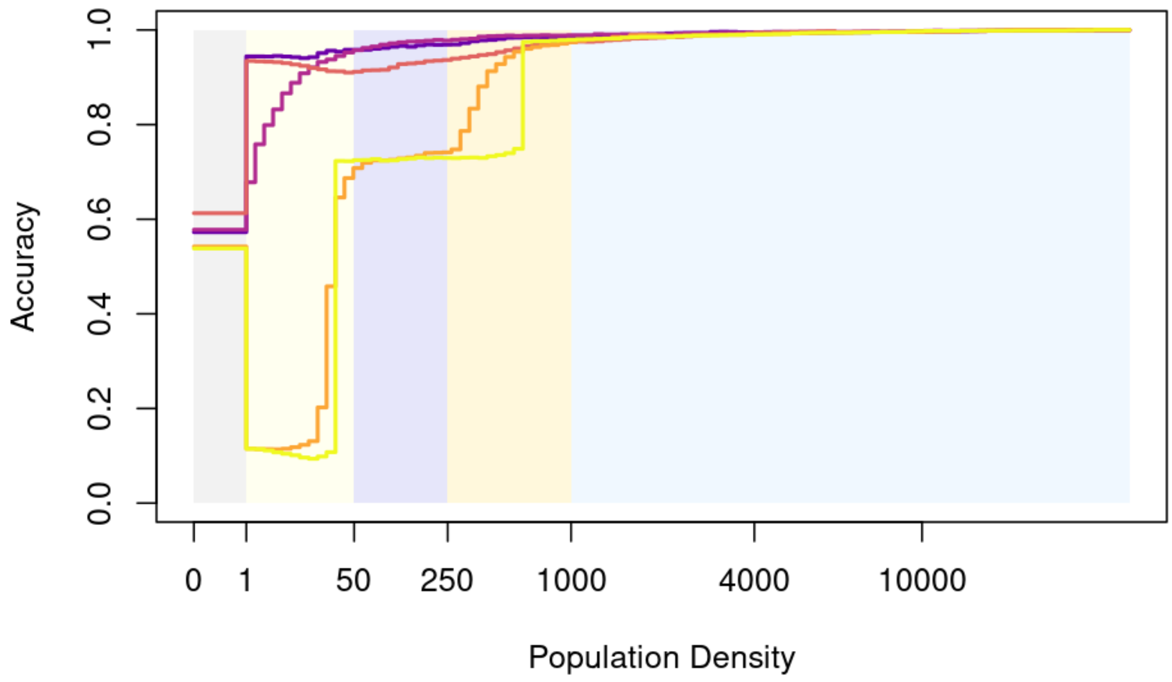

6.5 Recall

```
sens_y <- generate_accuracy_y(xx, "sens")
PPVProfile(sens_y, "Sensitivity")
```

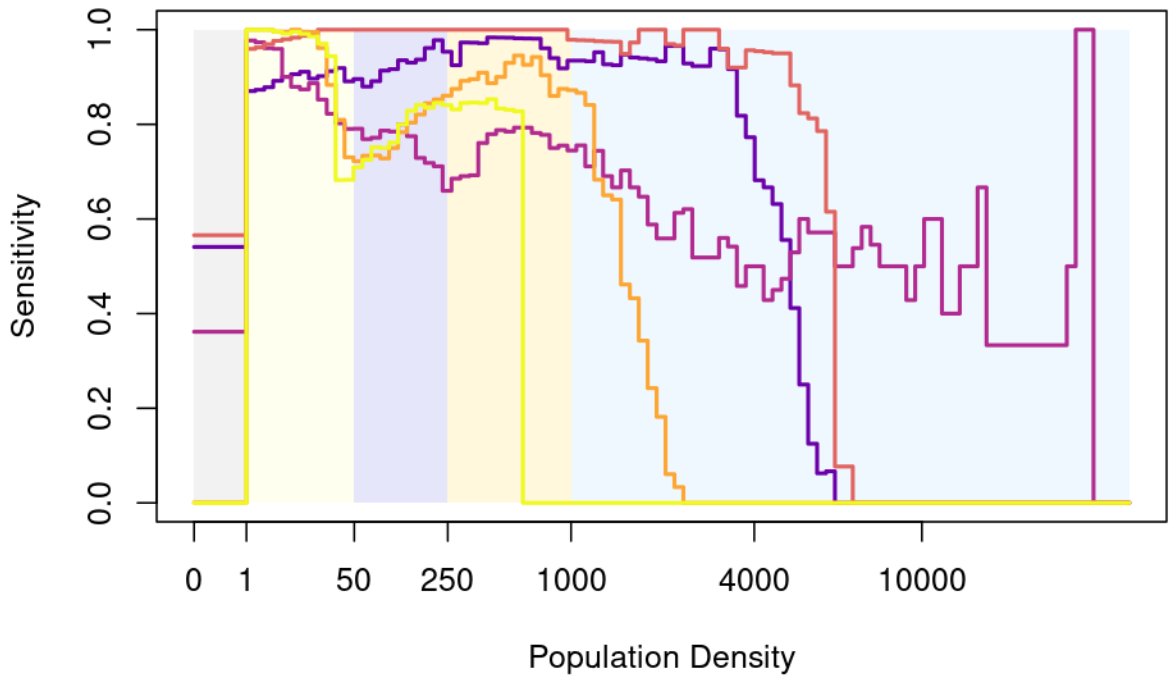

Last, assemble them for a figure.

```
area_figure <- function() {
  par(new, mfrow = c(2, 2), mar = c(4, 3, 2, 2))
  pdfAreaFigure(lb = "A")
  PPVProfile(acc_y, "Accuracy", lb = "B")
  PPVProfile(sens_y, "Sensitivity", lb = "C")
  PPVProfile(ppv_y, "Precision", lb = "D")
}
save_plot(area_figure, "AccuracyProfile")
area_figure()
```

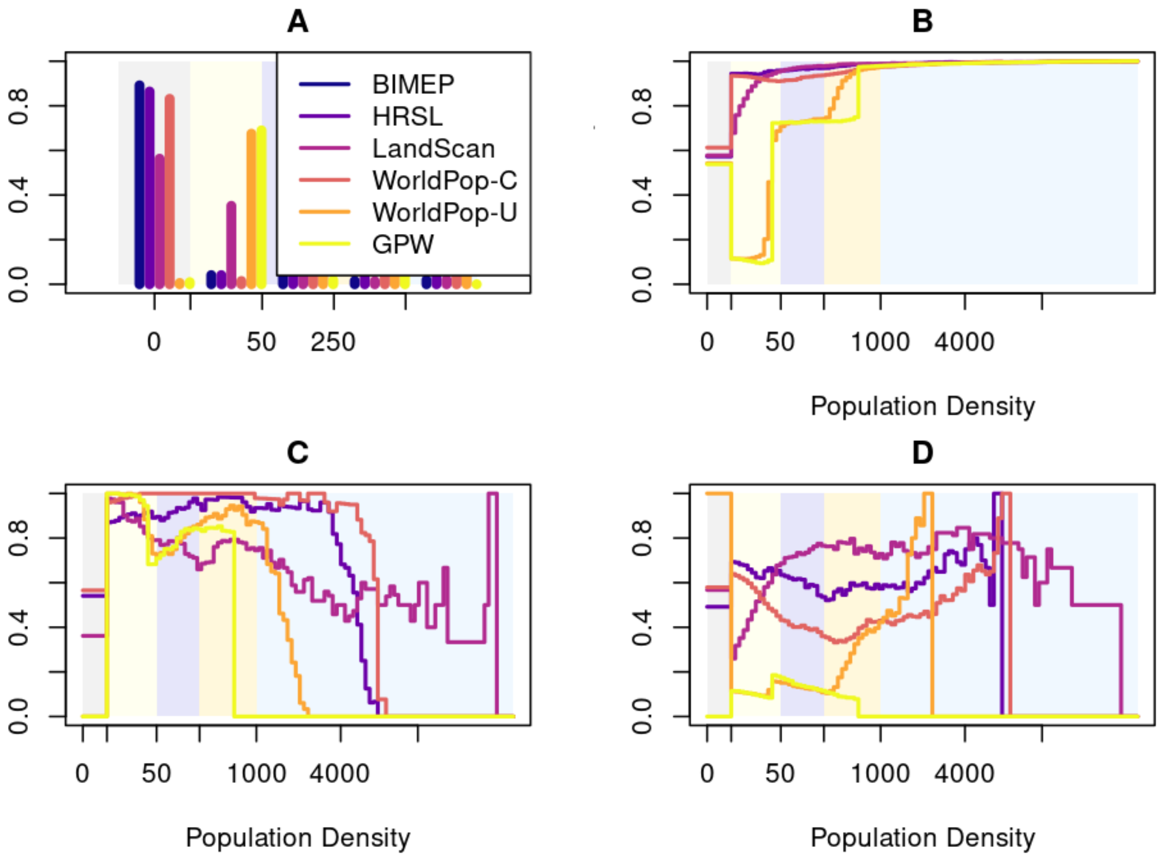

6.6 Table of ranges

This is table 3, which has accuracy, recall, and precision for ranges of values.

```
HRSL <- fullCat(ppv_data["y", "HRSL"][[1]], ppv_data["x", "HRSL"][[1]])
LS <- fullCat(ppv_data["y", "LandScan"][[1]], ppv_data["x", "LandScan"][[1]])
WP <- fullCat(ppv_data["y", "WorldPop-C"][[1]], ppv_data["x", "WorldPop-C"][[1]])
WPU <- fullCat(ppv_data["y", "WorldPop-U"][[1]], ppv_data["x", "WorldPop-U"][[1]])
GPW <- fullCat(ppv_data["y", "GPW"][[1]], ppv_data["x", "GPW"][[1]])

ppv <- cbind(HRSL = as.numeric(HRSL$ppv), LS = as.numeric(LS$ppv), WPC = as.numeric(WP$ppv)
sens <- cbind(HRSL = as.numeric(HRSL$sens), LS = as.numeric(LS$sens), WPC = as.numeric(WP$s
acc <- cbind(HRSL = as.numeric(HRSL$acc), LS = as.numeric(LS$acc), WPC = as.numeric(WP$acc)
accuracy_as_table <- signif(cbind(acc, sens, ppv), 3)
rownames(accuracy_as_table) <- rownames(HRSL)
t(accuracy_as_table)
```

```
##        empty rural1 rural2 periurban urban
## HRSL 0.9440 0.9480 0.9600    0.9670 0.986
## LS   0.6720 0.6740 0.9530    0.9750 0.989
## WPC  0.9340 0.9470 0.9170    0.9420 0.974
## WPU  0.1150 0.3260 0.9280    0.7550 0.976
## GPW  0.1150 0.3160 0.9570    0.7110 0.980
## HRSL 0.9530 0.3640 0.4000    0.4320 0.935
## LS   0.6320 0.7500 0.5380    0.2270 0.745
## WPC  0.9310 0.0818 0.1270    0.2780 0.978
```

```
## WPU  0.0028 0.5270 0.0380    0.3610 0.870
## GPW  0.0000 0.6200 0.0125    0.7270 0.000
## HRSL 0.9840 0.3640 0.3950    0.2130 0.581
## LS   0.9970 0.0869 0.3770    0.3030 0.729
## WPC  0.9950 0.2570 0.0714    0.0800 0.421
## WPU  1.0000 0.0356 0.0300    0.0223 0.430
## GPW  0.0000 0.0358 0.0500    0.0469 0.000
```

Remember that you can turn this into LaTex using the command `knitr::kable(t(accuracy_as_table), format = "latex")`.

7 Goodness of Fit Ratio

The goodness of fit ratio (GOFR) is both described in the paper and in the "Accuracy Metrics" notebook under the section SSD Profiles.

We need the admin2 for Bioko in order to recreate a table with GOFR for both the island and the four admin2 regions on the island. This admin-level shapefile is available by cropping the GADM data for Equatorial Guinea.

```
# The input map is in UTM, so we transform it to lat-long in order to agree
# with the raster coordinates of all rasters.
admin2_sf <- sf::st_transform(sf::st_read(rprojroot::find_package_root_file(
  "inst/extdata/admin2/bioko_admin2_fulldistricts.shp")),
  raster::crs(maps[1][[1]]))
admin2_st <- as(st_geometry(admin2_sf), "Spatial")
admin2_st_df <- as(admin2_sf[, "OBJECTID"], "Spatial")
tmap::tm_shape(admin2_sf) + tmap::tm_polygons("admin2")
```

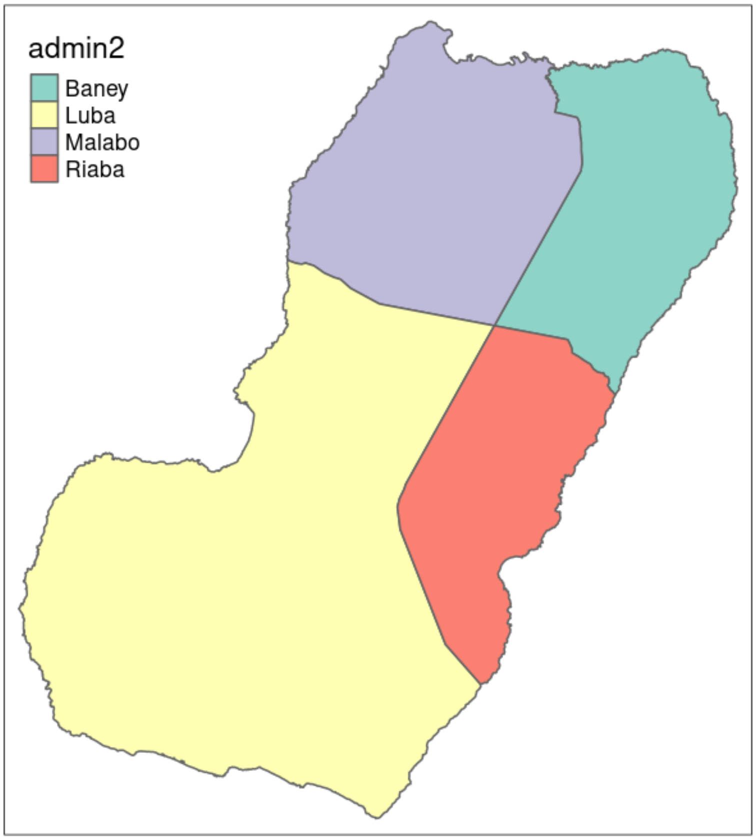

The GOFR itself is Sum-of-squared-errors divided by variance. We want to calculate this for the whole island and for districts on the island. Each GOFR calculation takes two maps as input, a source map, such as HRSL-fine, and a comparison map, which would be BIMEP-on-HRSL-fine-grid. So we will iterate through source maps, taking the source and the comparison map.

```
calculate_gofr <- function(normalize_gofr, gofr_map_type) {
# For each grid and resolution, there should be a map and a BIMEP version of that map.
admin_names <- c("Baney", "Malabo", "Luba", "Riaba")
# If these are out of order, then the names in admin_names are in the wrong order.
stopifnot(admin2_sf$OBJECTID == 1:4)
gofr <- files_df[files_df$source != "BIMEP", ]
rownames(gofr) <- paste(gofr$source, gofr$resolution)
gofr$GOFR <- numeric(nrow(gofr))
gofr$Baney <- numeric(nrow(gofr))
gofr$Malabo <- numeric(nrow(gofr))
gofr$Luba <- numeric(nrow(gofr))
gofr$Riaba <- numeric(nrow(gofr))

# This is the GOFR equation. This defines it.
gofr_of_maps_gold_variance <- function(pops_map, pops_bimep, normalize = FALSE) {
  if (normalize) {
    pops_bimep <- pops_bimep / sum(pops_bimep, na.rm = TRUE)
    pops_map <- pops_map / sum(pops_map, na.rm = TRUE)
  }
  pixel_cnt <- sum(is.finite(pops_map))
  # Ignore NA because they tell us where there is water. Rasters are designed to agree on
  pixel_error <- sum((pops_map - pops_bimep)^2, na.rm = TRUE) / pixel_cnt
```

```
  pixel_error / var(pops_bimep, na.rm = TRUE)
}

# This is the GOFR equation. This defines it.
gofr_of_maps <- function(pops_map, pops_bimep, normalize = FALSE) {
  if (normalize) {
    pops_bimep <- pops_bimep / sum(pops_bimep, na.rm = TRUE)
    pops_map <- pops_map / sum(pops_map, na.rm = TRUE)
  }
  pixel_cnt <- sum(is.finite(pops_map))
  # Ignore NA because they tell us where there is water. Rasters are designed to agree on w
  pixel_error <- sum((pops_map - pops_bimep)^2, na.rm = TRUE)
  pixel_error / sum((pops_map - mean(pops_bimep, na.rm = TRUE))^2, na.rm = TRUE)
}

for (gofr_make_idx in 1:nrow(gofr)) {
  gofr_source <- gofr[gofr_make_idx, "source"]
  gofr_grid <- gofr[gofr_make_idx, "grid"]
  gofr_resolution <- gofr[gofr_make_idx, "resolution"]
  bimep_name <- files_df[
    files_df$grid == gofr_grid & files_df$resolution == gofr_resolution & files_df$source =
    "name"
    ]
  pops_bimep <- raster::getValues(gofr_map_type[bimep_name][[1]])
  compare_map <- gofr_map_type[gofr[gofr_make_idx, "name"]][[1]]
  pops_map <- raster::getValues(compare_map)
  stopifnot(length(pops_bimep) == length(pops_map))

  # GOFR for the whole island.
  gofr$GOFR[gofr_make_idx] <- gofr_of_maps(pops_bimep, pops_map, normalize_gofr)

  # Iterate through the four admin2 by ID.
  segmented <- raster::getValues(raster::rasterize(
    admin2_st, compare_map, field = admin2_sf$OBJECTID))
  for (adm2_idx in 1:length(admin_names)) {
    sub_bimep <- pops_bimep[segmented == adm2_idx]
    sub_pops <- pops_map[segmented == adm2_idx]
    gofr[[admin_names[adm2_idx]]][gofr_make_idx] <- gofr_of_maps(sub_bimep, sub_pops, norma
  }
}
gofr
}
# Set to maps or density for original map or density surface.
gofr_map_type <- maps
gofr_absolute <- calculate_gofr(FALSE, gofr_map_type)
gofr_normalized <- calculate_gofr(TRUE, gofr_map_type)
choose <- 1:nrow(gofr_normalized)
cols <- c("grid", "resolution", "GOFR", "Baney", "Luba", "Malabo", "Riaba")
gofr_table <- rbind(gofr_absolute[choose, cols], gofr_normalized[choose, cols])
gofr_table
```

	grid	resolution	GOFR	Baney	Luba	Malabo	Riaba
HRSL coarse	HRSL	coarse	0.4138739	0.1570497	2.3945595	0.4517480	1.2952773
HRSL fine	HRSL	fine	0.9594931	1.0203838	3.1138156	0.9221886	3.1640946
GPW coarse	LandScan	coarse	0.9774469	1.0381888	1.0025690	0.9865757	1.0101944
LandScan coarse	LandScan	coarse	0.4719145	0.3712822	5.3800489	0.4870594	0.8089782
GPW fine	LandScan	fine	0.9883225	1.0139721	1.0013438	0.9944350	1.0008494
LandScan fine	LandScan	fine	0.7072126	0.8138229	1.3312524	0.7030759	0.9685300
WorldPop-C coarse	WorldPop	coarse	0.4313588	0.4136286	1.6191136	0.4439543	3.7734652
WorldPop-U coarse	WorldPop	coarse	0.7549939	0.7888236	0.9613794	0.7751246	0.9606142
WorldPop-C fine	WorldPop	fine	0.7706022	1.1294676	2.1172865	0.7279449	5.8789446
WorldPop-U fine	WorldPop	fine	0.8710373	0.9161720	0.9957948	0.8792588	0.9939277
HRSL coarse1	HRSL	coarse	0.4059734	0.1374114	0.1636434	0.4243434	0.3838233
HRSL fine1	HRSL	fine	0.9726343	1.0017457	1.0062601	0.9606814	1.4952068
GPW coarse1	LandScan	coarse	0.9717156	0.9969812	1.0004946	0.9863201	1.0034200
LandScan coarse1	LandScan	coarse	0.5455361	0.1360059	1.6583646	0.5696500	0.4578038
GPW fine1	LandScan	fine	0.9855358	0.9994530	1.0003357	0.9944164	1.0002270
LandScan fine1	LandScan	fine	0.7397548	0.7263601	0.9585947	0.7633306	0.9585455
WorldPop-C coarse1	WorldPop	coarse	0.4907176	0.3433935	0.1947156	0.4879687	0.2869291
WorldPop-U coarse1	WorldPop	coarse	0.8144712	0.9083583	0.9867411	0.7822062	0.9781008
WorldPop-C fine1	WorldPop	fine	0.7307216	0.6836563	0.7020810	0.7239107	0.7075961
WorldPop-U fine1	WorldPop	fine	0.9021504	0.9644446	0.9985091	0.8807455	0.9973660

The incoming maps are HRSL fine, WorldPop-C fine, WorldPop-U fine, GPW coarse, and LandScan coarse.

```
# This is here to show how to get the table into LaTeX. Add format = "latex" to the line be
# You'll want to run this in the terminal and copy to the document.
knitr::kable(t(gofr_table[, !names(gofr_table) %in% c("resolution")]), digits = 4)
```

Let's remind ourselves what the WorldPop-U looks like.

```
plot(maps[["WorldPop-U on WorldPop fine"]])
```

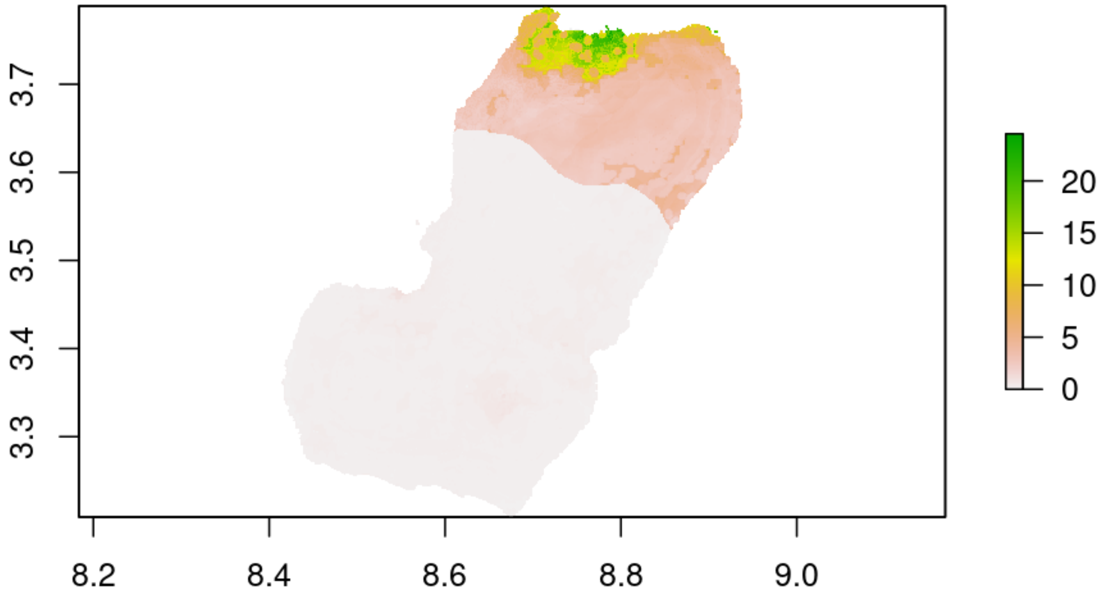

8 Map Plots

This paper looks at the maps many ways. Let's not exclude the most traditional look at how maps differ. This uses seven categories of population size, consistent among the maps.

```
library(tmap)
# I made this outline from the admin2 borders using QGis b/c the file
# made with sf::st_combine wasn't valid.
bbioko <- sf::st_transform(sf::st_read(rprojroot::find_package_root_file(
  "inst/extdata/Bioko_outline.shp")),
  raster::crs(maps[1][[1]]))
```

```
## Reading layer `Bioko_outline' from data source `/home/adolgert/dev/popbioko/inst/extdata
## Simple feature collection with 1 feature and 11 fields
## Geometry type: POLYGON
## Dimension:     XY
## Bounding box:  xmin: 434940.3 ymin: 354696.7 xmax: 493120.3 ymax: 418732.8
## Projected CRS: WGS 84 / UTM zone 32N
```

```
qleg <- FALSE
qbreaks <- c(0, 1, 10, 100, 1000, 10000, 100000)
qnames <- c("<1", "1-10", "10-100", "100-1000", "1000-10000", ">10000")
qpal <- c("#BBBBBBFF", viridisLite::viridis(length(qnames) - 1))
# raster::projection(density[["BIMEP on HRSL coarse"]]) <- "+proj=longlat +datum=WGS84 +no_
# tmap::tm_shape(bbioko) + tmap::tm_borders(col = "black", lwd = 1) +
for (set_crs_idx in 1:length(density)) {
```

```
  raster::crs(density[[set_crs_idx]]) <- utm_projection
}
qbm <- tm_shape(density[["BIMEP on HRSL coarse"]]) +
  tm_raster(title = "Population", palette = qpal, breaks = qbreaks, labels = qnames) +
  tm_layout(title = "BIMEP", frame = FALSE) +
  tm_legend(show = TRUE, legend.position = c(0.02, .01))

qwpc <- tm_shape(density[["WorldPop-C on WorldPop coarse"]]) +
  tm_raster(title = "qwpc", palette = qpal, breaks = qbreaks, labels = qnames) +
  tm_layout(title = "WorldPop-C", frame = FALSE) + tm_legend(show = qleg)

qwpu <- tm_shape(density[["WorldPop-U on WorldPop coarse"]]) +
  tm_raster(title = "qwpu", palette = qpal, breaks = qbreaks, labels = qnames) +
  tm_layout(title = "WorldPop-U", frame = FALSE) + tm_legend(show = qleg) +
  tm_compass(type="4star", size=2)

gpw <- tm_shape(density[["GPW on LandScan coarse"]]) +
  tm_raster(title = "gpw", palette = qpal, breaks = qbreaks, labels = qnames) +
  tm_layout(title = "GPW", frame = FALSE) + tm_legend(show = qleg)

qls <- tm_shape(density[["LandScan on LandScan coarse"]]) +
  tm_raster(title = "qls", palette = qpal, breaks = qbreaks, labels = qnames) +
  tm_layout(title = "LandScan", frame = FALSE) + tm_legend(show = qleg)

qhrsl <- tm_shape(density[["HRSL on HRSL coarse"]]) +
  tm_raster(title = "qhrsl", palette = qpal, breaks = qbreaks, labels = qnames) +
  tm_layout(title = "HRSL", frame = FALSE) + tm_legend(show = qleg)


quadmap <- tmap_arrange(qbm, qls, qhrsl, qwpc, gpw, qwpu, ncol = 2, nrow = 3)
tmap_save(quadmap, "islandquad.png")
```

```
## Warning: Values have found that are less than the lowest break

## Warning: Values have found that are less than the lowest break

## Warning: Values have found that are less than the lowest break

## Warning: Values have found that are less than the lowest break

## Warning: Values have found that are less than the lowest break
## Map saved to /home/adolgert/dev/popbioko/vignettes/islandquad.png
## Resolution: 2100 by 2100 pixels
## Size: 7 by 7 inches (300 dpi)
```

```
tmap_save(quadmap, "islandquad.pdf")
```

```
## Warning: Values have found that are less than the lowest break

## Warning: Values have found that are less than the lowest break

## Warning: Values have found that are less than the lowest break
```

```
## Warning: Values have found that are less than the lowest break

## Warning: Values have found that are less than the lowest break
## Map saved to /home/adolgert/dev/popbioko/vignettes/islandquad.pdf
## Size: 7 by 7 inches
```

```r
tmap_save(quadmap, "islandquad.eps")
```

```
## Warning: Values have found that are less than the lowest break

## Warning: Values have found that are less than the lowest break

## Warning: Values have found that are less than the lowest break

## Warning: Values have found that are less than the lowest break

## Warning: Values have found that are less than the lowest break
## Map saved to /home/adolgert/dev/popbioko/vignettes/islandquad.eps
## Size: 7 by 7 inches
```

```r
quadmap
```

```
## Warning: Values have found that are less than the lowest break

## Warning: Values have found that are less than the lowest break

## Warning: Values have found that are less than the lowest break

## Warning: Values have found that are less than the lowest break

## Warning: Values have found that are less than the lowest break
```

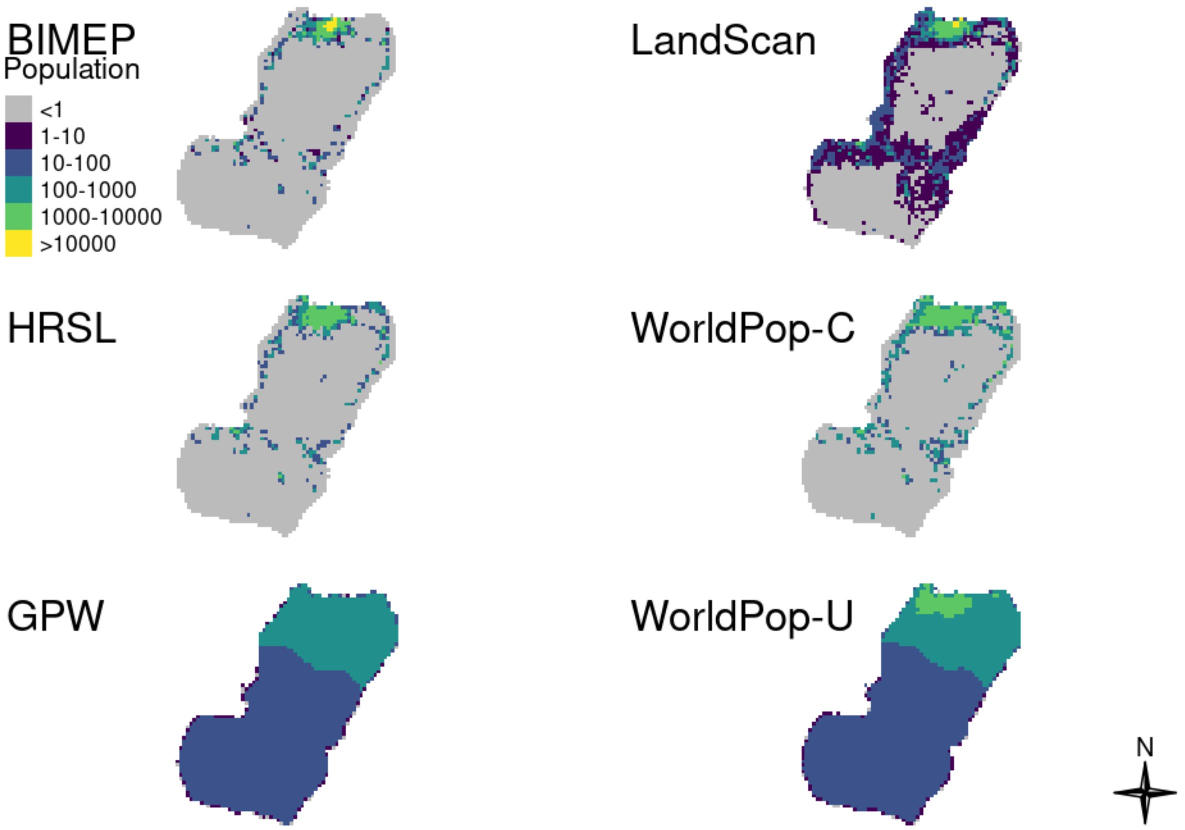

The plots may not have good spacing here, but they produce better spacing when making a PDF for final output.

```
library(tmap)
bcrop <- raster::extent(c(8.685, 8.85, 3.65, 3.80))  # for maps, not densities.
#bcrop <- raster::extent(c(440000, 490000, 350000, 424000))
original_map_names <- c(
 "BIMEP on HRSL fine",
 "HRSL on HRSL fine",
 "LandScan on LandScan coarse",
 "WorldPop-C on WorldPop fine",
 "GPW on LandScan coarse",
 "WorldPop-U on WorldPop fine"
)
short_names <- vapply(strsplit(original_map_names, " "), function(x) x[[1]], FUN.VALUE = cl
bmaps <- maps[original_map_names]
bbreaks <- c(0, 1, 5, 10, 50, 100)
bnames <- c("0-1", "1-5", "5-10", "10-50", "50-100")
#bnames[seq(2, length(bnames), 2)] <- ""
bpal <- c("#BBBBBBFF", viridisLite::viridis(length(bbreaks) - 1))
```

```
bcropped <- raster::crop(bmaps[1][[1]], bcrop)
fig2_order <- c("BIMEP", "LandScan", "HRSL", "WorldPop-C", "GPW", "WorldPop-U")
stopifnot(all(fig2_order %in% short_names))
stopifnot(all(short_names %in% fig2_order))
# Adding 0.1 is a way to put integers in the middle of the breakpoints.
small_maps <- lapply(1:length(fig2_order), function(map_idx) {
```

```r
  chosen_bmap <- which(startsWith(names(bmaps), fig2_order[map_idx]))
  crop_map <- raster::crop(bmaps[chosen_bmap][[1]], bcrop) + 0.1
  # For maps with 10x the side length.
  density_adjust <- (.3 / 360)^2 / prod(raster::res(crop_map))
  bLegend <- (map_idx == 2)
  tm_shape(crop_map * density_adjust) +
  tm_raster(title = "Population", palette = bpal, breaks = bbreaks, labels = bnames) +
  tm_legend(show = bLegend, outside=FALSE, position = c(-0.12, 0)) +
  tm_layout(title = fig2_order[map_idx], title.position = c(0.55, 0.92),
            title.bg.color = "white", title.size = 1.2,
            frame = FALSE)
})
bleg <- tm_shape(bcropped) +
  tm_raster(title = "Population", palette = bpal, breaks = bbreaks, labels = bnames) +
  tm_legend(legend.only = TRUE, position = c("center", "top"), title.size = 2, text.size =
small_maps[length(small_maps) + 1] <- bleg
```

```
## Warning in small_maps[length(small_maps) + 1] <- bleg: number of items to
## replace is not a multiple of replacement length
```

```r
small_maps[c("ncol", "nrow")] <- c(2, 3)
#closeup <- tmap_arrange(bp1, bp2, bp3, bleg, ncol = 2, nrow = 2)
# closeup <- do.call(tmap_arrange, small_maps)
sm <- small_maps
# outer.margins = c(bottom, left, top, right)
closeup2 <- tmap_arrange(sm[[1]], sm[[2]], sm[[3]], sm[[4]], sm[[5]], sm[[6]], nrow = 3, nc
tmap_save(closeup2, "closeup2.png")
```

```
## Warning: Values have found that are higher than the highest break

## Warning: Values have found that are higher than the highest break

## Map saved to /home/adolgert/dev/popbioko/vignettes/closeup2.png

## Resolution: 2100 by 2100 pixels

## Size: 7 by 7 inches (300 dpi)
```

```r
tmap_save(closeup2, "closeup2.pdf")
```

```
## Warning: Values have found that are higher than the highest break

## Warning: Values have found that are higher than the highest break

## Map saved to /home/adolgert/dev/popbioko/vignettes/closeup2.pdf

## Size: 7 by 7 inches
```

```r
tmap_save(closeup2, "closeup2.eps")
```

```
## Warning: Values have found that are higher than the highest break

## Warning: Values have found that are higher than the highest break

## Map saved to /home/adolgert/dev/popbioko/vignettes/closeup2.eps
## Size: 7 by 7 inches
```

```r
closeup2
```

```
## Warning: Values have found that are higher than the highest break
```

```
## Warning: Values have found that are higher than the highest break
```

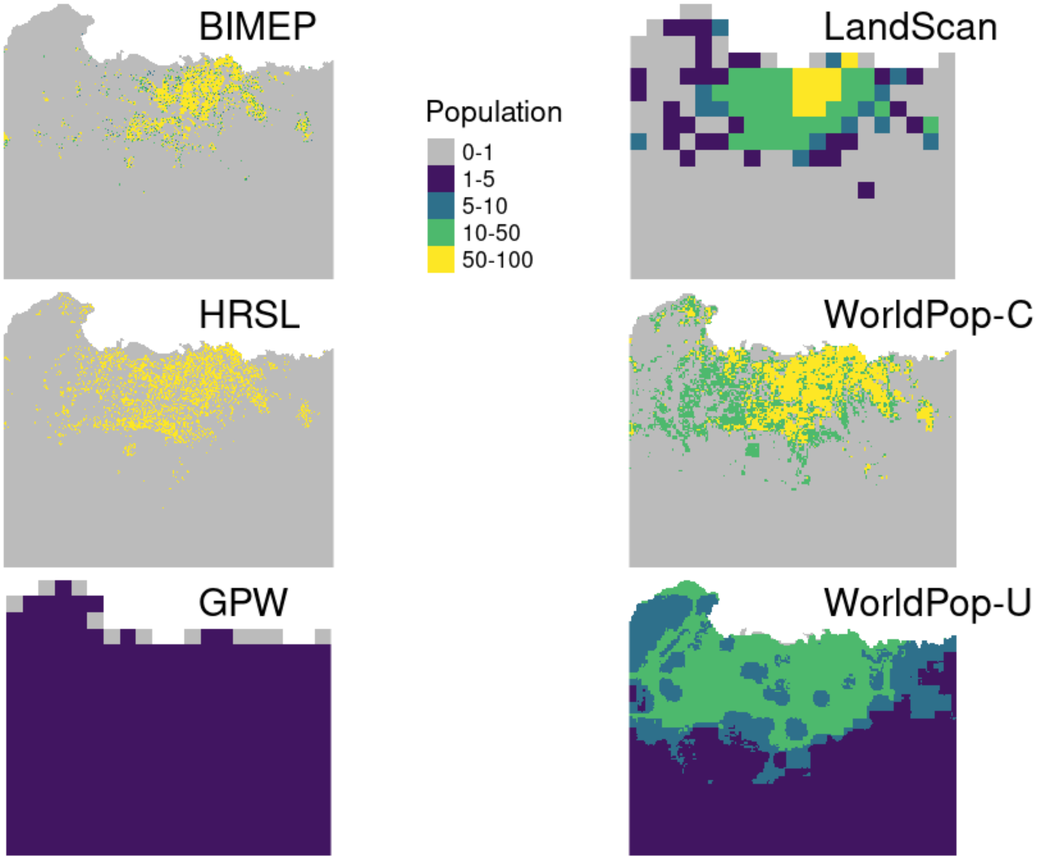

Note, for comparison, how diffuse the density map is, because the density map averages over a disc that's a square kilometer in area.

```r
bcrop <- raster::extent(c(465000, 490000, 400000, 424000))
bmaps <- density[c(3, 12, 4)]  # bimep, worldpop, hrsl
raster::plot(raster::crop(bmaps[1][[1]], bcrop))
```

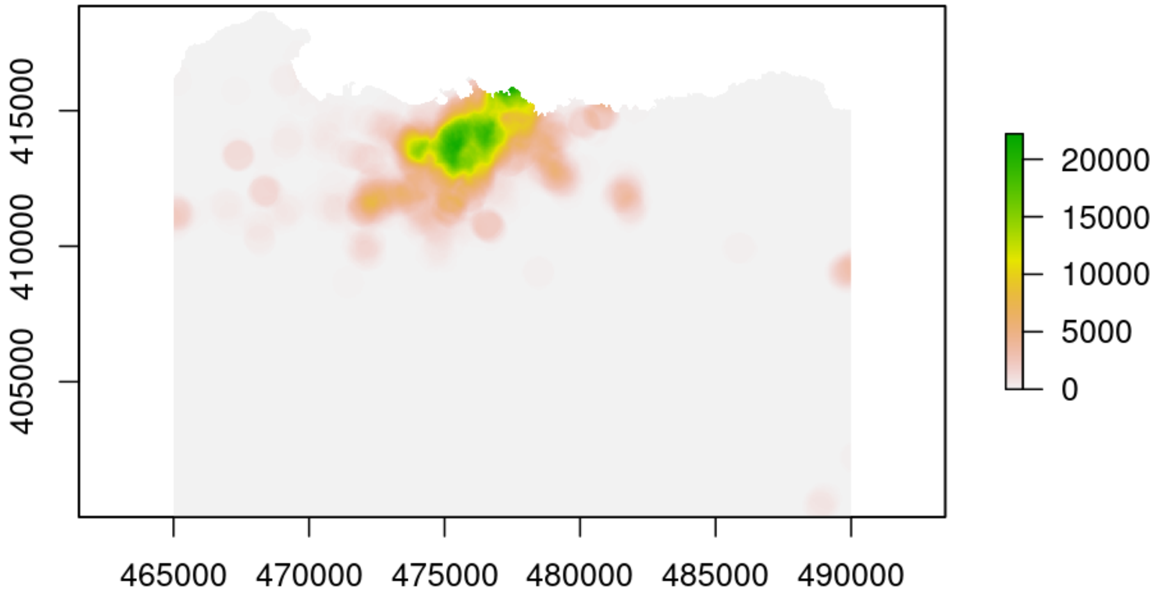

9 Figure 7: PfPR by Population

This makes the plot for figure 7. We have PfPR from a separate calculation and now ask how much population is subject to that PfPR. There are two RData sources, but we're using them to reconstruct a single result from a separate analysis of malaria parasite density, PfPR, that's referenced in the paper. We'll combine this with the population density data from above.

```
# Get the pfpr data.
source_data <- "/home/adolgert/dev/popbioko/inst/extdata/source"
load(file.path(source_data, "popData_image.RData"))
load(file.path(source_data, "pfpr_pops.RData"))
popk2 <- distinct(popk, popk$areaId, .keep_all = T)
poppr <- merge(mcprk, popk2, by = "areaId", all.x = T)
poppr <- poppr[!is.na(poppr$pop1k), ]
```

The goal is to find how many people live with a particular PfPR, so we focus on how to get density.

```
library(st)
```

```
## Loading required package: sda
## Loading required package: entropy
## Loading required package: corpcor
## Loading required package: fdrtool
```

```r
library(sf)
areas_sf <- sf::st_as_sf(areasgr)
areas_df <- sf::st_set_geometry(areas_sf, NULL)
stopifnot(all(sort(areas_df[[1]]) == areas_df[[1]]))
library(raster)
```

```
##
## Attaching package: 'raster'

## The following object is masked from 'package:nlme':
##
##     getData

## The following objects are masked from 'package:spatstat.geom':
##
##     area, rotate, shift

## The following object is masked from 'package:dplyr':
##
##     select
```

```r
sources <- c(
  "BIMEP on HRSL fine", "HRSL on HRSL fine", "LandScan on LandScan fine",
  "WorldPop-C on WorldPop fine", "WorldPop-U on WorldPop fine", "GPW on LandScan fine")
aligned <- list()
for (align_source in sources) {
  base_name <- strsplit(align_source, " ")[[1]][1]
  save_name <- paste0(base_name, "_gr.Rda")
  if (file.exists(save_name)) {
    aligned[[align_source]] <- local({
      load(save_name)
      bimep_gr
    })
  } else {
    density_map <- density[[align_source]]
    bimep_gr <- raster::extract(
      density_map, areasgr, cellnumbers = TRUE, method = "bilinear",
      na.rm = TRUE, fun = mean)
    save(bimep_gr, file = save_name)
    aligned[[align_source]] <- bimep_gr
  }
}
on_bgrid <- lapply(aligned, function(pop_matrix) {
  pm <- as.array(pop_matrix)
  pm[is.nan(pm)] <- 0  # Also an artifact.
  pm[pm < 0] <- 0  # largest negative is 10^-11. From bilinear interpolation.
  pm
  })
on_bgrid[["areaId"]] <- areas_df[["FID"]]
on_bgrid_df <- do.call(data.frame, on_bgrid)
poppr <- merge(mcprk, on_bgrid_df, by = "areaId", all.x = TRUE)
poppr <- poppr[complete.cases(poppr),]
row.is.finite <- apply(poppr, 1, function(x) any(is.finite(x)))
poppr <- poppr[row.is.finite, ]
```

```
prBYh_cdf <- function(Pf, H, B = 100) {
  xx <- seq(0, 1, length.out = B + 1)
  cdf <- 0 * xx
  for (i in 0:B) {
    ix <- which(Pf <= xx[i + 1])
    cdf[i + 1] <- ifelse(length(ix) == 0, 0, sum(H[ix]))
  }
  cbind(xx, cdf)
}

prBYh_pdf <- function(Pf, H, B = 100) {
  xx <- prBYh_cdf(Pf, H, B)
  xmid <- (xx[-1, 1] + xx[-B - 1, 1]) / 2
  pdfdist <- diff(xx[, 2])
  cbind(xmid, pdfdist)
}

cdfPfPlot <- function(Pf, H, B = 100, clr = "black", llwd = 2) {
  xx <- prBYh_cdf(Pf, H, B)
  plot(xx[, 1], xx[, 2],
    type = "l", xlab = "PfPR", ylab = "Population fraction",
    main = "A", lwd = llwd, col = clr, xlim = c(0, .45),
    ylim = c(0, 1)
  )
}

cdfPfLines <- function(Pf, H, B = 100, clr = "red", llwd = 2) {
  xx <- prBYh_cdf(Pf, H, B)
  lines(xx[, 1], xx[, 2], lwd = llwd, col = clr)
}

pdfPfPlot <- function(Pf, H, B = 100, llwd = 2, clr = "black") {
  xx <- prBYh_pdf(Pf, H, B)
  plot(xx[, 1], xx[, 2], col = clr, type = "l", lwd = llwd, xlim = c(0, .45), ylim = c(0, [illegible]
}

pdfPfLines <- function(Pf, H, B = 100, clr = "red", llwd = 2) {
  xx <- prBYh_pdf(Pf, H, B)
  lines(xx[, 1], xx[, 2], col = clr, lwd = llwd)
}

PfPR <- poppr$pfpr
sources <- c("BIMEP", "HRSL", "LandScan", "WorldPop.C", "WorldPop.U", "GPW")
pop_cols <- vapply(sources, function(x) names(poppr)[startsWith(names(poppr), x)], characte[illegible]

pfprFigure <- function() {
  par(mfrow = c(1, 2))
  for (cdf_idx in seq(pop_cols)) {
    BB <- 80
    pops <- poppr[[pop_cols[cdf_idx]]]
    normed_data <- pops / sum(pops)
    short_name <- gsub("\\.", "-", names(pop_cols)[cdf_idx])
    if (cdf_idx == 1) {
```

```
      cdfPfPlot(PfPR, normed_data, BB, clr = colors[short_name])
    } else {
      cdfPfLines(PfPR, normed_data, BB, clr = colors[short_name])
    }
  }

  short_names <- character(0)
  colors_used <- character(0)
  for (pdf_idx in seq(pop_cols)) {
    pops <- poppr[[pop_cols[pdf_idx]]]
    normed_data <- pops / sum(pops)
    short_name <- gsub("\\.", "-", names(pop_cols)[pdf_idx])
    if (pdf_idx == 1) {
      pdfPfPlot(PfPR, normed_data, BB, clr = colors[short_name])
    } else {
      pdfPfLines(PfPR, normed_data, BB, clr = colors[short_name])
    }
    short_names <- c(short_names, short_name)
    colors_used <- c(colors_used, colors[short_name])
  }
  legend("topright",
    legend = short_names, col = colors_used,
    lty = 1, lwd = 2, cex = 1
  )
}

pfprFigure()
```

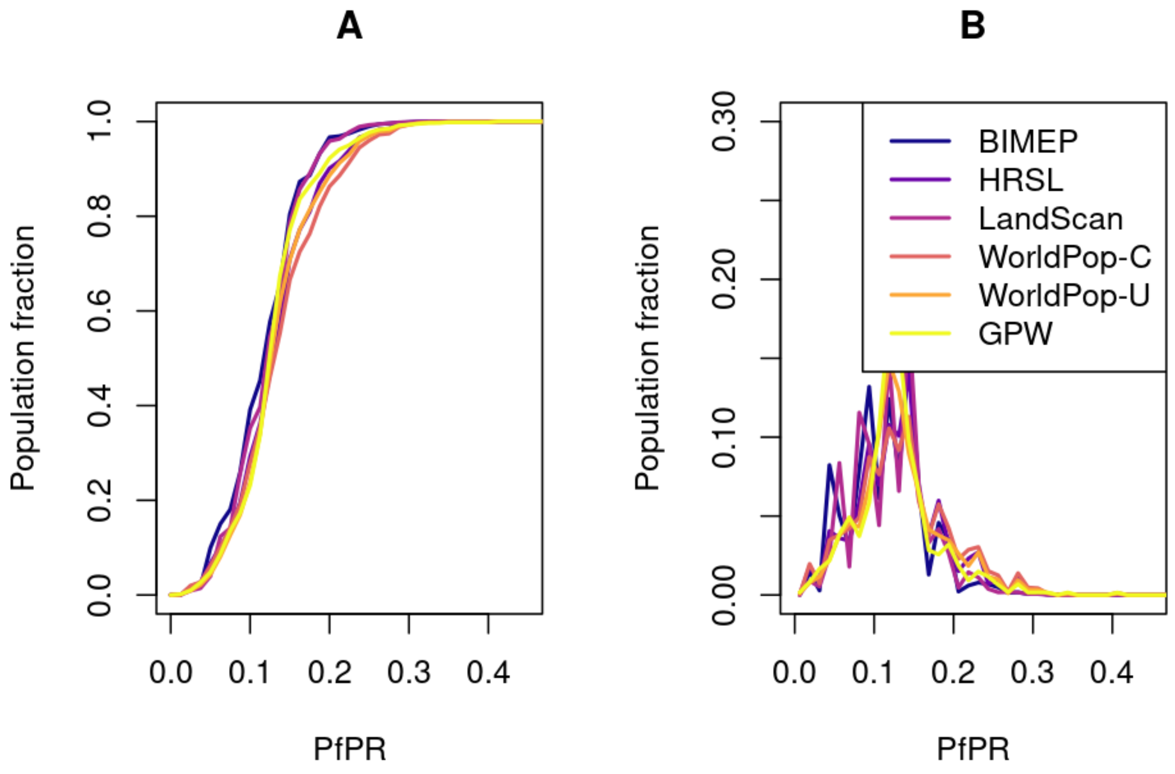


```
popbioko::save_plot(pfprFigure, "PRbypop_.pdf")
#pdf("PRbypop_.pdf", width = 10, height = 5)
#pfprFigure()
#a <- dev.off()
```

GATHER Compliance

Contents

1 Overview **1**

2 Checklist **1**

Bibliography **3**

From git@github.com:dd-harp/population_comparison_bioko.git on Sat May 22 11:14:59 2021, generated by adolgert.

1 Overview

This is the GATHER compliance checklist for the paper, *Measuring the accuracy of gridded human population density surfaces: a case study in Bioko Island, Equatorial Guinea* (Stevens et al. 2016). This checklist summarizes data and methods in order to be transparent. It's intended for global health audiences but is general enough to offer some clarity for this paper.

2 Checklist

1. Define the indicator(s), populations (including age, sex, and geographic entities), and time period(s) for which estimates were made. - The study area is Bioko Island, Equatorial Guinea. The Bioko Island data is 2018. LandScan data is 2018. WorldPop Unconstrained is the 2018 version. WorldPop Constrained is for 2020, so we adjusted it with caveats. HRSL is current, which makes it a 2019 version, but it assigns numbers from census data, which is older. The age and sex are all ages and all sexes. It's a total population count.
2. List the funding sources for the work. - Bill and Melinda Gates Foundation BMGF OPP1110495 for the analysis done here. That's separate from funding for the Bioko Island Malaria Elimination Project that collected this data. BIMEP lists donors on its website: https://www.mcdinternational.org/donors.
3. Describe how the data were identified and how the data were accessed. - The BIMEP GPS-level data was critical to a previous collaboration among IHME, BIMEP, and other organizations in order to estimate malaria prevalence on Bioko Island.

Gridded datasets were included if a) they were publicly available b) they cover most of Africa c) for the last five years, and d) they include spatial information at a subnational level.

- Included datasets
  - LandScan from ORNL
  - WorldPop's Constrained and Unconstrained datasets
  - Facebook and CIESIN's High resolution settlement layer
  - Gridded Population of the World (GPWv4.11).
- Excluded datasets of note
  - ESRI's World Population Estimate, because it is commercial.
  - Global Rural-Urban Mapping Project (GRUMPv1), because it covers 1990-2000 only.
  - JRC's European GHS Population Grid, because it is superseded by GPW.

  – U.S. Census Bureaus's country grids (Demobase). This covers only three countries: Haiti, South Sudan, and Pakistan.

4. Specify the inclusion and exclusion criteria. Identify all ad-hoc exclusions. The gridded maps chosen are all those public maps which mostly cover Africa. The HRSL, in particular, excludes South Sudan for ethical reasons, but otherwise covers Africa. We excluded the Gridded Population of the World because it is directly from census data, so it will be constant across Bioko, by design.
5. Provide information about all included data sources and their main characteristics.
   a) BIMEP GPS data. This data is not public because it's house-level GPS coordinates. Source is the Bioko Island Malaria Elimination Project, part of

   MCDI. Their contact information is MCD International Office, 8401 Colesville Rd, Suite 425, Silver Spring, MD 20910, P: 301-562-1920, F: 301-562-1921, Email: mcdi@mcd.org.

   b) WorldPop. Direct download. ftp://ftp.worldpop.org.uk/GIS/Population/Individual_countries/GNQ/Equatorial_Guinea_100m_Population.7z
   c) HRSL. Direct download: https://ciesin.columbia.edu/repository/hrsl/hrsl_gin_v1.zip
   d) LandScan. You have to register on their website. The data is called "LandScan Global 2018." https://landscan.ornl.gov/landscan-datasets
   e) Shapefile for Bioko Island, available at https://zenodo.org/record/4781344#.YKkwvRJlARU. This shapefile was created by the BIMEP program.
6. Identify data that have potentially important biases. - The BIMEP data covers 88% of households, not 100%. The missing 12% isn't geographically biased. It's from people not being home when they checked several times. People tend to move seasonally for work, so undercounting people in houses is less of an undercount for the actual number of people. We chose to use the data as is.
7. Describe and give sources for any other data inputs. - There is a shapefile for Bioko island, constructed by island administration. We could equally well use the GADM shapefile for Equatorial Guinea. We use this to define island boundaries. We noticed that the island boundaries for LandScan and WorldPop had more area near the Southwest shoreline. It was less than one percent, and we went with the Bioko map because it is supported by GPS coordinates of houses.
8. Provide all input datasets in a format which can be extracted. - We've handled this by providing the code we used to do the calculation. That code will automatically download all of the data except the BIMEP GPS data and the Bioko shapefile. It's unfortunately not enough for someone else to rerun this code, but it is the exact code that created the outputs.
9. Provide a conceptual overview of the data analysis method. The paper text is about the analysis method.
10. Provide a detailed description of all steps. The paper is exactly about the math of the steps, but it's also provided in the code we make available.
11. Describe how candidate models were evaluated and the final models selected. This seems more for statistical models. We did do some work to evaluate what should be the goodness-of-fit ratio. We settled on the final choice of sum-of-squared-errors divided by gold standard variance because it most resembles relative error, which is (observed - measured) / measured. So that made sense. This work is in the repository as "gofr_comparison.pdf."
12. Provide results of an evaluation of model performance. - Contained in the paper's results section.
13. Describe methods of calculating uncertainty of the estimates. - There is a section of the supplement that looks at uncertainty in point estimates due to exact grid location. Each gridded map serves as a realization of a population. The map comparison is a demonstration of uncertainty.

14. State how analytic or statistical source code can be accessed. - The code is in a public repository at this web address: https://github.com/dd-harp/population_comparison_bioko.
15. Provide published estimates in a file from which it can be extracted. - These are on the github, for the tables. They are in the vignettes directory under summary_statistics.csv, gofr_table.csv, and accuracy.csv.
16. Report quantitative measures of the uncertainty of the estimates. We put those into the summary statistics table. That's our best measure, by translating the grid.
17. Interpret results in light of existing evidence. - In the paper.
18. Discuss limitations on estimates. - In the paper's last section.

Bibliography

Stevens, Gretchen A, Leontine Alkema, Robert E Black, J Ties Boerma, Gary S Collins, Majid Ezzati, John T Grove, et al. 2016. "Guidelines for Accurate and Transparent Health Estimates Reporting: The Gather Statement." *The Lancet* 388 (10062): e19–e23.
