# Supplementary material for: Measuring the accuracy of gridded human population density surfaces: A case study in Bioko Island, Equatorial Guinea
Source: PLoS One. 2021 Sep 1;16(9):e0248646. doi: 10.1371/journal.pone.0248646 (PMC8409626; doi:10.1371/journal.pone.0248646)
Supplement: S1 File — (DOCX) [file pone.0248646.s002.docx]

Authors' Contributions

BF: Conceptualization, Supervision, Methodology, Validation, and Writing Original Draft Preparation. CAG: Data curation, Project Administration, and Writing Review and editing. GAG: Data Curation, and Writing – Review & Editing. SLW: writing, review, and editing. JMS, JNMO, OTD and JOON: Resources. SIH: Writing Review and Editing. DLS: Conceptualization, Supervision, Methodology, Formal Analysis, and Writing – Editing and Review, AJD: Software, investigation, validation, visualization, writing - original draft.

Funding

This work was supported by the Bill and Melinda Gates Foundation grant OPP1110495 – DLS. The funders had no role in study design, data collection and analysis, decision to publish, or preparation of the manuscript.

Brendan Fries: South and Central Africa ICEMR, Johns Hopkins Bloomberg School of Public Health, Baltimore, MD, USA. & Spatial Science for Public Health Center, Johns Hopkins Bloomberg School of Public Health, Baltimore, MD, USA.

Bfries2@jh.edu

Carlos A Guerra: Medical Care Development International, Silver Spring, MD, USA.

Carguegal@gmail.com

Guillermo A García: Medical Care Development International, Silver Spring, MD, USA.

ggargcia@mcd.org

Sean L Wu: Divisions of Biostatistics & Epidemiology, University of California, Berkeley, CA, USA.

[Sluwu89@berkely.edu](mailto:Sluwu89@berkely.edu)

Jordan M Smith: Medical Care Development International, Malabo, Equatorial Guinea.

Jmsmith@mcd.org

Jeremías Nzamio Mba Oyono: Medical Care Development International, Malabo, Equatorial Guinea.

jnzamio@mcd.org

Olivier T Donfack: Medical Care Development International, Malabo, Equatorial Guinea.

Otresor@mcd.org

José Osá Osá Nfumu: Medical Care Development International, Malabo, Equatorial Guinea. & Ministry of Health and Social Welfare, Malabo, Equatorial Guinea.

josa@mcd.org

Simon I Hay: Department of Health Metrics Sciences, School of Medicine, University of Washington, Seattle, WA, USA. & Institute for Health Metrics and Evaluation, University of Washington, Seattle, WA, USA.

Sihay@uw.edu

David L Smith: Department of Health Metrics Sciences, School of Medicine, University of Washington, Seattle, WA, USA. & Institute for Health Metrics and Evaluation, University of Washington, Seattle, WA, USA.

Smitdave@gmail.com

Andrew J Dolgert: Institute for Health Metrics and Evaluation, University of Washington, Seattle, WA, USA.

[adolgert@uw.edu](mailto:adolgert@uw.edu)

Keywords: malaria, malaria prevalence, geographic information system, population health, demographics, mapping, epidemiology, population density, urban, rural, accuracy evaluation.
